# Iron-catalysed, general and operationally simple formal hydrogenation using Fe(OTf)_3_ and NaBH_4_ †
†We dedicated this paper to Dr Stuart Warren on the occasion of his 75th birthday.
‡
‡Electronic supplementary information (ESI) available. See DOI: 10.1039/c4ob00945b



**DOI:** 10.1039/c4ob00945b

**Published:** 2014-06-10

**Authors:** Alistair J. MacNair, Ming-Ming Tran, Jennifer E. Nelson, G. Usherwood Sloan, Alan Ironmonger, Stephen P. Thomas

**Affiliations:** a School of Chemistry , University of Edinburgh , Joseph Black Building , West Mains Road , Edinburgh EH9 3JJ , UK . Email: stephen.thomas@ed.ac.uk ; Fax: +44 (0)131 650 6453; b Research and Development , GlaxoSmithKline , Gunnelswood Road , Stevenage SG1 2NY , UK

## Abstract

An operationally simple and environmentally benign formal hydrogenation protocol has been developed using highly abundant iron(iii) salts and an inexpensive, bench stable, stoichiometric reductant, NaBH_4_, in ethanol, under ambient conditions. This reaction has been applied to the reduction of terminal alkenes (22 examples, up to 95% yield) and nitro-groups (26 examples, up to 95% yield). Deuterium labelling studies indicate that this reaction proceeds *via* an ionic rather than radical mechanism.

## Abstract 2

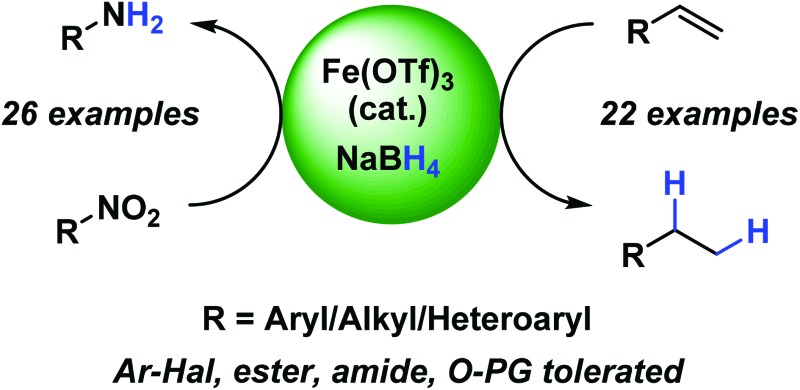
An operationally simple and environmentally benign formal hydrogenation protocol has been developed using a highly abundant iron(iii) salt and an inexpensive, bench stable, stoichiometric reductant, NaBH_4_, in ethanol, under ambient conditions.

## Introduction

The hydrogenation of apolar and polar functionalities is routine in both academia and industry for the production of fine and bulk chemicals.^1^ Highly operationally simple hydrogenation methods using heterogeneous (*e.g.* Pd/C–H_2_) or homogeneous (*e.g.* Ru/NEt_3_–HCO_2_H) systems have allowed the broadest possible user base to exploit this reaction.^2^ To date, the most commonly used methods require precious or semi-precious transition metal complexes or finely divided powders.^1^

Iron-based catalysts offer several advantages over more traditional ‘noble’ metal systems due to the high abundance, long-term availability,^3^ low cost and low toxicity of iron.^4^ On an industrial scale, heterogeneous iron catalysts have been widely exploited; however the use of soluble iron catalysts is considerably less well developed.^5^

Iron-catalysed alkene reductions have been reported however many systems suffer from the need for: elevated temperatures; high hydrogen pressure; chemical activation or are superstoichiometric in iron.^6^ Although several well defined and highly active homogeneous iron complexes for catalytic hydrogenation have been developed,^7^ notably by Chirik,^8^ these catalysts and pre-catalysts are highly air- and moisture-sensitive, so have not seen widespread adoption. On a small scale, the use of hydrogen gas has numerous drawbacks, particularly with safe storage and handling. These can be circumvented by the use of an inexpensive, bench-stable, solid hydrogen source. NaBH_4_ is air- and moisture stable and produced on kilotonne scale annually.^9^

**Scheme 1:**
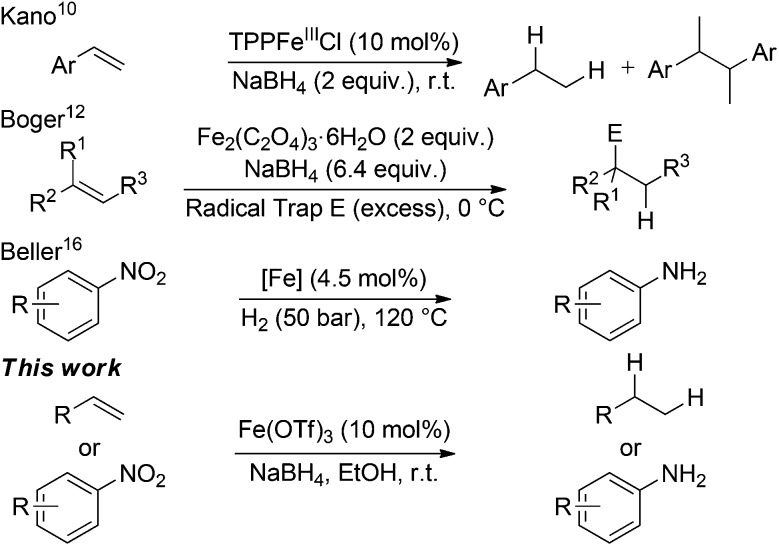
Iron-catalysed reductions and reductive functionalisations. TTP = tetraphenylporphyrinato. [Fe] = iron phenanthroline complex pyrolysed onto a carbon support.

Ashby used LiAlH_4_ in conjunction with stoichiometric amounts of transition metal halides, including FeCl_3_ and FeCl_2_, to reduce 1-octene.^10^ Kano reported a biomimetic reduction of styrene derivatives using an iron-porphinato complex and NaBH_4_ however reductive homocoupling of radical species was a major side-reaction.^11^ Recently, Boger reported the hydrofunctionalisation of alkenes mediated by superstoichiometric iron(iii) salts and NaBH_4_.^12^ In the absence of an electrophile, it was found that tertiary alkenes were hydrogenated (Scheme 1).^12*a*^

Along with alkene hydrogenation, the reduction of nitro-arenes to aniline derivatives represent another high-value industrial process for the preparation of a wide range of synthetic precursors, including; dyes, pharmaceuticals, agrochemicals and polymers.^13^ Iron-catalysed hydrogenation of nitroarenes is well established using iron(0) carbonyl precursors acting *via* a cohort of *in situ* generated iron species.^14^ Beller has developed a number of homogeneous iron-catalysed nitroarene reductions^15^ and recently carbon-supported heterogeneous systems using either N_2_H_4_·6H_2_O or H_2_ as the stoichiometric reductant (Scheme 1).^16^

NaBH_4_ is a poor reducing agent for nitro-groups under ambient conditions, although it has been used in the presence of palladium, nickel, copper catalysts for the reduction of nitro-groups to amines.^17^ Additionally, Sakaki and co-workers have reported the use of NaBH_4_ and porphyrinatoiron complexes for the reduction of a limited number of nitroarenes.^18^

Herein we report an iron-catalysed, NaBH_4_-mediated reduction procedure that is capable of reducing both alkene and nitroarene functionalities.

## Results and discussion

Alkene reduction was first investigated and successful ‘hydrogenation’ of 4-phenyl-1-butene **1a**, to the alkane **2a**, was found using stoichiometric (Table 1, entries 1–4) and substoichiometric (entries 5–10) amounts of simple, commercially available, iron salts in the presence of NaBH_4_.^19^

**Table 1:** Initial screen of activity of iron salts for the reduction of 4-phenyl-1-butene^*a*^

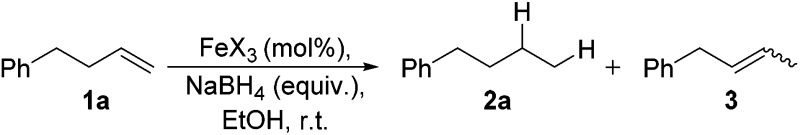					
Entry	FeX_2/3_	FeX_2/3_ (mol%)	Equiv. NaBH_4_	Yield^*b*^ (%)	
				**2a**	**3**
1	FeCl_3_	100	2	15	2
2	FeBr_3_	100	2	42	1
3	Fe(OTf)_3_	100	1	19	3
4	Fe(OTf)_3_	100	2	91	9
5	FeCl_3_	10	2	91	5
6	FeCl_3_ ^*c*^	10	2	89	6
7	Fe(OTf)_3_	10	2	90	10
**8**	**Fe(OTf)_3_** ^*d*^	**10**	**1.5**	**90**	**10**
9	Fe(OTf)_3_ ^*e*^	1	2	47	7
10	Fe(OTf)_2_	10	2	11	0
11	HOTf	10	2	6	0^*f*^
12	NaOTf	10	2	4	0^*g*^

^*a*^Conditions: 0.50 mmol 4-phenyl-1-butene, *n* mol% iron(iii) salt, *n* equiv. NaBH_4_, EtOH (2 ml), r.t., 16 h.

^*b*^Yield measured by ^1^H NMR of the crude reaction product using 1,3,5-trimethoxybenzene as internal standard.

^*c*^>99.99% purity.

^*d*^6 h.

^*e*^48 h.

^*f*^75% starting material recovered.

^*g*^80% starting material recovered.

**Scheme 2:**
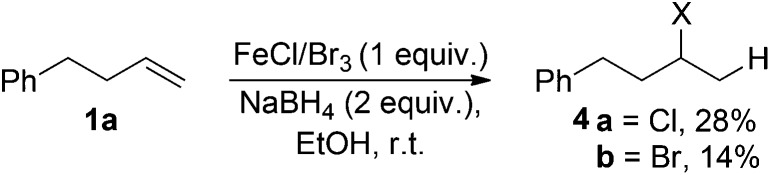
Formation of halogenated side products, X = Cl/Br.

Iron(iii) chloride, bromide and triflate supported the reduction (entries 1–4); however when stoichiometric FeCl_3_ or FeBr_3_ were used, (3-chlorobutyl)benzene **4a** and (3-bromobutyl)benzene **4b** were obtained as side-products respectively. This was presumably as a result of radical formation, followed by halide abstraction from the iron salt (Scheme 2).^20^

Use of Fe(OTf)_3_ prevented the halogenation reaction and in addition, it was found that Fe(OTf)_3_ gave the shortest reaction times.^21^ At a 10 mol% iron loading, the quantity of NaBH_4_ could be lowered to 1.5 equivalents and the reaction time reduced to 6 hours, without decreasing reaction yield (entry 8), however in all cases, it was found the some isomerisation to the internal alkene **3** was observed. An attempt to reduce the catalyst loading to 1 mol% gave considerably diminished yields, even after 48 h (entry 9).

The catalytic activity of iron was attested to by high purity FeCl_3_ (>99.99%) showing equal catalytic activity (entry 6) to the reagent grade salts.^22^ Additionally in the absence of iron, no reduction of the alkene was observed: triflic acid and sodium triflate (entries 11 and 12) were not catalytically active; only the starting material **1a** was recovered.

Presumably due to the high solubility of NaBH_4_ in these solvents, successful reduction reactions were achieved in methanol, 1-butanol, 2-butanol and acetonitrile, however the highest yields were obtained in ethanol.^19^ Along with the sustainability and low toxicity of ethanol, makes it the favoured reaction solvent.

With the optimal conditions of Fe(OTf)_3_ (10 mol%), NaBH_4_ (150 mol%) in ethanol, the substrate scope of the formal hydrogenation was investigated. The developed system was found to be chemoselective for the reduction of terminal alkenes (Table 2). Reductions in the presence of aryl halides showed no protodehalogenation^23^ except in the case of aryl bromide **1d** where 18% of the protodehalogenated product was observed (Table 2, entries 2–4).

**Table 2:** Scope and limitation of the iron-catalysed, hydride-mediated reduction^*a*^

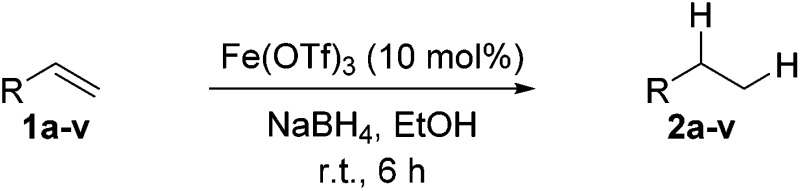			
Entry	Substrate	Product	Yield^*b*^ (%)
1	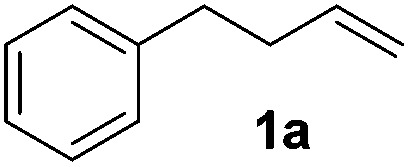	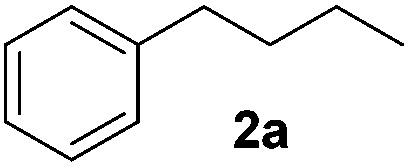	90 (83)
	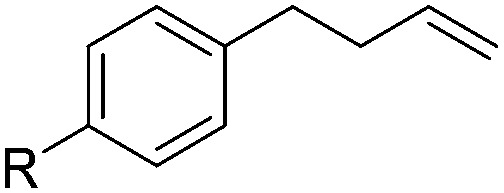	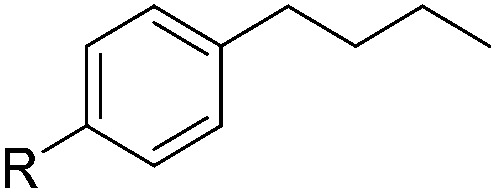	
2	R = F (**1b**)	**2b**	92 (79)
3	R = Cl (**1c**)	**2c**	93 (77)
4	R = Br (**1d**)	**2d**	78 (71)^*c*^
	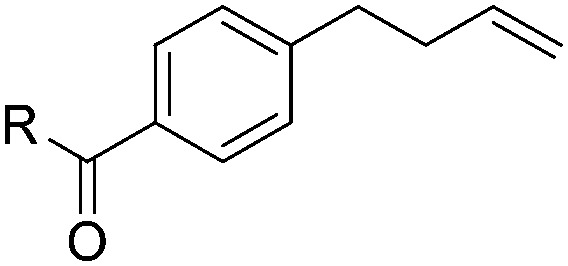	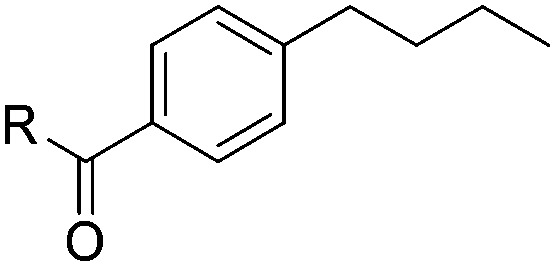	
5	R = OH (**1e**)	**2e**	25
6	R = OMe (**1f**)	**2f**	95 (94)
7	R = NH^*t*^Bu (**1g**)	**2g**	73
8	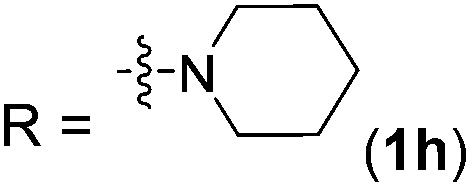	**2h**	92 (87)
9	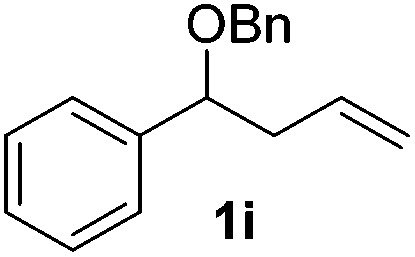	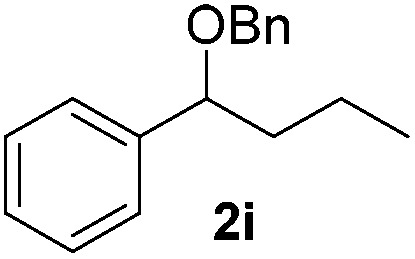	50 (50)
10	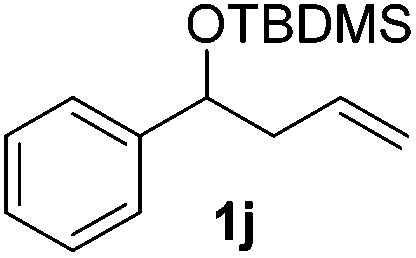	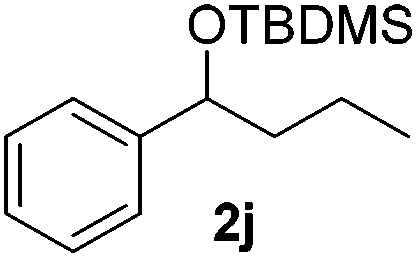	56
11	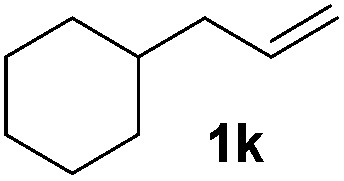	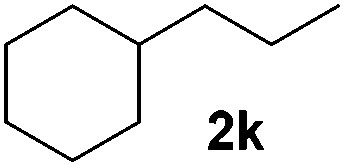	>95 (69)
12	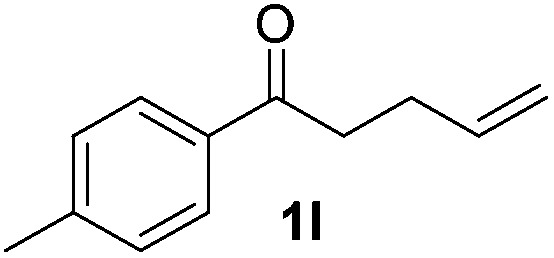	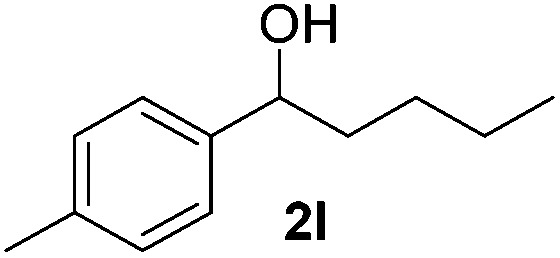	22
13	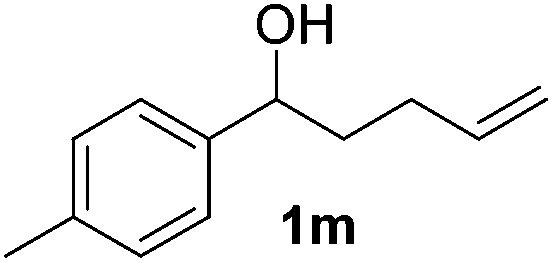	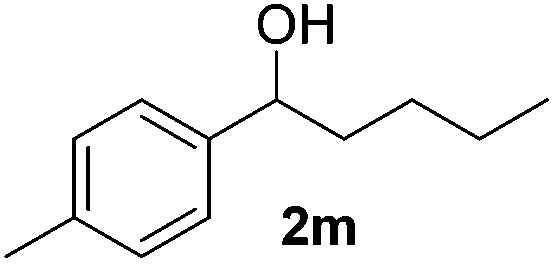	10
	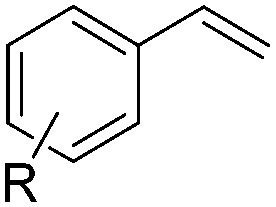	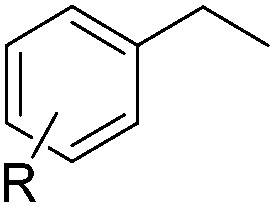	
14	R = 4-Cl (**1n**)	**2n**	58^*d*^
15	R = 4-^*t*^Bu (**1o**)	**2o**	55 (45)^*d*^
16	R = 4-OMe (**1p**)	**2p**	56 (46)^*d*^
17	R = 3-CF_3_ (**1q**)	**2q**	50^*d*^
18	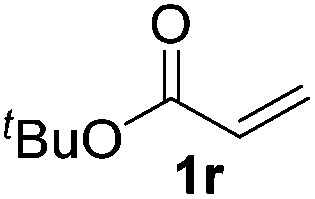	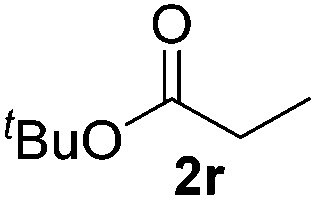	>95 (73)
19	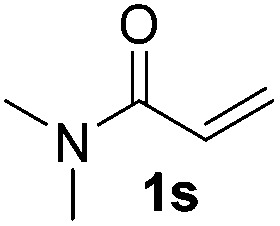	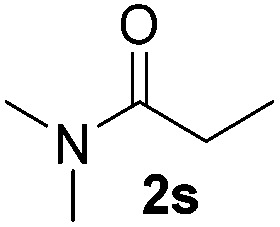	75
20	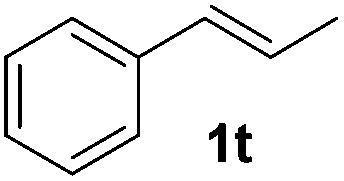	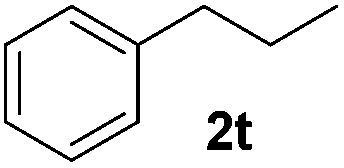	3
21	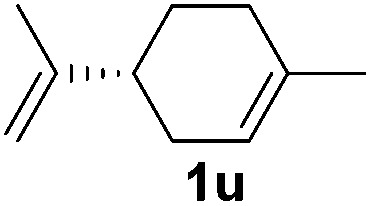	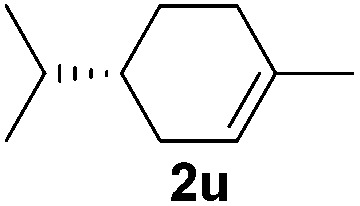	0
22	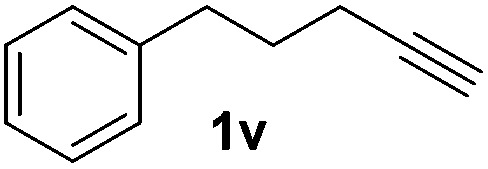	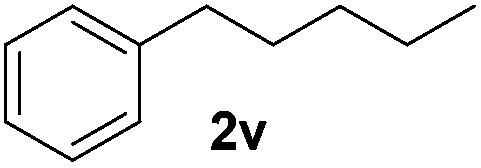	7, 34^*e*^

^*a*^Conditions: 1 mmol alkene, 10 mol% Fe(OTf)_3_, EtOH (4 ml), 1.5 equiv. NaBH_4_, r.t., 6 h.

^*b*^Determined by ^1^H NMR using 1,3,5-trimethoxybenzene as internal standard. Isolated yield in parentheses.

^*c*^18% phenylbutane **2a** also recovered.

^*d*^Conditions: 1 mmol alkene, 10 mol% Fe(OTf)_3_, EtOH (4 ml), 2 equiv. NaBH_4_, r.t., 18 h.

^*e*^20 equiv. NaBH_4_.

Despite previous reports of the reduction of esters and amides with NaBH_4_ in MeOH,^24^ chemoselective alkene reduction was observed for substrates being both ester and amide functionalities (entries 6–8). Although a carboxylic acid functionalised substrate was poorly tolerated (entry 5), reduction of 4-phenyl-1-butene **1a** in the presence of acetic acid, using excess NaBH_4_, was successful. Despite the lability of benzyl protecting groups under conventional hydrogenation conditions, both benzyl and silyl ethers were conserved during alkene reduction (entries 9 and 10).

Although the reduction was carried out in ethanol, inclusion of an alcohol or ketone in the alkene substrate diminished reduction yields (entries 12 and 13). Styrene derivatives were successfully reduced; however longer reaction times and higher quantities of NaBH_4_ were required and yields were generally lower than the alkyl analogues (entries 14–17). In contrast to the work of de Vries using iron nanoparticles,^25^ acrylate and acrylamide derivatives were chemoselectively reduced at the alkene (entries 18 and 19). The reaction was highly selective for the reduction of unsubstituted terminal alkenes; only trace reduction of β-methyl styrene **1t** was observed and neither the internal nor 1,1-disubstituted alkenes of (+)-limonene **1u** underwent reduction (entries 20 and 21).^26^ Attempts to extend the reaction scope to the terminal alkyne; 5-phenyl-butyne **1v**, resulted in a poor yield of alkane, even with excess NaBH_4_ (entry 22). The reduction of 4-phenyl-1-butene in the presence of 10 mol% diphenylacetylene resulted in a reduced yield of phenylbutane (15%) and no evidence of reduction of the diphenylacetylene.

During the development of the alkene ‘hydrogenation’, the reduction of the nitro-group of 3-nitrostyrene was observed to occur competitively with the reduction of the alkene. Using nitrobenzene as a model substrate, simple iron salts were investigated for catalytic activity in the reduction of the nitro-group to primary amines. FeCl_3_ offers an inexpensive and readily available iron(iii) source and good reactivity was found with increased NaBH_4_ loading (Table 3, entries 1–3). The use of high-purity FeCl_3_ (≥99.99%) again did not change the observed reactivity, (entry 4). However, returning to Fe(OTf)_3_ gave higher conversions to aniline (entry 6), and allowed reaction times to be reduced to 4 h.

**Table 3:** Optimisation of nitroarene reduction^*a*^

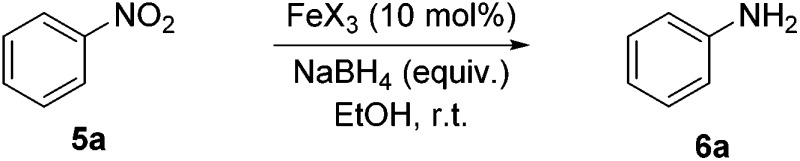				
Entry	FeX_2/3_	NaBH_4_ equiv.	*t* (h)	Conversion^*b*^ (%)
1	FeCl_3_	2	18	15
2	FeCl_3_	4	18	51
3	FeCl_3_	20	18	88
4	FeCl_3_ ^*c*^	20	18	90
5	FeCl_2_	20	18	62
6	Fe(OTf)_2_	20	18	60
7	Fe(OTf)_3_	20	18	99
8	Fe(OTf)_3_	10	18	32
**9**	**Fe(OTf)_3_**	**20**	**4**	**99**
10	BF_3_·Et_2_O	20	4	0^*d*^
11	AlCl_3_	20	4	0^*e*^
12	HOTf	20	4	1^*f*^

^*a*^Conditions: 0.5 mmol 4-phenyl-1-butene, 10 mol% iron salt, NaBH_4_, ethanol (4 ml), r.t.

^*b*^Conversion measured by ^1^H NMR.

^*c*^>99.99% purity.

^*d*^57% starting material recovered.

^*e*^31% starting material recovered.

^*f*^78% starting material recovered.

Even using the apparently more active salt, Fe(OTf)_3_, it was found that the quantity of NaBH_4_ could not be reduced without diminishing conversion to the product. In the absence of an iron salt, no reduction of nitrobenzene to aniline was observed, irrespective of the amount of NaBH_4_ used. Lewis acids; BF_3_ and AlCl_3_, were ineffective as catalysts (entries 10 and 11) and the use of triflic acid (entry 12) also resulted in only starting material being recovered.

Using these conditions, substrate scope was investigated. *o*-, *m*-, *p*-Methyl nitrobenzene, and even the sterically hindered 2,6-dimethyl nitrobenzene were all successfully reduced (Table 4, entries 2–5). Nitroarenes bearing electron-withdrawing (–CF_3_) and electron-donating (–OMe) substituents were both tolerated (entries 6–10). Nitro-groups were successfully reduced in the presence of aryl-chloride and fluoride substituents without protodehalogenation (entries 11–14), however, 4-bromo-nitrobenzene **5o** was reduced to both 4-bromoaniline and to the proto-dehalogenated product aniline (entry 15).

**Table 4:** Scope and limitation of the iron-catalysed, hydride-mediated reduction^*a*^

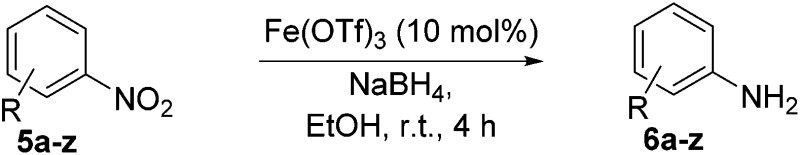							
Entry	Substrate	Product	Yield^*b*^ (%)	Entry	Substrate	Product	Yield^*b*^ (%)
1	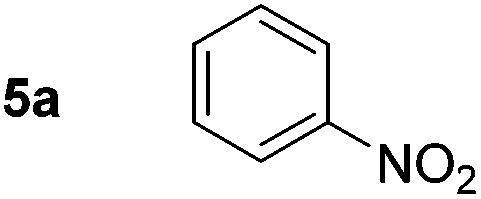	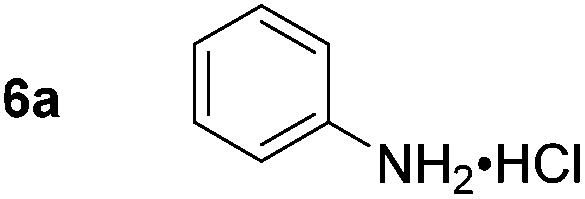	90 (80)^*c*^	14	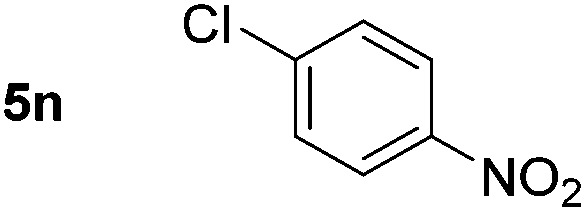	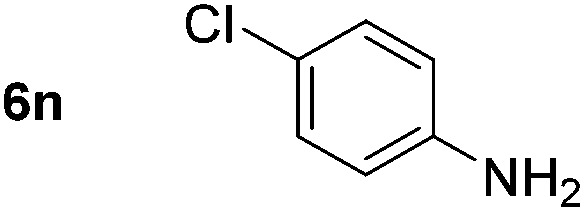	80 (47)
2	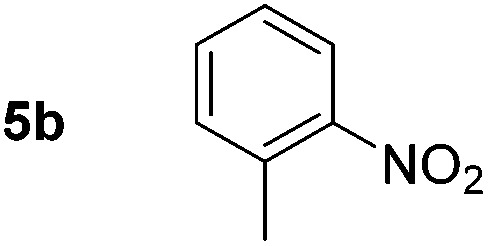	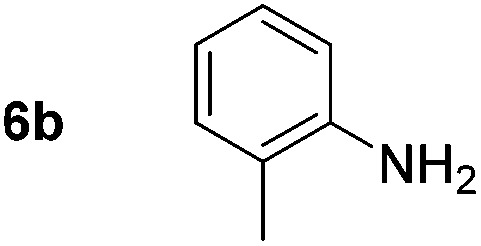	80^*d*^ (80)	15	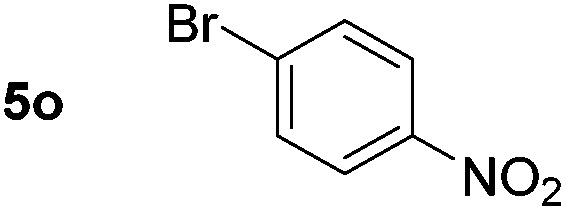	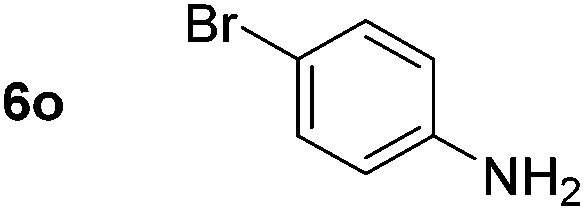	51 (51)^*e*^
3	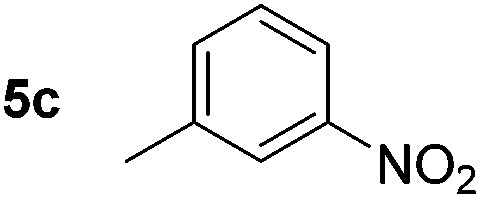	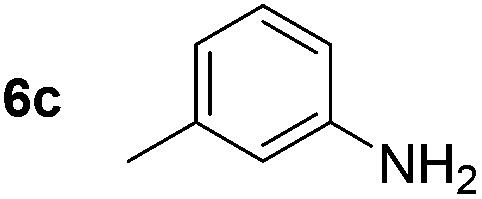	61^*d*^ (49)	16	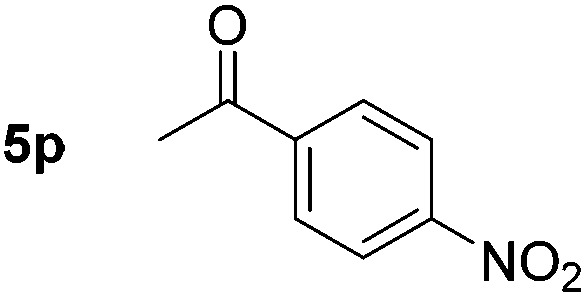	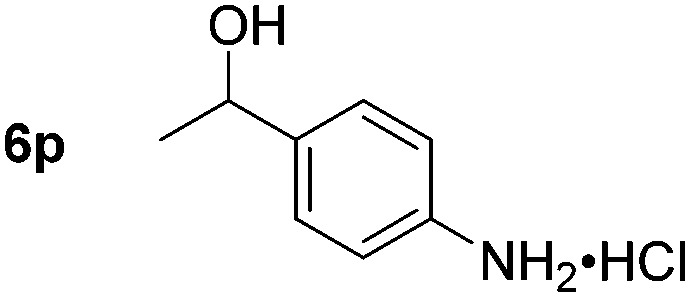	68 (15)
4	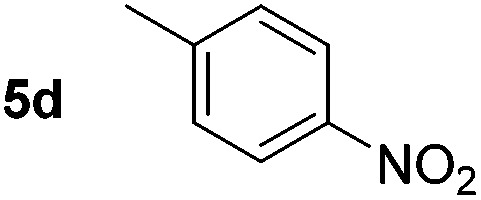	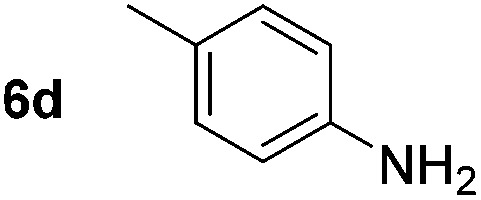	73 (66)	17	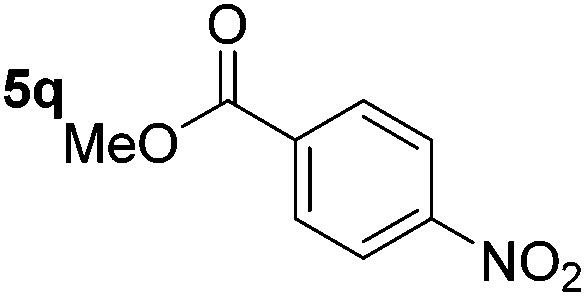	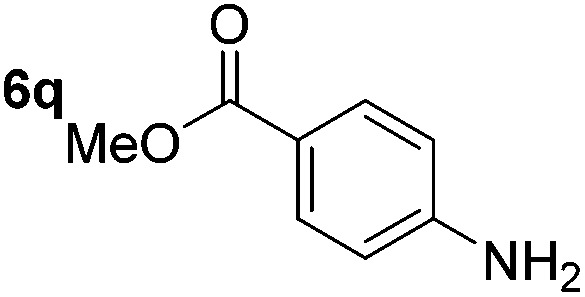	87 (80)
5	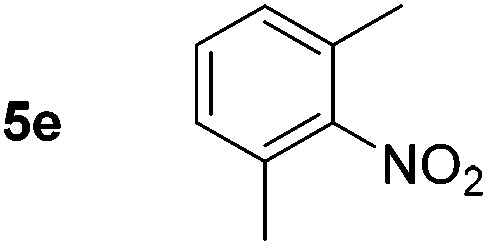	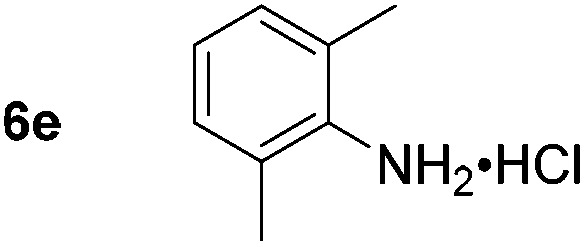	79 (59)^*c*^	18	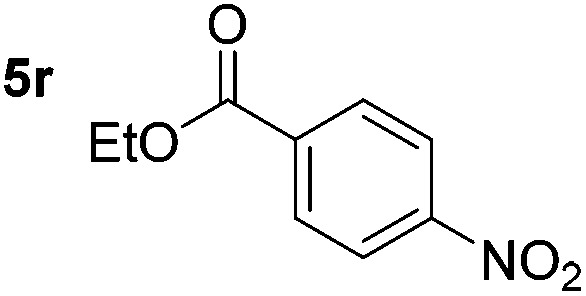	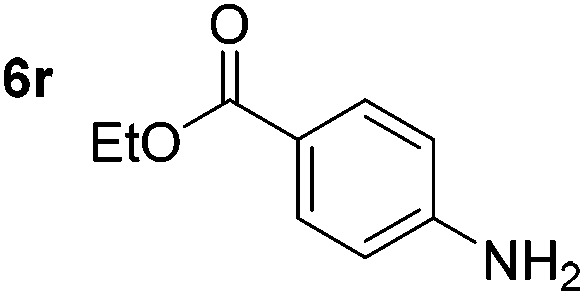	93 (28)
6	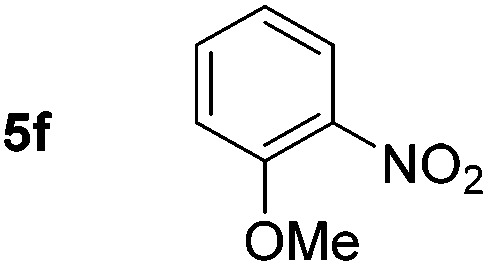	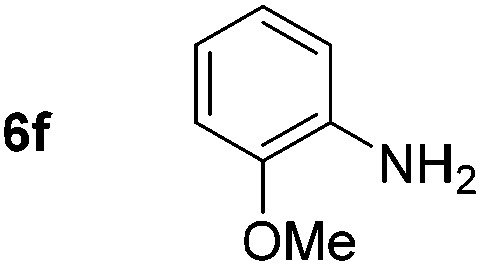	76^*d*^ (75)	19	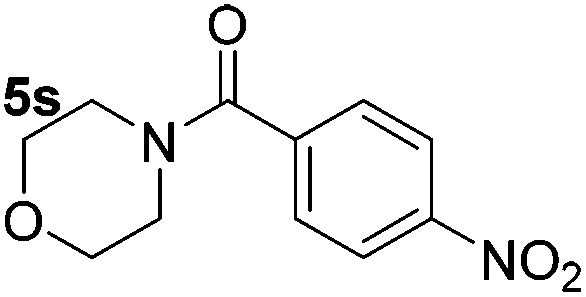	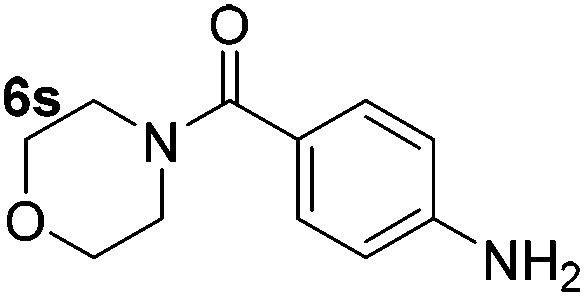	80 (32)
7	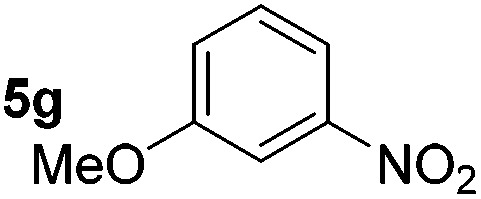	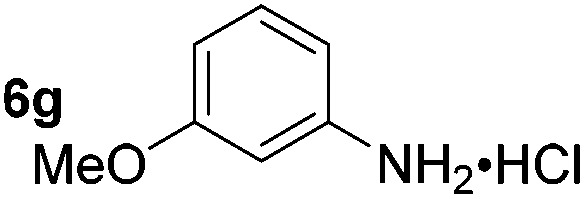	81 (68)^*c*^	20	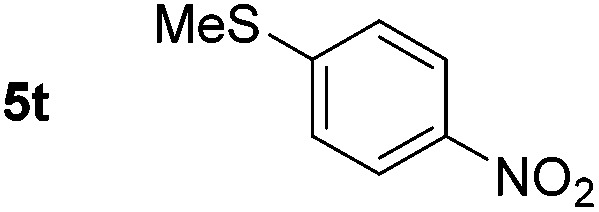	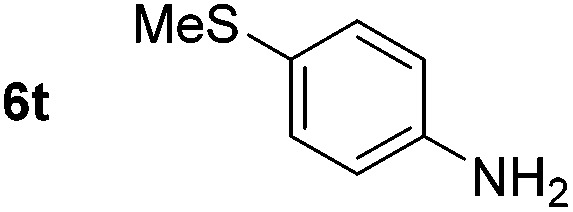	>95 (76)
8	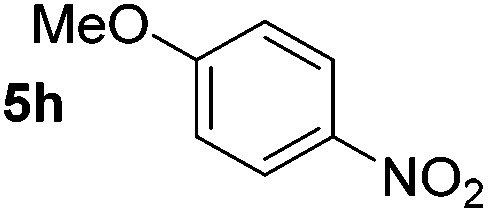	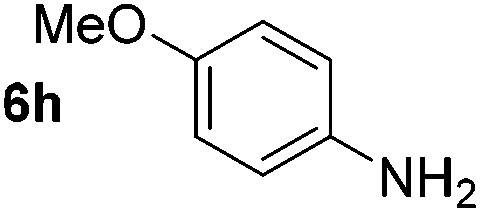	68 (24)	21	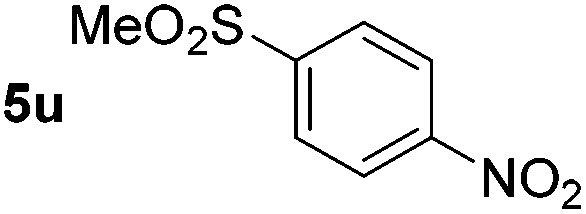	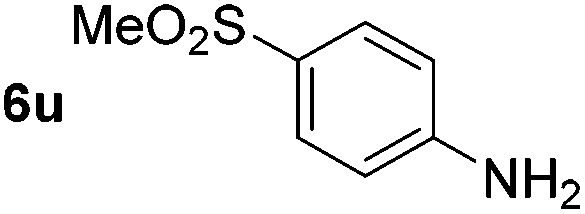	(53)
9	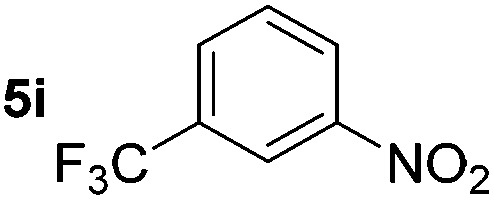	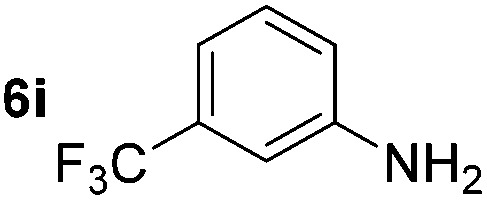	82^*d*^ (55)	22	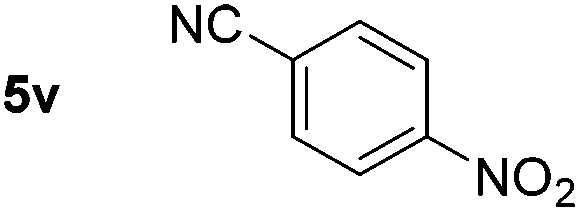	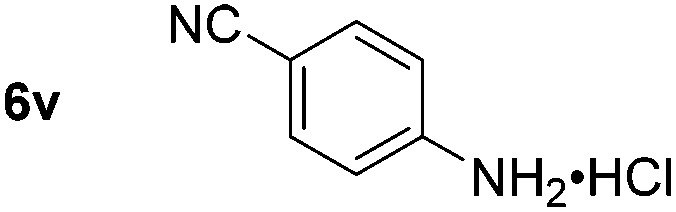	82 (77)^*c*^
10	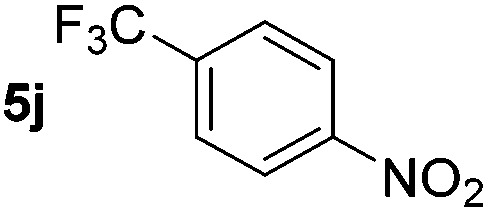	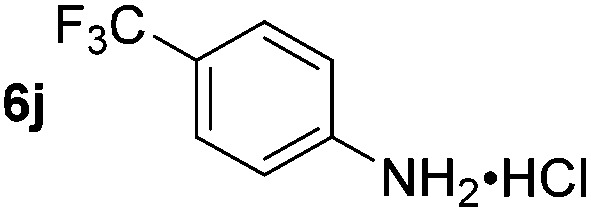	83 (76)^*c*^	23	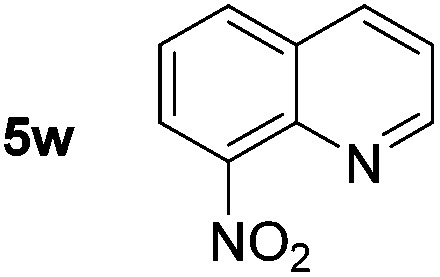	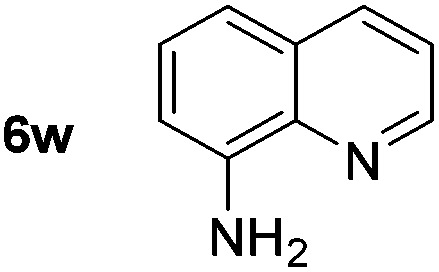	54 (51)
11	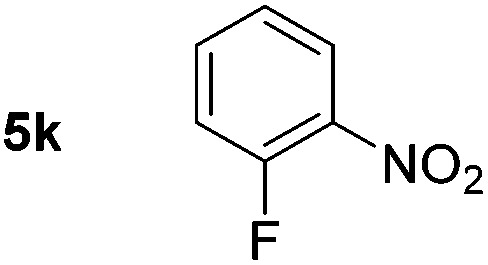	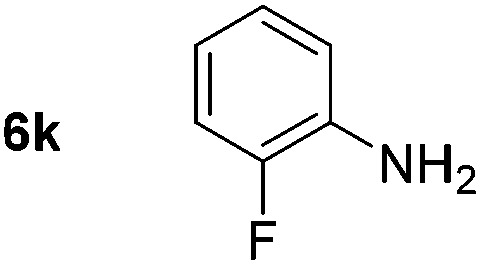	73^*d*^	24	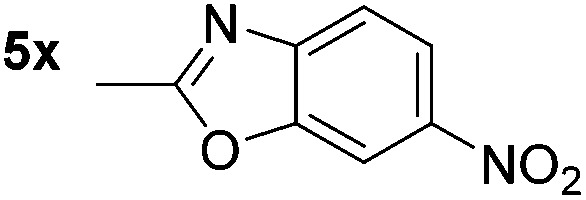	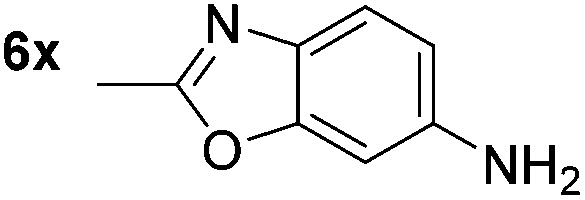	33 (24)
12	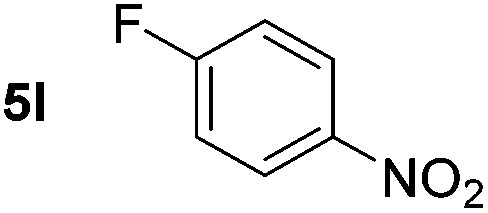	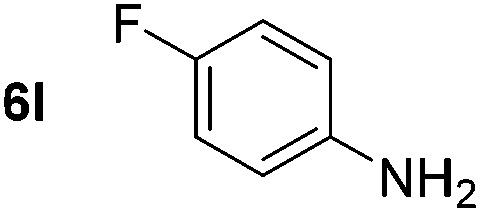	87^*d*^	25	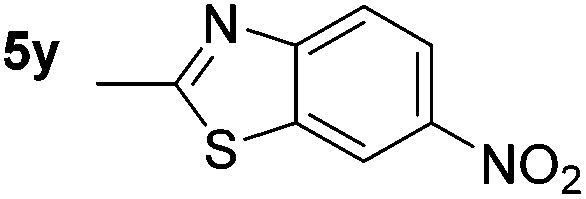	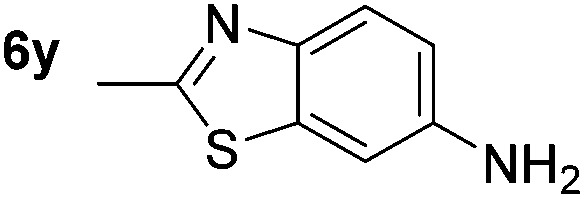	60 (56)
13	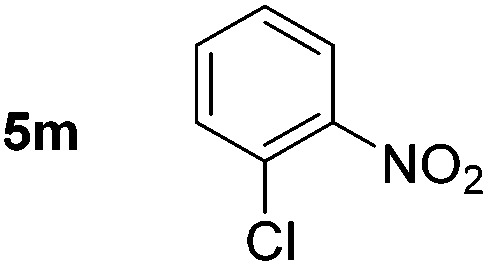	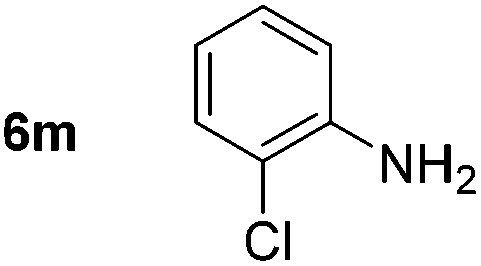	70^*d*^ (17)	26	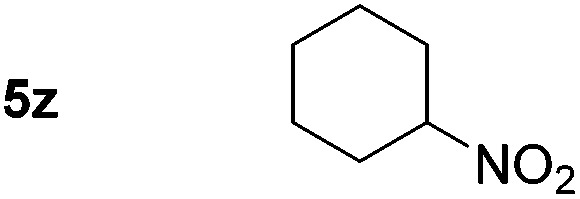	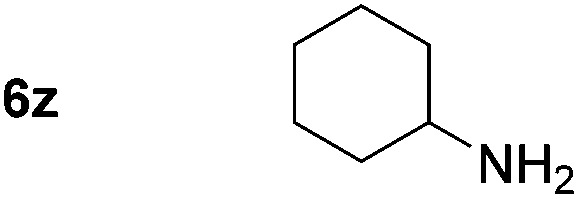	20^*f*^

^*a*^Conditions: 0.5 mmol nitroarene, 10 mol% FeOTf_3_, EtOH (4 ml), 20 equiv. NaBH_4_, r.t., 4 h.

^*b*^Yield determined by ^1^H NMR using 1,3,5-trimethoxybenzene as internal standard. Isolated yield in parentheses.

^*c*^Isolated as the HCl salt.

^*d*^1,2-Dichloroethane used as internal standard.

^*e*^9% aniline also recovered.

^*f*^Conditions: 50 mol% FeOTf_3_, 30 equiv. NaBH_4_.

Chemoselective nitro-group reduction was observed in the presence of ester and amide functionalities (entries 17–19). The synthesis of the analgesic benzocaine **6r** from the corresponding nitroarene showcases the utility of this methodology. Perhaps unsurprisingly, a substrate bearing a ketone **5p** showed poor chemoselectivity with the carbonyl being reduced in addition to the nitro-group (entry 16).

*p*-(Methylthio)-aniline **5t** was successfully produced from the corresponding nitroarene in good yield (entry 20). The corresponding methylsulfonyl substituted nitroarene **5u** was also successfully reduced, albeit with lower isolated yield (entry 21). 8-Nitroquinoline **5w** was successfully reduced to 8-aminoquinoline (entry 23). Interestingly, treatment of nitro-substituted benzoxazole **5x** and benzothiazole **5y** derivatives with NaBH_4_ in the absence of an iron salt exclusively gave the reductively ring-opened product. However, in the presence of Fe(OTf)_3_, only the chemoselective reduction of the nitro-group was observed (entries 24 and 25). Aliphatic nitro-groups were also reduced by the Fe(OTf)_3_/NaBH_4_ system (entry 26), however increased loadings of both the catalyst and stoichiometric reductant were required.

Two contrasting mechanisms have been proposed for previously reported iron-catalysed, NaBH_4_-mediated, alkene reductions. We sought to gain insight into which of the following mechanisms is operating in our developed reaction conditions. Kano proposed the addition of an iron-hydride to the alkene, followed by proton abstraction from ethanol.^11*a*^ In contrast, Boger proposed that both hydrogen atoms originated from sodium borohydride.^12*a*^ Additionally, NaBH_4_ has been shown to reduce iron(ii/iii) salts to a range of nanoparticulate or low oxidation-state iron and iron/boron species.^25,27^ While the formation of nanoparticles cannot be ruled out, the lack of stabilisers or an induction period would appear to suggest against these being the active catalytic species. In order to investigate the origin of the added hydrogen, and gain insight into the mode of operation of the low-valent catalyst, a series of deuterium incorporation experiments were carried out.

**Scheme 3:**
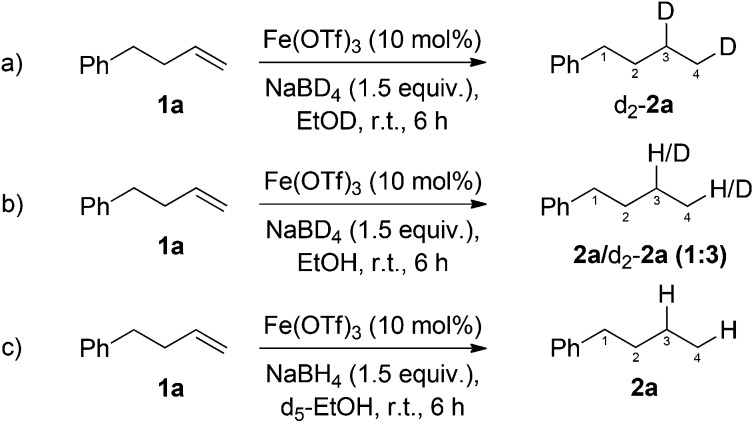
Deuterium labelling studies for the investigation of the mechanism of iron-catalysed, NaBH_4_ mediated, alkene reduction.

Reduction of 4-phenyl-1-butene **1a** using NaBD_4_ and d_1_-ethanol gave exclusively the dideuterated alkane d_2_-**2a** (Scheme 3a). In line with previous reports of deuterium exchange between NaBD_4_ and alcoholic solvents,^28^ performing the reduction with NaBD_4_ and ethanol gave a mixture of deuterated and non-deuterated alkanes (Scheme 3b). In both cases deuterium was incorporated in both C3 and C4 positions of the alkane. In order to probe the existence of a radial intermediate, d_5_-EtOH was used as the reaction solvent to probe radical abstraction from the CD_2_OH position, however, no deuterium incorporation was observed (Scheme 3c). This suggests an ionic, rather than radical mechanism.

## Conclusions

In conclusion, a single, general, operationally simple and highly applicable protocol for the formal hydrogenation of apolar (alkene) and polar (nitro-) functionalities has been developed using a simple iron salt as catalyst. Using Fe(OTf)_3_ (10 mol%) and NaBH_4_ as the stoichiometric reductant, a wide range of functionalised and unfunctionalised alkenes and aryl- and alky nitro-groups have been successfully hydrogenated under operationally simple, environmentally benign reaction conditions.
