# Supplementary material for: Iron-catalysed, general and operationally simple formal hydrogenation using Fe(OTf)3 and NaBH4
Source: Org Biomol Chem. 2014 Jun 10;12(28):5082–8. doi: 10.1039/c4ob00945b (PMC4295815; doi:10.1039/c4ob00945b)
Supplement: Supplementary file 1 [file OB-012-C4OB00945B-s001.pdf]

Electronic Supplementary Material (ESI) for Organic & Biomolecular Chemistry.
This journal is © The Royal Society of Chemistry 2014

Iron-Catalysed, General and Operationally Simple Formal Hydrogenation using $Fe(OTf)_3$ and $NaBH_4$

Supporting Information

Alistair J MacNair[a], Ming-Ming Tran[a], Jennifer E. Nelson[a], George U. Sloan[a], Alan Ironmonger[b], Stephen P. Thomas[a]

[a]School of Chemistry, University of Edinburgh, Joseph Black Building, West Mains Road, Edinburgh EH9 3JJ
E-mail: stephen.thomas@ed.ac.uk
[b]Research and Development, GlaxoSmithKline, Gunnelswood Road, Stevenage SG1 2NY

Contents

General Experimental	S2
Preparation of Alkene Derivatives	S3
Preparation of Nitro- Derivatives	S5
General Procedure for the Reduction of Alkene Derivatives	S7
Table S1. Screen of activity of stoichiometric iron salts for the reduction of 4-phenyl-1-butene	S8
Table S2. Catalytic activity of iron salts for the reduction of 4-phenyl-1-butene	S9
Table S3. Catalytic activity of iron salts for the reduction of *trans*-*β*-methylstyrene	S10
ICP-MS Analysis	S11
Screening of Alkene Substrates (Table 2)	S12
General Procedure for the Reduction of Nitro- Groups	S17
Table S6. Catalytic activity of iron salts for the reduction of nitrobenzene	S18
Screening of Nitro- Functionalised Substrates (Table 4)	S19
NMR Spectra	S27
Deuterium Labelling Experiments	S70
References	S73

General Experimental

All air and moisture sensitive reactions were carried out either using standard vacuum line and Schlenk techniques, or in a glovebox with a purified nitrogen atmosphere. All solvents for air- and moisture sensitive techniques were obtained from an anhydrous solvent system (Innovative Technology), except in the case of ethanol, which was distilled from absolute ethanol over magnesium. All glassware was cleaned using base (KOH, ^{i}PrOH) and acid ($HCl_{(aq)}$) baths.

All ^{1}H, ^{2}D and ^{13}C spectra were obtained on Bruker Avance III 400 and 500 MHz spectrometers or on a Bruker Avance I 600 MHz spectrometer. All spectra were obtained at ambient temperature. The chemical shifts (δ) and coupling constants (*J*) were recorded in parts per million (ppm) and Hertz (Hz) respectively. ^{1}H and ^{13}C multiplicities and coupling constants are reported where applicable. Spectra were recorded relative to the residual solvent residual peak ($CDCl_3$, 7.27 ppm, 77.00 ppm).

Iron(III) triflate was purchased from Sigma Aldrich Co. LLC, iron(III) trifluoromethanesulfonate, 90% (product number 708801, lot #SHBB6947V). Iron(III) chloride was purchased from Sigma Aldrich Co. LLC, iron(III) chloride, anhydrous, powder, ≥ 99.99% trace metals basis (product number 45164-9, batch #MKBG2975V). Sodium borohydride was purchased from Sigma Aldrich Co. LLC, sodium borohydride powder ≥ 98% (product number 452882, lot #STBC6399V). All reagents were used as supplied.

Flash chromatography was performed on silica gel (Merck Geduran Si 60). Petroleum spirit refers to petroleum ether distillate obtained at 40-60 °C. Thin layer chromatography was performed on aluminium backed silica plates (Merck 60 F_{254}). Catalytic reactions were assayed by Gas Chromatography Mass Spectrometry (GCMS) by comparison with authentic samples.
Injector temp.; 50 °C for 3 min; ramps 5 °C/min to 80 °C; hold for 2 min; ramps 45 °C/min to 300 °C; hold for 2 min.

Infra-red spectra were recorded on a Shimadzu IRAffinity-1 spectrometer (serial no. A213749). Melting points were determined using a Gallenkamp melting point apparatus and are uncorrected.

Preparation of Alkene Derivatives

4-(3-buten-1-yl)-benzoic acid, methyl ester (1f)

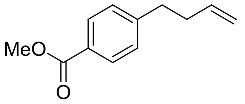

A solution of 4-(3-butenyl)-benzoic acid (0.528 g, 3.00 mmol) and sulphuric acid (10 drops) in methanol (15 mL) under a nitrogen atmosphere was heated under reflux for 18 hours. Aqueous sulphate buffer solution (15 mL) was added and the organic phase was extracted with ethyl acetate (3 × 10 mL), washed with brine, dried over $MgSO_4$ and concentrated *in vacuo*. Following flash column chromatography (80:20 hexane:diethyl ether), 4-(3-butenyl)-benzoic acid methyl ester was isolated as a colourless oil (0.400 g, 2.10 mmol, 70%). δ_H (500 MHz, $CDCl_3$) 7.98 (dt, *J* = 8.3 Hz, 1.8 Hz, 2H), 7.28 (ap d, 2H), 5.91-5.81 (m, 1H), 5.10-4.99 (m, 2H), 3.93 (s, 3H), 2.79 (t, *J* = 7.5 Hz, 2H), 2.42 (ap q, *J* = 7.9 Hz, 2H). δ_C (125 MHz, $CDCl_3$) 167.2, 147.4, 137.5, 129.7, 128.5, 127.9, 115.4, 52.0, 35.4, 35.1.

Data in accordance with that previously reported.[1]

1-Benzyloxy-1-phenyl-3-butene (1i)

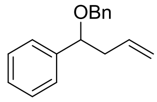

A solution of 4-phenyl-1-buten-4-ol (0.425 g, 2.90 mmol) in anhydrous tetrahydrofuran (5 ml) was added dropwise to solution of sodium hydride (0.120 g, 3.00 mmol) in anhydrous tetrahydrofuran (10 ml) being stirred under an atmosphere of nitrogen at 0 °C. The reaction mixture was stirred for 1 hour, then benzyl bromide (0.598 g, 3.50 mmol) was added dropwise and the reaction mixture was allowed to warm to room temperature. After 3 hours triethylamine (0.354 g, 3.50 mmol) was added and the reaction mixture concentrated *in vacuo*. The reaction mixture was diluted with saturated NH_4Cl solution (10 mL) with CH_2Cl_2 (10 mL). The organic phase was extracted with diethyl ether (3 × 10 mL), washed with brine, dried over $MgSO_4$, and concentrated *in vacuo*. Following flash column chromatography (99:1 pentane:diethyl ether), 1-benzyloxy-1-phenyl-3-butene was isolated as a colourless oil (0.604 g, 0.60 mmol, 21 %). δ_H (500 MHz, $CDCl_3$) 7.43- 7.29 (m, 10H), 5.87-5.78 (m, 1H), 5.10-5.02 (m, 2H), 4.50 (d, *J* = 11.9 Hz, 1H), 4.40 (t, 6.2 Hz, 1H), 4.31 (d, *J* = 11.9 Hz, 1H), 2.67 (ap quin, *J* = 6.8 Hz, 1H), 2.47 (ap quin, *J* = 7.1 Hz, 1H). δ_C (125 MHz, $CDCl_3$) 141.9, 138.5, 134.9, 128.4, 128.3, 127.7, 127.5, 126.9, 116.9, 81.2, 70.4, 42.7.

Data in accordance with that previously reported.[2]

1-(4-Methylphenyl)-4-penten-1-one (1l)

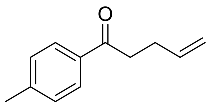

N,*N*-dimethylformamide (2 drops) was added dropwise to a rapidly stirred mixture of pentenoic acid (2.503 g, 25.00 mmol) and oxalyl chloride (3.427 g, 27.00 mmol). Once bubbling had ceased, further *N*,*N*-dimethylformamide (2 drops) was added, the reaction mixture was diluted with hexanes (10 mL), filtered through celite and concentrated *in vacuo*. The resulting colourless oil was diluted with anhydrous tetrahydrofuran (20 mL) and cooled to –78 °C, to this solution was added dropwise a mixture of *p*-tolylmagnesium bromide (1 M in tetrahydrofuran, 25 mL, 25 mmol) and iron(III) acetylacetonate (0.265 g, 0.75 mmol) in tetrahydrofuran (20 mL), under a nitrogen atmosphere, and also at –78 °C. The reaction mixture was stirred for 2 hours under a nitrogen atmosphere, then diluted

with HCl (1 M, 20 mL). The organic phase was extracted with diethyl ether (3 × 10 mL), washed with brine, dried over $MgSO_4$, and concentrated *in vacuo*. The product was isolated, following flash column chromatography (99:1 gradient to 9:1 petroleum spirit:ethyl acetate), as a pale yellow oil (0.881 g, 5.57 mmol, 23 %). δ_H (400 MHz, $CDCl_3$) 7.89 (d, J = 8.3 Hz, 2H), 7.28 (d, J = 8.0 Hz, 2H), 5.93 (ddt, J = 16.9 Hz, 10.2 Hz, 6.6 Hz, 1H), 5.14-5.01 (m, 2H), 3.07 (t, J = 7.3 Hz, 2H), 2.51 (m, 2H), 2.43 (s, 3H). δ_C (126 MHz, $CDCl_3$) 199.1, 143.8, 137.4, 134.5,129.3, 128.2, 115.2, 37.7, 28.3, 21.6.

Data in accordance with that previously reported.[3]

1-(4-Methylphenyl)-4-penten-1-ol (1m)

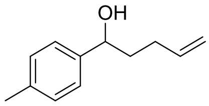

A solution of 1-(4-methylphenyl)-4-penten-1-one (0.871 g, 5.00 mmol) in ethanol (20 mL) was added to $NaBH_4$ (0.379 g, 10.00 mmol) and the resulting mixture was stirred under nitrogen for 18 hours. The reaction mixture was diluted with HCl (1 M, 20 mL). The organic phase was extracted with diethyl ether (3 × 10 mL), washed with brine, dried over $MgSO_4$, and concentrated *in vacuo*. The product was isolated, by flash column chromatography (8:2 petroleum spirit:diethyl ether), as a colourless oil (0.729 g, 4.14 mmol, 83 %). δ_H (500 MHz, $CDCl_3$) 7.27 (d, J = 8.1 Hz, 2H), 7.19 (d, J = 7.8 Hz, 2H), 5.87 (ddt, J = 17.1 Hz, 10.3 Hz, 3.6 Hz, 1H), 5.10-4.99 (m, 2H), 4.69 (m, 1H), 2.38 (s, 3H), 2.24-2.08 (m, 2H), 1.97-1.79 (m, 2H). δ_C (125 MHz, $CDCl_3$) 141.7, 138.3, 137.3, 129.2, 125.9, 114.9, 73.9, 38.0, 30.1, 21.1.

Preparation of Nitro- Derivatives

Methyl 4-nitrobenzoate (5q)

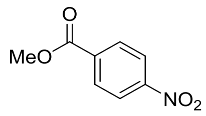

Sulfuric acid (10 drops) was added to a stirred solution of 4-nitrobenzoic acid (0.501 g, 3.00 mmol) in methanol (10 mL). The resulting mixture was stirred for 24 h under reflux. The reaction mixture was diluted with a saturated solution of $NaHCO_3$ (10 ml) and the organic layers were extracted with ethyl acetate (3 × 10 mL). The combined organic extracts were washed with brine (10 mL), dried with $MgSO_4$ and then the solvent removed *in vacuo* to give the ester (0.533 g, 2.94 mmol, 98 %). δ_H (500 MHz: $CDCl_3$) 8.30 (dt, *J* = 9.0 Hz, 2.1 Hz, 2H), 8.23 (dt, *J* = 9.1 Hz, 2.2 Hz, 2H), 3.99 (s, 3H); δ_C (126 MHz: $CDCl_3$) 165.2, 135.5, 130.7, 123.6, 52.8. IR υ_{max} (cm^{-1}): 1717, 1522, 1346, 1269, 1101.

Data were consistent with those of an authentic sample (Aldrich Cat. No. 274186).

Ethyl 4-nitrobenzoate (5r)

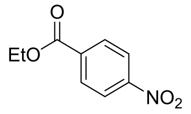

Sulfuric acid (10 drops) was added to a stirred solution of 4-nitrobenzoic acid (0.501 g, 3.00 mmol) in methanol (10 mL). The resulting mixture was stirred for 24 h under reflux. The reaction mixture was diluted with a saturated solution of $NaHCO_3$ (10 mL) was added and the organic layers were extracted with ethyl acetate (3 × 10 mL). The combined organic extracts were washed with brine (10 mL), dried with $MgSO_4$ and then evaporated under pressure to give the ester. The crude mixture was purified via silica column chromatography using a ratio of 6:4 petrol:ethyl acetate, yielding the pure ester (0.504 g, 2.56 mmol, 86 %). δ_H (600 MHz: $CDCl_3$) 8.29 (dt, *J* = 8.7 Hz, 1.9 Hz, 2H), 8.22 (dt, *J* = 9.1 Hz, 2.3 Hz, 2H), 4.45 (q, *J* = 7.2 Hz, 2H), 1.44 (t, *J* = 7.2 Hz, 3H). δ_C (150 MHz: $CDCl_3$) 164.7, 150.5, 135.9, 130.7, 123.5, 61.9. IR υ_{max} (cm^{-1}):1715, 1522, 1271, 1101.

Data was in accordance with that previously reported.[4]

4-(4-Nitrobenzoyl)morpholine (5s)

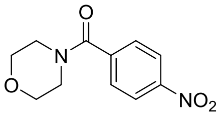

4-Nitrobenzoic acid (0.167 g, 1.00 mmol), one drop of dimethylformamide and an excess of oxalyl chloride (approx. 2.50 mL) were stirred at room temperature until no effervescence was apparent. Solvent was removed with flow of nitrogen and then 4-(dimethylamino)pyridine (0.025 g, 0.20 mmol), *N*,*N*-diisopropylethylamine (174 µL, 1.10 mmol), morpholine (87.0 µL, 1.00 mmol) and dichloromethane (2.0 mL) were added and stirred for 2 hours. Dichloromethane (10.0 mL) was added and washed with 4 M HCl (3 × 10 mL). The combined aqueous layers were neutralised using 6 M NaOH solution and washed with dichloromethane (3 × 10.0 mL). The combined organic extracts were washed with a sulfate buffer (1 × 10.0 mL), dried with $MgSO_4$ and then evaporated under pressure to give the amide. δ_H (600 MHz: $CDCl_3$) 8.26 (dt, *J* = 8.7 Hz, 1.9 Hz, 2H), 7.56 (dt, *J* = 8.7 Hz, 1.9 Hz),

3.78 (br s, 4H), 3.62 (br s, 2H), 3.37 (br s, 2H). IR υ_{max} (cm^{-1}):1624.0, 1514.1, 1440.8, 1350.2, 1282.7, 1132.2, 1107.1, 1008.8, 837.1.

Data was in accordance with that previously reported.[5]

1-(Methylsulfonyl)-4-nitro-benzene (5u)

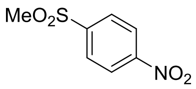

Dichloromethane (5 mL) was added to a mixture of potassium permanganate (1.00 g), manganese (II) sulphate monohydrate (1.00 g), and 4-nitrothioanisole (0.169 g, 1.00 mmol). The mixture was stirred for 72 h, then filtered and washed with dichloromethane (2 × 10 mL). The solvent was evaporated to yield the pure sulfone (yellow crystals, 0.1668 g, 83%). mp 138-141 °C (lit. 139-141 °C). δ_H (500 MHz: $CDCl_3$) 8.46-8.43 (m, 2H), 8.19-8.16 (m, 2H), 3.13 (s, 3H); δ_C (250 MHz: $CDCl_3$) 150.9, 146.0, 129.0, 124.6, 44.3; ν_{max} (solid state)/cm^{-1} 2924, 2853, 1678, 1522, 1337, 1321, 1290, 1151, 1086, 962, 853, 777, 737, 719, 681.

Data was in accordance with that previously reported.[6]

General Procedures for Reduction of Alkene Derivatives

All reactions were carried out at room temperature in a Radley's carousel multiple position reactor under an atmosphere of nitrogen.

General procedure **A** – Catalyst optimisation

A solution of 1-phenyl-4-butene (0.066 g, 0.50 mmol) or *trans*-*β*-methylstyrene (0.0661g, 0.50 mmol) in anhydrous ethanol (2 mL) was added to a mixture an iron salt (0.50 mmol) and sodium borohydride (0.038 g, 1.00 mmol). The resulting mixture was stirred under a nitrogen atmosphere for 18 h. Aqueous HCl (1 M, 10 mL) was added, and the aqueous phase extracted with diethyl ether (2 × 10 mL) and pentane (10 mL). The combined organic layers were then washed with brine, dried with $MgSO_4$ and concentrated *in vacuo*. 1,3,5-Trimethoxybenzene (0.017 g, 0.10 mmol) was added as internal standard, and the crude product mixture was analysed by ^1H NMR and GCMS.

General procedure **B** – Substrate screening

A solution of iron(III) triflate (0.050 g, 0.10 mmol) in anhydrous ethanol (2 mL) was added to a solution of a terminal alkene derivative (1.00 mmol) and sodium borohydride (0.057 g, 1.50 mmol) in anhydrous ethanol (2 mL). The resulting mixture was stirred under a nitrogen atmosphere for 6 h. The reaction mixture was diluted with a saturated solution of $NaHCO_3$ (10 mL) and the aqueous phase was extracted with dichloromethane (3 × 10 mL). The combined organic layers were then washed with brine, dried with $MgSO_4$ and concentrated in vacuo. 1,3,5-Trimethoxybenzene (0.017 g, 0.10 mmol) was added as internal standard, and the crude product mixture was analysed by ^1H NMR and GCMS.

In the case of products requiring further purification, the following procedure (**C**) was used to allow separation by flash column chromatography:[7]

The crude product mixture was diluted with dichloromethane (5 mL), *m*-chloroperbenzoic acid (0.248 g, 1.00 mmol) added, and the resulting mixture was stirred for 16 h. Sodium sulphite was added, and the reaction mixture was diluted with aqueous HCl (1 M, 10 mL) and the aqueous phase was extracted with dichloromethane (3 × 10 mL). The combined organic layers were then washed with brine, dried with $MgSO_4$ and concentrated *in vacuo*.

Table S1 Screen of activity of stoichiometric iron salts for the reduction of 4-phenyl-1-butene.[a]

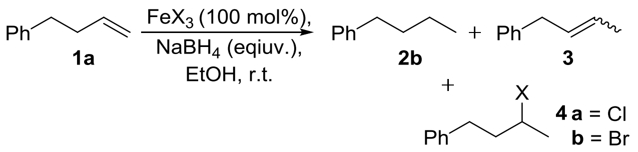

Entry	$FeX_{2/3}$	equiv. $NaBH_4$	yield (%)[b]		
			**2b**	**3**	**4**
1	$FeCl_3$	2	15	2	28
2	$FeCl_3$	3	50	3	25
3	$FeCl_3$	4	64	2	24
4	$FeBr_3$	2	42	1	14
5	$FeBr_3$	3	59	2	12
6	$Fe(OTf)_3$	1	19	3	0
7	$Fe(OTf)_3$	2	91	9	0
8	$Fe(OTf)_3$	3	93	7	0

[a] Conditions: 0.5 mmol 4-phenyl-1-butene, 0.5 mmol iron(III) salt, n equiv. $NaBH_4$, ethanol (2 ml), r.t., 16 h. [b] Yield measured by ^1H NMR spectroscopy of the crude reaction product using 1,3,5-trimethoxybenzene as internal standard.

Table S2 Screen of catalytic activity of simple iron salts for the reduction of 4-phenyl-1-butene.[a]

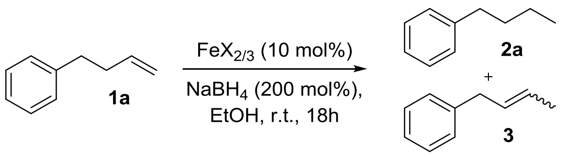

Entry	Iron Salt	Conversion (%)[b] **2a**	**3**
1	FeF_3	0	0
2	$FeF_3{\cdot}3H_2O$	3	0
3	$FeCl_3$	91	5
4	$FeCl_3$ (>99.99 %)	89	6
5	$FeBr_3$	90	9
6	$Fe(NO_3)_3{\cdot}9H_2O$	17	0
7	$Fe(acac)_3$	0	0
8	Fe_2O_3	29	0
9	$Fe(OTf)_3$	90	10
10[c]	$Fe(OTf)_3$	9	0
11[d]	$Fe(OTf)_3$	6	3
12[e]	$Fe(OTf)_3$	78	13
13[f]	$Fe(OTf)_3$	19	0
14	$Fe_2(SO_4)_3{\cdot}6H_2O$	0	0
15	$FePO_4{\cdot}4H_2O$	0	0
16	Fe(OH)O	0	0
17	$Fe^{III}(OTs)_3{\cdot}6H_2O$	75	8
18	$K_3Fe(CN)_6$	6	0
19	FeF_2	3	0
20	$FeF_2{\cdot}4H_2O$	5	0
21	$FeCl_2$	81	14
22	$FeBr_2$	58	8
23	FeI_2	54	20
24	$Fe(OTf)_2$	11	0
25	$Fe(BF_4)_2{\cdot}6H_2O$	41	0
26	$Fe(acac)_2$	3	0
27	$Fe(OAc)_2$	3	0
28	$Fe(SO_4)$	5	0
29	Fe^{II} citrate	0	0
30	$Fe^{II}(C_2O_4){\cdot}2H_2O$	5	0
31	$Fe^{II}(MoO_4)$	3	0
32	Fe^{II} gluconate hydrate	3	0
33	$K_4Fe(CN)_6$	6	0
34	$Cp^*_2Fe_2(CO)_4$	0	0
35	Ferrocene	10	0
36	Fe_2CO_9	0	0

[a] Conditions: According to general procedure **A**; 0.50 mmol 4-phenyl-1-butene, 10 mol% FeX_n, 200 mol% $NaBH_4$, ethanol (2 ml), r.t., 18 h. [b] Conversion measured by ^1H NMR spectroscopy. [c] Reaction performed in methanol (2 mL). [d] Reaction performed in acetonitrile (2 mL). [e] Reaction performed in 1-butanol (2 mL). [f] Reaction performed in 2-butanol (2 mL).

Table S3 **C**atalytic activity of simple iron salts for the reduction of *trans*-β-methylstyrene.[a]

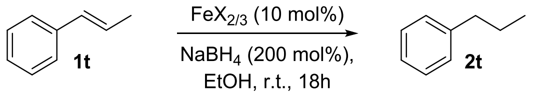

Entry	Iron Salt	Conversion (%)[b] **2t**
1	$FeF_3 \cdot 3H_2O$	0
2	$FeCl_3$	2
3	$FeBr_3$	2
4	$Fe(NO_3)_3 \cdot 9H_2O$	2
5	$Fe(acac)_3$	0
6	Fe_2O_3	0
7	$Fe(OTf)_3$	1
8	$Fe_2(SO_4)_3 \cdot 6H_2O$	0
9	$FePO_4 \cdot 4H_2O$	0
10	Fe(OH)O	0
11	$Fe^{III}(OTs)_3 \cdot 6H_2O$	1
12	$K_3Fe(CN)_6$	0
13	FeF_2	1
14	$FeF_2 \cdot 4H_2O$	0
15	$FeCl_2$	1
16	$FeBr_2$	1
17	FeI_2	1
18	$Fe(OTf)_2$	1
19	$Fe(BF_4)_2 \cdot 6H_2O$	1
20	$Fe(acac)_2$	1
21	$Fe(OAc)_2$	0
22	$Fe(SO_4)$	1
23	Fe^{II} citrate	0
24	$Fe^{II}(C_2O_4) \cdot 2H_2O$	3
25	Fe^{II} (MoO_4)	2
26	$K_4Fe(CN)_6$	0
27	Ferrocene	0
28	Fe_2CO_9	0

[a] Conditions: According to general procedure **A**; 0.50 mmol *trans*-β-methylstyrene, 10 mol% FeX_n, 200 mol% $NaBH_4$, ethanol (2 ml), r.t., 18 h.[b] Conversion measured by ^1H NMR spectroscopy.

ICP-MS Analysis

$Fe(OTf)_3$ (0.3 mg) was dissolved in HNO_3 (5 mL, 2 % w/w).

ICP-MS (Inductively Coupled Plasma Mass Spectroscopy) analysis was performed on $Fe(OTf)_3$ (0.3 mg) .

Analysis was performed on an Agilent 7500ce inductively coupled plasma mass spectrometer calibrated against calibrated multi-element standard solution.

The abundance of each metal was recorded relative to the abundance of iron.

Table S4 ICP-MS analysis of the relative abundance (ppm) of metals in $Fe(OTf)_3$

Al	Cr	Mn	Ni	Cu	Mo	Ru	Rh	Pd	Ag	Ir	Pt
18120	110	749	27	89	19	<1	<1	1	36	<1	<1

In order to investigate whether trace metal impurities present in the iron salts were involved in the observed catalysis, a series of control reactions were performed.[8]

According to general procedure **A**, 4-phenyl-1-butene (0.066 g, 0.50 mmol), sodium borohydride (0.027 g, 0.75 mmol) and either aluminium trichloride (0.007 g, 0.05 mmol), silver(I) triflate (0.013 g, 0.05 mmol), copper(II) chloride (0.007 g, 0.05 mmol), manganese(II) chloride (0.006 g, 0.05 mmol) or nickel(II) chloride (0.007 g, 0.05 mmol) were reacted in anhydrous ethanol (2 mL).

Nickel and copper salts have both been previously shown to be active for the hydrogenation of alkenes using strong hydride sources.[9] Salts of both metals were capable of reducing 4-phenyl-1-butene to phenylbutane at 10 mol% (table S4, entries 3 and 6) however, at the ppm measured by ICP-MS analysis, neither were effective catalysts (entries 4 and 7).

Table S5 Investigation into the role of metal impurities in $Fe(OTf)_3$[a]

Entry	Metal Salt	Loading	Yield of phenylbutane (%)[b]
1	$AlCl_3$	10 mol %	4
2	AgOTf	10 mol %	2
3	$CuCl_2$	10 mol %	54
4	$CuCl_2$	90 ppm	0
5	$MnCl_2$	10 mol %	11
6	$NiCl_2$	10 mol %	80
7	$NiCl_2$	30 ppm	0

[a] Conditions: According to general procedure **A**; 0.50 mmol 4-phenyl-1-butene, 10 mol% metal salt, 150 mol% $NaBH_4$, EtOH (2 ml), r.t., 6 h.[b] Yield measured by ^1H NMR spectroscopy relative to 1,3,5-trimethoxybenzene internal standard.

Additionally, according to general procedure **B**, 4-*tert*-butylstyrene (0.166 g, 1.00 mmol), sodium borohydride (0.057 g, 1.50 mmol) and iron(III) triflate (0.050 g, 0.10 mmol) and either $CuCl_2$ (90 ppm) or $NiCl_2$ (30 ppm) were reacted in anhydrous ethanol (4 mL). With additional copper and nickel, the yield of ethyl-4-*tert*-butylbenzene (by ^1H NMR spectroscopy) was 55% and 45% respectively, showing no improvement over the standard reaction (table 3, entry 15).

Screening of Alkene Substrates (Table 2)

Table 2, entry 1

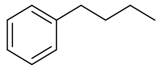

Butylbenzene (2a)
According to general procedure **B**, 4-phenyl-1-butene (0.132 g, 1.00 mmol), sodium borohydride (0.057 g, 1.50 mmol) and iron(III) triflate (0.050 g, 0.10 mmol) were reacted in anhydrous ethanol (4 mL). The resulting oil was reacted with *m*-chloroperbenzoic acid (0.248 g, 1.00 mmol) in dichloromethane (5 mL) according to general procedure **C**. Following flash column chromatography (99:1 pentane:diethyl ether) butylbenzene (0.11 g, 0.83 mmol, 83%) was obtained as a colourless oil. δ_H (500 MHz, $CDCl_3$) 7.31 (t, *J* = 8.0 Hz, 2H), 7.23-7.18 (m, 3H), 2.65 (t, *J* = 7.8 Hz, 2H), 1.68-1.60 (m, 2H), 1.40 (ap sex, *J* = 7.6 Hz, 2H), 0.96 (t, *J* = 1.0 Hz, 3H). δ_C (125 MHz, $CDCl_3$) 143.0, 128.5, 128.3, 125.6, 35.7, 33.7, 22.4, 14.0.

Data in accordance with that previously reported.[10]

Table 2, entry 2

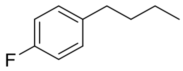

1-Fluoro-4-butylbenzene (2b)
According to general procedure **B**, 1-fluoro-4-butenylbenzene (0.150g, 1.00 mmol), sodium borohydride (0.057 g, 1.50 mmol) and iron(III) triflate (0.050 g, 0.10 mmol) were reacted in anhydrous ethanol (4 mL). The resulting oil was reacted with *m*-chloroperbenzoic acid (0.248 g, 1.00 mmol) in dichloromethane (5 mL) according to general procedure **C**. Following flash column chromatography (99:1 pentane:diethyl ether) 1-fluoro-4-butylbenzene (0.120 g, 0.79 mmol, 79%) was obtained as a colourless oil. δ_H (500 MHz, $CDCl_3$) 7.17-7.12 (m, 2H), 6.98 (tt, *J* = 8.8 Hz, 2.1 Hz, 2H), 2.61 (t, *J* = 7.9 Hz, 2H), 1.64-1.57 (m, 2H), 1.41-1.33 (m, 2H), 0.96 (t, *J* = 7.4 Hz, 3H). δ_C (125 MHz, $CDCl_3$) 161.1 (d, *J* = 242.8 Hz), 138.5 (d, *J* = 3.5 Hz), 129.7 (d, *J* = 8.0 Hz), 114.9 (d, *J* = 20.9 Hz), 34.8, 33.8, 22.3, 13.9.

Data in accordance with that previously reported.[11]

Table 2, entry 3

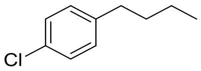

1-Chloro-4-butylbenzene (2c)
According to general procedure **B**, 1-chloro-4-butenylbenzene (0.166 g, 1.00 mmol), sodium borohydride (0.057 g, 1.50 mmol) and iron(III) triflate (0.050 g, 0.10 mmol) were reacted in anhydrous ethanol (4 mL). The resulting oil was reacted with *m*-chloroperbenzoic acid (0.248 g, 1.00 mmol) in dichloromethane (5 mL) according to general procedure **C**. Following flash column chromatography (99:1 pentane:diethyl ether) 1-chloro-4-butylbenzene (0.129 g, 0.79 mmol, 77%) was obtained as a colourless oil. δ_H (500 MHz, $CDCl_3$) 7.26 (d, *J* = 8.4 Hz, 2H), 7.13 (d, *J* = 8.4 Hz, 2H), 2.60 (t, *J* = 7.8 Hz, 2H), 1.64-1.57 (m, 2H), 1.37 (app sex, *J* = 7.5 Hz, 2H), 0.95 (t, *J* = 7.4 Hz, 3H). δ_C (125 MHz, $CDCl_3$) 141.3, 131.3, 129.8, 128.3, 35.0, 33.6, 22.3, 13.9.

Data in accordance with that previously reported.[12]

Table 2, entry 4

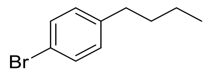

1-Bromo-4-butylbenzene (2d)
According to general procedure **B**, 1-bromo-4-butenylbenzene (0.210 g, 1.00 mmol), sodium borohydride (0.057 g, 1.50 mmol) and iron(III) triflate (0.050 g, 0.10 mmol) were reacted in anhydrous ethanol (4 mL). The resulting oil was reacted with *m*-chloroperbenzoic acid (0.248 g, 1.00 mmol) in dichloromethane (5 mL) according to general procedure **C**. Following flash column chromatography (99:1 pentane:diethyl ether) 1-bromo-4-butylbenzene (0.150 g, 0.71 mmol, 71%) was obtained as a colourless oil. δ_H (500 MHz, $CDCl_3$) 7.42 (dt, J = 8.5 Hz, 2.1 Hz, 2H), 7.08 (dt, J = 8.1 Hz, 2.1 Hz, 2H), 2.59 (t, J = 7.8 Hz, 2H), 1.64-1.57 (m, 2H), 1.37 (app sex, J = 7.7 Hz, 2H), 0.95 (t, J = 7.4 Hz, 3H). δ_C (125 MHz, $CDCl_3$) 141.8, 131.3, 130.2, 119.3, 35.0, 33.5, 22.2, 13.9.

Data in accordance with that previously reported.[13]

Table 2, entry 5

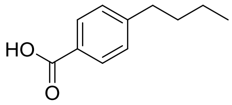

4-Butyl-benzoic acid (2e)
According to general procedure **B**, 4-(3-butenyl)-benzoic acid (0.176 g, 1.00 mmol), sodium borohydride (0.056 g, 1.50 mmol) and iron(III) triflate (0.050 g, 0.10 mmol) were reacted in anhydrous ethanol (4 mL). Product was not isolated.

Data in accordance with that previously reported.[14]

Table 2, entry 6

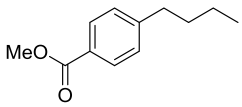

4-Butyl-benzoic acid, methyl ester (2f)
According to general procedure **B**, 4-(3-butenyl)-benzoic acid, methyl ester (0.190 g, 1.00 mmol), sodium borohydride (0.057 g, 1.50 mmol) and iron(III) triflate (0.050 g, 0.10 mmol) were reacted in anhydrous ethanol (4 mL). The resulting oil was reacted with *m*-chloroperbenzoic acid (0.248 g, 1.00 mmol) in dichloromethane (5 mL) according to general procedure **C**. Following flash column chromatography (9:1 pentane:diethyl ether) 4-butyl-benzoic acid, methyl ester (0.181 g, 0.94 mmol, 94 %) was obtained as a colourless oil. δ_H (400 MHz, $CDCl_3$) 7.97 (dt, J = 8.4 Hz, 1.9 Hz, 2H), 7.26 (d, J = 8.1 Hz, 2H), 3.92 (s, 3H), 2.69 (t, J = 7.8 Hz, 2H), 1.68-1.59 (m, 2H), 1.43-1.35 (m, 2H), 0.95 (t, 7.4 Hz, 3H). δ_C (100 MHz, $CDCl_3$) 148.5, 129.6, 128.4, 127.6, 51.9, 35.7, 33.3, 22.3, 13.9.

Data in accordance with that previously reported.[15]

Table 2, entry 8

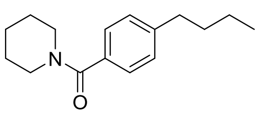

1-(4-Butylbenzoyl)-piperidine (2h)
According to general procedure **B**, 1-bromo-4-butenylbenzene (0.243g, 1.00 mmol), sodium borohydride (0.057 g, 1.50 mmol) and iron(III) triflate (0.050 g, 0.10 mmol) were reacted in anhydrous ethanol (4 mL). The resulting oil was reacted with *m*-chloroperbenzoic acid (0.248 g, 1.00

mmol) in dichloromethane (5 mL) according to general procedure **C**. Following flash column chromatography (70:30 petroleum spirit:diethyl ether) 1-(4-butylbenzoyl)-piperidine (0.202 g, 0.87 mmol, 87%) was obtained as a colourless oil. δ_H (500 MHz, $CDCl_3$) 7.32 (d, *J* = 8.1 Hz, 2H), 7.21 (d, *J* = 8.2 Hz, 2H), 3.72 (br s, 2H), 3.39 (br s, 2H), 2.64 (t, *J* = 7.8 Hz, 2H), 1.76 – 1.46 (br m, 8H), 1.38 (ap sex, *J* = 7.6 Hz, 2H), 0.95 (t, *J* = 7.3 Hz, 3H). δ_C (125 MHz, $CDCl_3$) 170.6, 144.4, 133.8, 128.4, 126.9, 48.8 (br), 43.2 (br), 35.5, 33.5, 26.6 (br), 25.7 (br), 24.7, 22.3, 13.9. HRMS (EI) Exact mass cald. for $C_{16}H_{23}NO$ $[M]^+$: 245.1780, found: 245.1774.

Table 2, entry 9

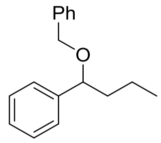

1-Benzyloxy-1-phenylbutane (2i)
According to general procedure **B**, 1-benzyloxy-1-phenyl-3-butene (0.238g, 1.00 mmol), sodium borohydride (0.057 g, 1.50 mmol) and iron(III) triflate (0.050 g, 0.50 mmol) were reacted in anhydrous ethanol (4 mL). The resulting oil was reacted with *m*-chloroperbenzoic acid (0.248 g, 1.00 mmol) in dichloromethane (5 mL) according to general procedure **C**. Following flash column chromatography (99:1 pentane:diethyl ether) 1-benzyloxy-1-phenyl-3-butane (0.120 g, 0.50 mmol, 50%) was obtained as a colourless oil. δ_H (600 MHz, $CDCl_3$) 7.41-7.29 (m, 10H), 4.48 (d, *J* = 12.1 Hz, 1H), 4.34 (dd, *J* = 7.6 Hz, 5.7 Hz, 1H), 4.28 (d, *J* = 11.7 Hz, 1H), 1.93-1.85 (m, 1H), 1.69-1.62 (m, 1H), 1.53-1.45 (m, 1H), 1.36-1.29 (m, 1H), 0.91 (t, *J* = 7.6 Hz, 1H). δ_C (150 MHz, $CDCl_3$) 145.47, 141.41, 131.06, 130.99, 130.43, 130.14, 130.11, 129.50, 84.00, 73.07, 43.23, 21.82, 16.64.

Data in accordance with that previously reported.[16]

Table 2, entry 11

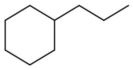

Propylcyclohexane (2k)
According to general procedure **B**, allylcyclohexane (0.124 g, 1.00 mmol), sodium borohydride (0.057 g, 1.50 mmol) and iron(III) triflate (0.050 g, 0.10 mmol) were reacted in anhydrous ethanol (4 mL). Propylcyclohexane was obtained as a colourless oil (0.086 g, 0.69 mmol, 69%). δ_H (500 MHz, $CDCl_3$) 1.76-1.63 (m), 1.38-1.14 (m), 0.95-0.86 (m). δ_C (126 MHz, $CDCl_3$) 39.9, 37.4, 33.5, 26.8, 26.5, 22.4, 19.9, 14.4, 14.0.

Data were consistent with those of an authentic sample (Aldrich Cat. No. 111856).

Table 2, entry 14

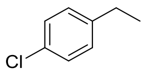

1-Ethyl-4-chlorobenzene (2n)
According to general procedure **B**, 4-chlorostyrene (0.139 g, 1.00 mmol), sodium borohydride (0.057 g, 1.50 mmol) and iron(III) triflate (0.050 g, 0.10 mmol) were reacted in anhydrous ethanol (4 mL). The resulting oil was reacted with *m*-chloroperbenzoic acid (0.248 g, 1.00 mmol) in dichloromethane (5 mL) according to general procedure **C**. Following flash column chromatography (99:1 pentane:diethyl ether) 1-chloro-4-ethylbenzene (0.016 g, 0.12 mmol, 12%) was obtained as a colourless oil. δ_H (600 MHz, $CDCl_3$) 7.25 (dt, J = 8.3 Hz, 1.9 Hz, 2H), 7.13 (m, 2H), 2.63 (q, J = 7.6 Hz, 2H), 1.23 (t, J = 7.9 Hz, 3H). δ_C (150 MHz, $CDCl_3$) 145.3, 133.9, 131.9, 131.0, 28.3, 15.5.

Data in accordance with that previously reported.[10]

Table 2, entry 15

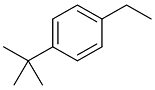

1-Ethyl-4-*tert*-butylbenzene (2o)
According to general procedure **B**, 4-*tert*-butylstyrene (0.166 g, 1.00 mmol), sodium borohydride (0.057 g, 1.50 mmol) and iron(III) triflate (0.050 g, 0.10 mmol) were reacted in anhydrous ethanol (4 mL). The resulting oil was reacted with *m*-chloroperbenzoic acid (0.248 g, 1.00 mmol) in dichloromethane (5 mL) according to general procedure **C**. Following flash column chromatography (95:5 pentane:diethyl ether) 1-ethyl-4-*tert*-butylbenzene (0.073 g, 0.45 mmol, 45%) was obtained as a colourless oil. δ_H (500 MHz, $CDCl_3$) 7.37 (d, J = 8.3 Hz, 2H), 7.20 (d, J = 8.4 Hz, 2H), 2.68 (q, J = 7.7 Hz, 2H), 1.37 (s, 3H), 1.29 (t, J = 7.7 Hz, 3H). δ_C (126 MHz, $CDCl_3$) 148.4, 141.2, 127.5, 125.2, 34.4, 31.5, 28.3, 15.5.

Data in accordance with that previously reported.[10]

Table 2, entry 16

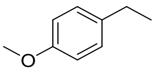

1-Ethyl-4-methoxybenzene (2p)
According to general procedure **B**, 4-methoxystyrene (0.134 g, 1.00 mmol), sodium borohydride (0.057 g, 1.50 mmol) and iron(III) triflate (0.050 g, 0.10 mmol) were reacted in anhydrous ethanol (4 mL). The resulting oil was reacted with *m*-chloroperbenzoic acid (0.248 g, 1.00 mmol) in dichloromethane (5 mL) according to general procedure **C**. Following flash column chromatography (9:1 pentane:diethyl ether) 1-ethyl-4-methoxybenzene (0.067 g, 0.49 mmol, 49%) was obtained as a colourless oil. δ_H (500 MHz, $CDCl_3$) 7.16 (dt, J = 8.8 Hz, 2.1 Hz, 2H), 6.88 (dt, J = 8.7 Hz, 2.13 Hz, 2H), 3.83 (s, 3H), 2.64 (q, J = 7.7 Hz, 2H), 1.26 (t, J = 7.7 Hz, 3H). δ_C (126 MHz, $CDCl_3$) 157.7, 136.4, 128.7, 113.7, 55.3, 28.0, 16.0.

Data in accordance with that previously reported.[10]

Table 2, entry 17

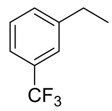

1-Ethyl-3-(trifluoromethyl)benzene (2q)
According to general procedure **B**, 4-methoxystyrene (0.134 g, 1.00 mmol), sodium borohydride (0.057 g, 1.50 mmol) and iron(III) triflate (0.050 g, 0.10 mmol) were reacted in anhydrous ethanol (4 mL). Product was not isolated.

Table 2, entry 18

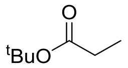

***tert*-Butyl propionate (2r)**
According to general procedure **B**, *tert*-butylacetate (0.128 g, 1.00 mmol), sodium borohydride (0.057 g, 1.50 mmol) and iron(III) triflate (0.050 g, 0.10 mmol) were reacted in anhydrous ethanol (4 mL). *tert*-butyl propionate was obtained as a colourless oil (0.096 g, 0.74 mmol, 74 %). δ_H (500 MHz, $CDCl_3$) 2.25 (q, J = 7.5 Hz, 2H), 1.46 (s, 9H), 1.11 (t, J = 7.6 Hz, 3H). δ_C (126 MHz, $CDCl_3$) 174.0, 79.9, 28.8, 28.1, 9.2.

Data were consistent with those of an authentic sample (Aldrich Cat. No. 254525).

Table 2, entry 19

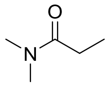

***N*,*N*-Dimethylpropionamide (2s)**
According to general procedure **B**, *N*,*N*-dimethylacrylamide (0.099 g, 1.00 mmol), sodium borohydride (0.057 g, 1.50 mmol) and iron(III) triflate (0.050 g, 0.10 mmol) were reacted in anhydrous ethanol (4 mL). Following flash column chromatography (8:2, pentane:diethyl ether) an inseparable mixture of the *N*,*N*-dimethylpropionamide and starting material, *N*,*N*-dimethylacrylamide, was obtained as a colourless oil.

Data were consistent with those of an authentic sample (Aldrich Cat. No. 252875).

General Procedures of the Reduction of Nitro- Groups

General procedure **D**:

Anhydrous ethanol (4 mL) was added to a mixture of sodium borohydride (0.379 g, 10.00 mmol), iron(III) triflate (0.025 g, 0.05 mmol) and a nitroarene (0.50 mmol) and the resulting mixture was stirred at room temperature under a nitrogen atmosphere. After 4 hours the reaction mixture was diluted with a saturated solution of $NaHCO_3$ (10 mL) and the aqueous phase extracted with dichloromethane (3 x 10 mL). The combined organic extracts were washed with brine (20 mL), dried over $MgSO_4$ and concentrated *in vacuo*. 1,3,5-Trimethoxybenzene (0.0168 g, 0.10 mmol) was added as an internal standard, and the crude reaction mixture was analysed by ^{1}H NMR spectroscopy.

Products were isolated either by flash column chromatography or one of the following methods:

General procedure **E** (acid/base extraction):

The crude reaction mixture was diluted with dichloromethane (10 mL), extracted three times with aqueous hydrochloric acid (4 M, 10 mL), the aqueous phase was treated with aqueous sodium hydroxide (6 M) until basic. The aqueous phase was then extracted with dichloromethane (3 x 10 mL), and the combined organic extracts washed with brine (20 mL), dried over $MgSO_4$, and concentrated *in vacuo* to give the isolated arylamine.

General procedure **F** (isolation as hydrochloride salt):

The crude reaction mixture was diluted with diethyl ether (2 mL) and hydrochloric acid (2 M in diethyl ether, 0.25 mL, 0.5 mmol) was added. The mixture was filtered to give the isolated arylamine as a salt with HCl.

Table S6 Catalytic activity of simple iron salts and Lewis acids for the reduction of nitrobenzene.[a]

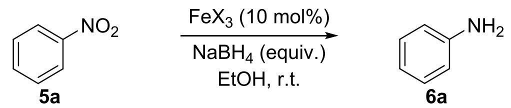

Entry	$FeX_{2/3}$	$NaBH_4$ equiv.	t (h)	Conversion (%)[b]
1	$FeCl_3$	2	16	15
2	$FeCl_3$	4	16	51
3	$FeCl_3$	20	16	88
4	$FeCl_3$ (99.99%)	20	16	90
5	$FeCl_2$	20	16	62
6	$Fe(OTf)_2$	20	16	60
7	$Fe(OTf)_3$	20	16	99
8	$Fe(OTf)_3$	10	16	32
9	$Fe(OTf)_3$	20	4	99
10	$BF_3 \cdot Et_2O$	20	4	0
11	$AlCl_3$	20	4	0
12	HOTf	20	4	1

[a] Conditions: 0.50 mmol nitrobenzene, 10 mol% iron salt, $NaBH_4$, EtOH (4 ml), r.t., 18 h. [b] Conversion measured by ^1H NMR spectroscopy.

Screening of Nitro- Functionalised Substrates (Table 3)

Table 4, entry 1

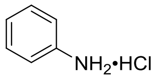

Aniline hydrochloride (6a)

According to general procedure **D**, nitrobenzene (0.062 g, 0.052 mL, 0.50 mmol), iron(III) triflate (0.025 g, 0.05 mmol) and sodium borohydride (0.378 g, 10 mmol) were reacted in anhydrous ethanol (4 mL). Crude product was obtained as a yellow oil (90 %). Following purification by general procedure **F**, aniline hydrochloride was isolated as an amorphous grey powder (0.052 g, 0.40 mmol, 80%), mp 199 °C (from diethyl ether). δ_H (500 MHz, CD_3OD) 7.55 (t, *J* = 8.4 Hz, 2H), 7.50 (t, *J* = 7.4 Hz, 1H), 7.41 (d, *J* = 7.0 Hz, 2H). δ_C (500 MHz, CD_3OD) 132.1, 131.5, 130.5, 124.2. IR υ_{max} (cm^{-1}): 3360, 2808.36, 2594.26, 2358.94, 2011.76, 1600.92, 1490.97, 738.74, 684.73.

Data was in accordance with that previously reported.[17]

Table 4, entry 2

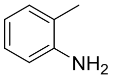

2-Aminotoluene (6b)

Anhydrous ethanol (4 mL) was added to a mixture of sodium borohydride (0.379 g, 10.00 mmol), iron(III) triflate (0.025 g, 0.05 mmol) and 2-nitrotoluene (0.069 g, 0.059 mL, 0.50 mmol) and the resulting mixture was stirred at room temperature under a nitrogen atmosphere. After 4 hours the reaction mixture was diluted with a saturated solution of $NaHCO_3$ (10 mL) and the aqueous phase extracted with dichloromethane (3 x 10 mL). The combined organic extracts were washed with brine (20 mL), dried over $MgSO_4$ and concentrated *in vacuo*. 1,2-Dichloroethane (0.049 g, 0.039 mL, 0.10 mmol) was added as an internal standard, and the crude reaction mixture was analysed by ^{1}H NMR spectroscopy. Crude product was obtained (80 %). Following removal of the internal standard *in vacuo*, 2-aminotoluene was isolated as a dark brown oil (0.043 g, 0.40 mmol, 80 %). δ_H (400 MHz, $CDCl_3$) 7.11-7.04 (br m, 2H), 6.74 (t, *J* = 7.5 Hz, 1H), 6.71 (d, *J* = 7.8 Hz, 1H), 3.30 (br s, 2H), 2.21 (s, 3H). δ_C (100 MHz, $CDCl_3$) 144.5, 130.5, 127.0, 122.4, 118.7, 115.0, 17.4.

Data were consistent with those of an authentic sample (Aldrich Cat. No. 185426).

Table 4, entry 3

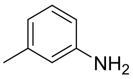

3-Aminotoluene (6c)

According to general procedure **D**, 3-nitrotoluene (0.069 g, 0.059 mL, 0.50 mmol), iron(III) triflate (0.025 g, 0.05 mmol) and sodium borohydride (0.378 g, 10.00 mmol) were reacted in anhydrous ethanol (4 mL). Crude product was obtained (61%). Following purification by general procedure **E**, 3-aminotoluene was isolated as a light brown oil (0.026 g, 0.25 mmol, 49%). δ_H (400 MHz, $CDCl_3$) 7.05 (t, *J* = 7.7 Hz, 1H), 6.59 (d, *J* = 7.5 Hz, 1H), 6.55-6.49 (br m, 2H), 3.60 (br s, 2H), 2.28 (s, 3H). δ_C (100 MHz, $CDCl_3$) 146.3, 139.1, 129.1, 119.4, 115.9, 112.2, 21.4.

Data were consistent with those of an authentic sample (Aldrich Cat. No. 511218).

Table 4, entry 4

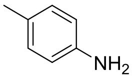

4-Aminotoluene (6d)

According to general procedure **D**, 4-nitrotoluene (0.069 g, 0.05 mL, 0.50 mmol), iron(III) triflate (0.025 g, 0.05 mmol) and sodium borohydride (0.378 g, 10.00 mmol) were reacted in anhydrous ethanol (4 mL). Crude product was obtained (73%). Following purification by general procedure **E**, 4-aminotoluene was isolated (0.035 g, 0.33 mmol, 66%). δ_H (400 MHz, $CDCl_3$) 7.00 (d, *J* = 8.1, 2H), 6.65 (d, *J* = 8.3 Hz, 2H), 3.25(br.s, 2H), 2.20 (s, 2H). δ_C (100 MHz, $CDCl_3$) 129.74, 127.88, 115.52, 20.42.

Data were consistent with those of an authentic sample (Aldrich Cat. No. 236314).

Table 4, entry 5

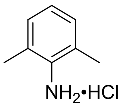

2,6-Dimethylaniline hydrochloride (6e)

According to general procedure **D**, 1,3-dimethyl-2-nitrobenzene (0.076 g, 0.068 mL, 0.50 mmol), iron(III) triflate (0.025 g, 0.05 mmol) and sodium borohydride (0.378 g, 10.00 mmol) were reacted in anhydrous ethanol (4 mL). Crude product was obtained (79 %). Following purification by general procedure **F**, 2,6-dimethylaniline hydrochloride was isolated (0.047 g, 0.30 mmol, 59%). δ_H (400 MHz, CD_3OD) 7.30-7.26 (m, 1H), 7.24-7.21 (m, 2H), 2.44 (s, 6H). δ_C (100 MHz, CD_3OD) 131.42, 129.29, 128.53, 128.04, 16.23. IR υ_{max} (cm^{-1}): 2816, 2639, 2361, 2012, 1584, 1530, 1464, 1179, 762, 687.

Data was in accordance with that previously reported.[18]

Table 4, entry 6

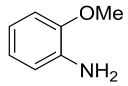

2-Methoxyaniline (6f)

According to general procedure **D**, 2-nitroanisole (0.077 g, 0.061 mL, 0.50 mmol), iron(III) triflate (0.0252 g, 0.05 mmol) and sodium borohydride (0.378 g, 10.00 mmol) were reacted in anhydrous ethanol (4 mL). Crude product was obtained (76 %). Following purification by general procedure **E**, 2-methoxyaniline was isolated as a colourless oil (0.046 g, 0.37 mmol, 75 %). δ_H (400 MHz, $CDCl_3$) 6.89-6.75 (m, 4H), 3.90 (s, 3H), 3.74 (br s, 2H). δ_C (100 MHz, $CDCl_3$) 147.2, 136.1, 121.0, 118.4, 114.9, 110.3, 55.3.

Data was in accordance with that previously reported.[17]

Table 4, entry 7

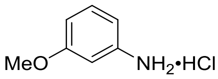

3-Methoxyaniline hydrochloride (6g)

According to general procedure **D**, 3-nitroanisole (0.077 g, 0.50 mmol), iron(III) triflate (0.025 g, 0.05 mmol) and sodium borohydride (0.378 g, 10.00 mmol) were reacted in anhydrous ethanol (4 mL). Crude product was obtained (81 %). Following purification by general procedure **F**, 3-methoxyaniline hydrochloride was isolated as an amorphous brown powder (0.054 g,0.34 mmol, 68%), mp: 167-168 °C. δ_H (400 MHz, CD_3OD) 7.45 (m, 1H), 7.06 (dd, J = 8.4 Hz, 2.4 Hz, 1H), 6.95 (dd, J = 7.8 Hz, 2.1 Hz, 1H), 6.93 (m, 1H), 3.85 (s, 3H). δ_C (100 MHz, CD_3OD) 162.5, 133.1, 132.4, 116.1, 115.7, 110.3, 56.3. IR υ_{max} (cm^{-1}): 2940, 2600, 2359, 1578, 1539, 1466, 1423, 1319, 1252, 1159, 1038.

Data was in accordance with that previously reported.[18]

Table 4, entry 8

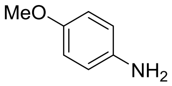

4-Methoxyaniline (6h)

According to general procedure **D**, 4-nitroanisole (0.077 g, 0.50 mmol), iron(III) triflate (0.025 g, 0.05 mmol) and sodium borohydride (0.378 g, 10.00 mmol) were reacted in anhydrous ethanol (4 mL). Crude product was obtained (68 %). Following purification by general procedure **E**, 4-methoxyaniline was isolated as a colourless oil (0.015 g, 0.12 mmol, 24 %). δ_H (400 MHz, $CDCl_3$) 6.76 (dt, J = 9.0 Hz, 2.3 Hz, 2H), 6.66 (d, J = 9.0 Hz, 2.4 Hz, 2H), 3.76 (s, 3H), 3.32 (br s, 2H). δ_C (100 MHz, $CDCl_3$) 152.7, 139.9, 116.4, 114.8, 55.9.

Data was in accordance with that previously reported.[18]

Table 4, entry 9

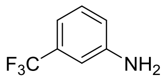

3-(Trifluoromethyl)aniline (6i)

According to general procedure **D**, 3-nitrobenzotrifluoride (0.067 mL, 0.50 mmol), iron(III) triflate (0.025 g, 0.05 mmol) and sodium borohydride (0.378 g, 10 mmol) were reacted in anhydrous ethanol (4 mL). Crude product was obtained (82 %). Following purification by general procedure **E**, 3-(trifluoromethyl) aniline was isolated as a yellow oil (0.044 g, 0.27 mmol, 55 %). δ_H (500 MHz, $CDCl_3$) 7.27 (t, J = 7.8 Hz, 1H), 7.02 (dt, J = 7.7 Hz, 0.7 Hz, 1H), 6.92 (s, 1H), 6.84 (dd, J = 8.0 Hz, 2.3 Hz, 1H), 3.86 (br s, 2H). δ_C (126 MHz, $CDCl_3$) 146.7, 131.6 (q, J = 31.9 Hz), 129.7, 124.2 (q, J = 272.0 Hz), 118.0, 115.0 (q, J = 4.0 Hz), 111.3 (q, J = 4.0 Hz).

Data was in accordance with that previously reported.[19]

Table 4, entry 10

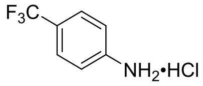

4-(Trifluoromethyl)aniline hydrochloride (6j)

According to general procedure **D**, 4-nitrobenzotrifluoride (0.096 g, 0.50 mmol), iron(III) triflate (0.025 g, 0.05 mmol) and sodium borohydride (0.378 g, 10.00 mmol) were reacted in anhydrous ethanol (4 mL). Crude product was obtained (83 %). Following purification by general procedure **F**, 4-(trifluoromethyl) aniline was isolated as an amorphous cream solid (0.075 g, 0.38 mmol, 76 %). δ_H (400 MHz, CD_3OD) 7.83 (d, J = 8.4 Hz, 2H), 7.52 (d, J = 8.4 Hz, 2H). δ_C (126 MHz, $CDCl_3$) 137.5, 133.0, 129.4, 125.8. IR υ_{max} (cm^{-1}): 2849, 2590, 2359, 1616, 1510, 1321, 1194, 1161, 1126, 1065, 1016, 827.

Data was in accordance with that previously reported.[19]

Table 4, entry 11

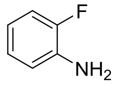

2-Fluoroaniline (6k)

According to general procedure **D**, 1-fluoro-2-nitrobenzene (0.053 mL, 0.50 mmol), iron(III) triflate (0.025 g, 0.05 mmol) and sodium borohydride (0.378 g, 10.00 mmol) were reacted anhydrous ethanol (4 mL). Crude product was obtained (73%). Product was not isolated.

Data were consistent with those of an authentic sample (Aldrich Cat. No. F3401).

Table 4, entry 12

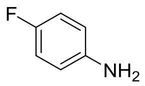

4-Fluoroaniline (6l)

According to general procedure **D**, 4-fluoro-1-nitrobenzene (0.070 g, 0.50 mmol), iron(III) triflate (0.025 g, 0.05 mmol) and sodium borohydride (0.378 g, 10.00 mmol) were reacted in anhydrous ethanol (4 mL). Crude product was obtained (87 %). Product was not isolated.

Data were consistent with those of an authentic sample (Aldrich Cat. No. F3800).

Table 4, entry 13

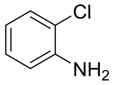

2-Chloroaniline (6m)

According to general procedure **D**, 1-chloro-2-nitrobenzene (0.064 g, 0.058 mL, 0.50 mmol), iron(III) triflate (0.025 g, 0.05 mmol) and sodium borohydride (0.378 g, 10.00 mmol) were reacted in anhydrous ethanol (4 mL). Crude product was obtained (87 %). Following purification by general procedure **E**, 2-chloroaniline was isolated as brown needles (0.011 g, 0.09 mmol, 17 %). δ_H (500 MHz, $CDCl_3$) 7.26 (dd, J = 8.0 Hz, 1.3 Hz, 1H), 7.09 (td, J = 8.0 Hz, 1.4 Hz, 1H), 6.79 (dd, J = 8.0 Hz, 1.4 Hz, 1H), 6.71 (td, J = 7.3 Hz, 1.5 Hz, 1H), 4.06 (br. s, 2H). δ_C (126 MHz, $CDCl_3$) 142.9, 129.4, 127.6, 119.3, 119.1, 115.9. IR υ_{max} (cm^{-1}): 2835.4, 2590.4, 2139.1, 1988.6, 1562.3, 1514.1, 1477.5, 1444.7, 1400.3, 1159.2, 1097.5, 1051.2, 754.2, 680.9.

Data were consistent with those of an authentic sample (Aldrich Cat. No. 23310).

Table 4, entry 14

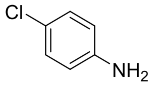

4-Chloroaniline (6n)

According to general procedure **D**, 1-chloro-4-nitrobenzene (0.079 g, 0.50 mmol), iron(III) triflate (0.025 g, 0.05 mmol) and sodium borohydride (0.378 g, 10.00 mmol) were reacted in anhydrous ethanol (4 mL). Crude product was obtained (80 %). Following purification by general procedure **E**, 4-chloroaniline was isolated as a brown plates (0.030 g, 0.24 mmol, 47 %). δ_H (500 MHz, $CDCl_3$) 7.11 (dt, J = 8.8 Hz, 2.2 Hz, 2H), 6.62 (dt, J = 8.8 Hz, 2.1 Hz, 2H), 3.62 (br s, 1H). δ_C (126 MHz, $CDCl_3$) 144.9, 129.1, 123.2, 116.2. IR υ_{max} (cm^{-1}): 3474, 3383, 2359, 2342, 1614, 1499, 1288, 1180, 1090, 829, 640.

Data were consistent with those of an authentic sample (Aldrich Cat. No. C22415).

Table 4, entry 15

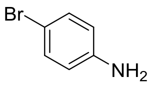

4-Bromoaniline (6o)

According to general procedure **D**, 1-bromo-4-nitrobenzene (0.101 g, 0.50 mmol), iron(III) triflate (0.025 g, 0.05 mmol) and sodium borohydride (0.378 g, 10.00 mmol) were reacted in anhydrous ethanol (4 mL). Crude product was obtained (53 %). Following flash column chromatography (dichloromethane, methanol) 4-bromoaniline was isolated as an orange amorphous solid (0.087 g, 0.51 mmol, 51%). δ_H (500 MHz, $CDCl_3$) 7.26 (dt, J = 8.9 Hz, 2.3 Hz, 2H), 6.58 (dt, J = 8.9 Hz, 2.3 Hz, 2H) 3.67 (br s, 2H). δ_C (126 MHz, $CDCl_3$). IR υ_{max} (cm^{-1}): 3471.9, 3381.2, 1610.6, 1593.2, 1487.1, 1284.6, 1180.4, 1151.5, 1068.6, 815.9.

Data were consistent with those of an authentic sample (Aldrich Cat. No.100900).

Table 4, entry 16

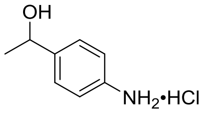

1-(4-Aminophenyl)ethanol hydrochloride (6p)

According to general procedure **D**, 1-(4-nitrophenyl)ethanone (0.083 g, 0.50 mmol), iron(III) triflate (0.025 g, 0.05 mmol) and sodium borohydride (0.378 g, 10.00 mmol) were reacted in anhydrous ethanol (4 mL). Crude product was obtained (68 %). Following purification by general procedure **F**, 1-(4-Aminophenyl)ethanol hydrochloride was isolated (0.013 g, 0.08 mmol, 15%). δ_H(400 MHz, CD_3OD) 7.50 (d, J = 8.3 Hz, 2H), 7.39 (d, J = 8.6 Hz, 2H), 4.41 (q, J = 6.5 Hz, 1H), 1.40 (d, J = 6.5 Hz, 3H). δ_C (100 MHz, CD_3OD) 146.72, 131.10, 129.20, 124.36, 80.11, 24.12. IR υ_{max} (cm^{-1}): 3383, 2577, 2455, 2361, 2342, 1578, 1514, 1420, 1206, 1067, 827.

Data was in accordance with that previously reported.[20]

Table 4, entry 17

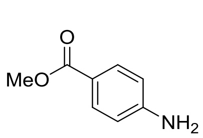

Methyl 4-aminobenzoate (6q)

According to general procedure **D**, methyl 4-nitrobenzoate (0.181 g, 1.00 mmol), iron(III) triflate (0.050 g, 0.10 mmol) and sodium borohydride (0.757 g, 20.00 mmol) were reacted in anhydrous ethanol (8 mL). Crude product was obtained (87 %). Following purification by general procedure **E**, methyl 4-aminobenzoate was isolated (0.121 g, 0.80 mmol, 80%). δ_H (400 MHz: $CDCl_3$) 7.86 (dt, J = 8.6 Hz, 1.8 Hz, 2H), 6.66 (dt, J = 8.7 Hz, 1.9 Hz, 2H), 3.86 (s, 3H). δ_C (150 MHz: $CDCl_3$) 167.1, 150.8, 131.6, 119.8, 113.8, 51.6. IR υ_{max} (cm^{-1}): 3339, 1682, 1597, 1275, 1173, 1111.

Data were consistent with those of an authentic sample (Aldrich Cat. No.274186).

Table 4, entry 18

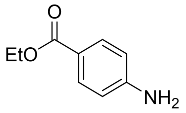

Ethyl 4-aminobenzoate (6r)

According to general procedure **D**, ethyl 4-nitrobenzoate (0.181 g, 1.00 mmol), iron(III) triflate (0.050 g, 0.10 mmol) and sodium borohydride (0.757 g, 20.00 mmol) were reacted in anhydrous ethanol (8 mL). Crude product was obtained (93%). Following purification by general procedure **E**, ethyl 4-aminobenzoate was isolated as an (0.046 g, 0.28 mmol, 28%), mp 87-88 °C. δ_H (400 MHz: $CDCl_3$) 7.85 (dt, J = 8.7 Hz, 1.9 Hz, 2H), 6.63 (dt, J = 8.7 Hz, 1.9 Hz, 2H), 4.31 (q, J = 7.2 Hz, 2H), 4.07 (s, 2H), 1.37 (t, J = 7.0 Hz, 3H). δ_C (150 MHz: $CDCl_3$) 166.7, 150.7, 131.5, 120.1, 113.7, 60.3, 14.4. IR υ_{max} (cm^{-1}): 3341, 1680, 1595, 1275, 1171, 1109.

Data was in accordance with that previously reported.[20]

Table 4, entry 19

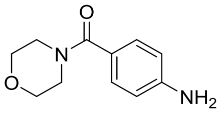

4-(4-Aminobenzoyl)morpholine (6s)

According to general procedure **D**, 4-(4-nitrobenzoyl)morpholine (0.130 g, 0.55 mmol), iron(III) triflate (0.028 g, 0.05 mmol) and sodium borohydride (0.416 g, 11.00 mmol) were reacted in anhydrous ethanol (4.25 mL). Crude product was obtained (60 %). Following purification by general procedure **E**, 4-(4-aminobenzoyl)morpholine was isolated as a pale brown amorphous solid (0.067 g, 0.32 mmol, 32%). δ_H(600 MHz: $CDCl_3$) 7.29 (d, J = 8.3 Hz, 2H), 6.68 (d, J = 8.3 Hz, 2H), 3.89 (br s, 2H), 3.72 (br s, 4H), 3.67 (br s, 4H). δ_C (150 MHz: $CDCl_3$) 170.9, 148.3, 129.4, 124.7, 114.2, 67.0, 65.82.

Data was in accordance with that previously reported.[21]

Table 4, entry 20

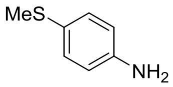

4-(Methyl)-aniline (6t)

According to general procedure **D**, 4-nitrothioanisole (0.169 g, 1.00 mmol), iron(III) triflate (0.050 g, 0.10 mmol) and sodium borohydride (0.757 g, 20.00 mmol) were reacted in anhydrous ethanol (8 mL). Crude product was obtained (100 %). Following purification by general procedure **E**, 4-(methyl)-aniline was isolated as an amorphous orange solid (0.103 g, 0.76 mmol, 76 %). δ_H(500 MHz, $CDCl_3$) 7.21 (dt, J = 8.7 Hz, 2.1 Hz, 2H), 6.66 (dt, J = 8.7 Hz, 2.1 Hz, 2H), 3.67 (br s, 2H), 2.44 (s, 3H). δ_C(150 MHz, $CDCl_3$) 145.1, 131.1, 125.8, 115.7, 18.8.

Data was in accordance with that previously reported.[18]

Table 4, entry 21

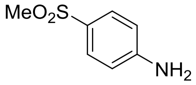

4-(Methylsulfonyl)-aninline (6u)

According to general procedure **D**, 4-nitrothioanisole (0.123 g, 1.00 mmol), iron(III) triflate (0.050 g, 0.10 mmol) and sodium borohydride (0.757 g, 20.00 mmol) were reacted in anhydrous ethanol (8 mL). Crude product was obtained (100 %). Following purification by general procedure **E**, 4-(methyl)-aniline was isolated as an amorphous orange solid (0.103 g, 0.76 mmol, 76 %). δ_H(500 MHz, $CDCl_3$) 7.73 (dt, *J* = 8.8 Hz, 2.0 Hz, 2H), 6.74 (dt, *J* = 8.8 Hz, 2.1 Hz, 2H), 4.20 (br s, 2H), 3.03 (s, 3H). δ_C(150 MHz, $CDCl_3$) 151.2, 129.5, 129.0, 114.1, 45.0.

Data was in accordance with that previously reported.[18]

Table 4, entry 22

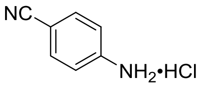

4-Aminobenzonitrile hydrochloride (6v)

According to general procedure **B**, 4-nitrobenzonitrile (0.074 g, 0.50 mmol), iron(III) triflate (0.025 g, 0.05 mmol) and sodium borohydride (0.378 g, 10.00 mmol) were reacted in anhydrous ethanol (4 mL). Crude product was obtained (82 %). Following purification by general procedure **C**, 4-aminobenzonitrile hydrochloride was isolated as an amorphous dark yellow solid (0.060 g, 0.39 mmol, 77 %), mp 200-202 °C. δ_H (400 MHz, CD_3OD) 7.77 (d, *J* = 8.8 Hz, 2H), 7.31 (d, *J* = 8.8 Hz, 2H). δ_C(100 MHz, CD_3OD) 142.08, 135.31, 122.48, 119.54, 109.66. IR υ_{max} (cm^{-1}): 2808, 2577, 2361, 2236, 2102, 1609, 1503, 1371, 835, 812.

Data was in accordance with that previously reported.[18]

Table 4, entry 23

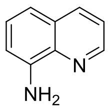

8-Aminoquinoline (6w)

According to general procedure **A**, 8-nitroquinoline (0.0871 g, 0.50 mmol), iron(III) triflate (0.025 g, 0.05 mmol) and sodium borohydride (0.378 g, 10.00 mmol) were reacted in anhydrous ethanol (4 mL). Crude product was obtained (54 %). The reaction mixture was diluted with water (5 mL) then treated with sodium cyanide (0.015 g, 0.30 mmol). The resultant aqueous phase was extracted with EtOAc (3 ×10 mL) then concentrated in vacuo. Following flash column chromatography (1:9 ethyl acetate:petroleum spirit) 8-aminoquinoline was isolated as a brown plates (0.037 g, 0.25 mmol, 51%) , mp: 57-60 °C. δ_H (400 MHz, $CDCl_3$) 8.77 (dd, *J* = 4.1 Hz, 1.7 Hz, 1H), 8.08 (dd, *J* = 8.3 Hz, 1.7 Hz, 1H), 7.40-7.32 (m, 2H), 7.16 (dd, *J* = 8.1 Hz, 1.2 Hz, 1H), 6.94 (dd, *J* = 7.5 Hz, 1.3 Hz, 1H), 4.99 (br s, 2H). δ_C (100 MHz, $CDCl_3$) 147.45, 143.96, 138.47, 136.00, 128.87, 127.38, 121.35, 116.07, 110.05. IR υ_{max} (cm^{-1}): 3451, 3348, 3034, 2924, 2359, 1591, 1504, 1368, 1094, 818, 789, 760.

Data were consistent with those of an authentic sample (Aldrich Cat. No.260789).

Table 4, entry 24

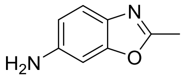

2-Methyl-6-benzoxazolamine (6x)

According to general procedure **A**, 2-methyl-6-nitrobenzoxazole (0.089 g, 0.50 mmol), iron(III) triflate (0.025 g, 0.05 mmol) and sodium borohydride (0.378 g, 10.00 mmol) were reacted in anhydrous ethanol (4 mL). Crude product was obtained (33 %). Following flash column chromatography (3:7 ethyl acetate:hexane) 2-methyl-6-benzoaxazolamine was isolated as brown prisms (0.018 g, 0.12 mmol, 24%) mp: 133-137 °C. δ_H (400 MHz, $CDCl_3$) 7.40 (d, *J* = 8.4 Hz, 1H), 6.78 (d, *J* = 1.9 Hz, 1H), 6.65 (dd, *J* = 8.4 Hz, 2.2 Hz, 1H), 2.57 (s, 3H). δ_C (100 MHz, $CDCl_3$) 161.69, 152.19, 144.25, 134.01, 119.48, 112.50, 96.47, 14.36. IR υ_{max} (cm^{-1}): 3362, 3213, 2359, 2342, 1614, 1576, 1493, 1435, 818, 557.

Data was in accordance with that previously reported.[22]

Table 4, entry 25

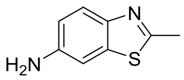

2-Methyl-6-benzothiazolamine (6y)

According to general procedure **C**, 2-methyl-6-nitro-benzothiazole (0.097 g, 0.50 mmol), iron(III) triflate (0.025 g, 0.05 mmol) and sodium borohydride (0.378 g, 10.00 mmol) were reacted in anhydrous ethanol (4 mL). Crude product was obtained (60 %). Following flash column chromatography (1:1 ethyl acetate:hexane) 2-methyl-6-benzothiazolamine was isolated (0.046 g, 0.28 mmol, 56%). δ_H (500 MHz, $CDCl_3$) 7.72 (d, *J* = 8.7 Hz, 1H), 7.06 (d, *J* = 2.2 Hz, 1H), 6.80 (dd, *J* = 8.6 Hz, 2.3 Hz, 1H), 3.80 (br s, 2H), 2.76 (s, 3H). δ_C (500 MHz, $CDCl_3$) 162.6, 146.7, 144.0, 137.2, 122.7, 155.1, 105.8, 19.8.

Data was in accordance with that previously reported.[23]

Table 4, entry 26

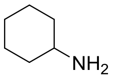

Cyclohexylamine (6z)

According to general procedure **C**, nitrocyclohexane (0.129 g, 1.00 mmol), iron(III) triflate (0.251 g, 0.50 mmol) and sodium borohydride (1.136 g, 30.00 mmol) were reacted in anhydrous ethanol (12 mL). Crude product was obtained (20 %). Product was not isolated.

Data were consistent with those of an authentic sample (Aldrich Cat. No.240648).

NMR Spectra of Compounds

4-(3-Butenyl)-benzoic acid methyl ester (1f)

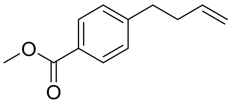

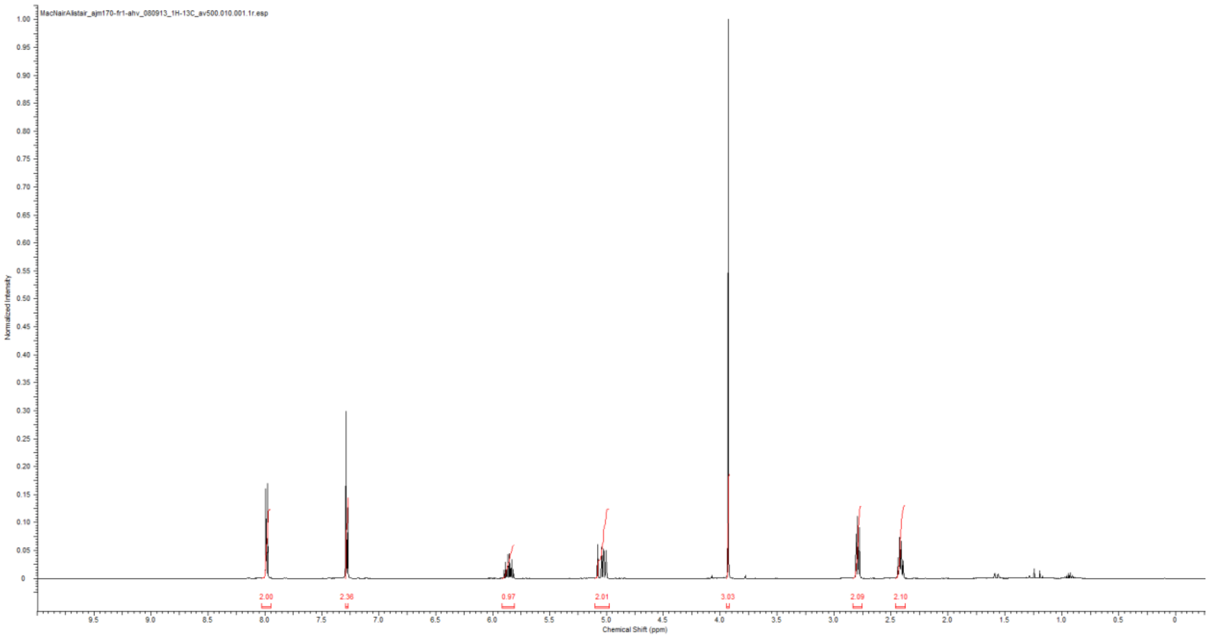

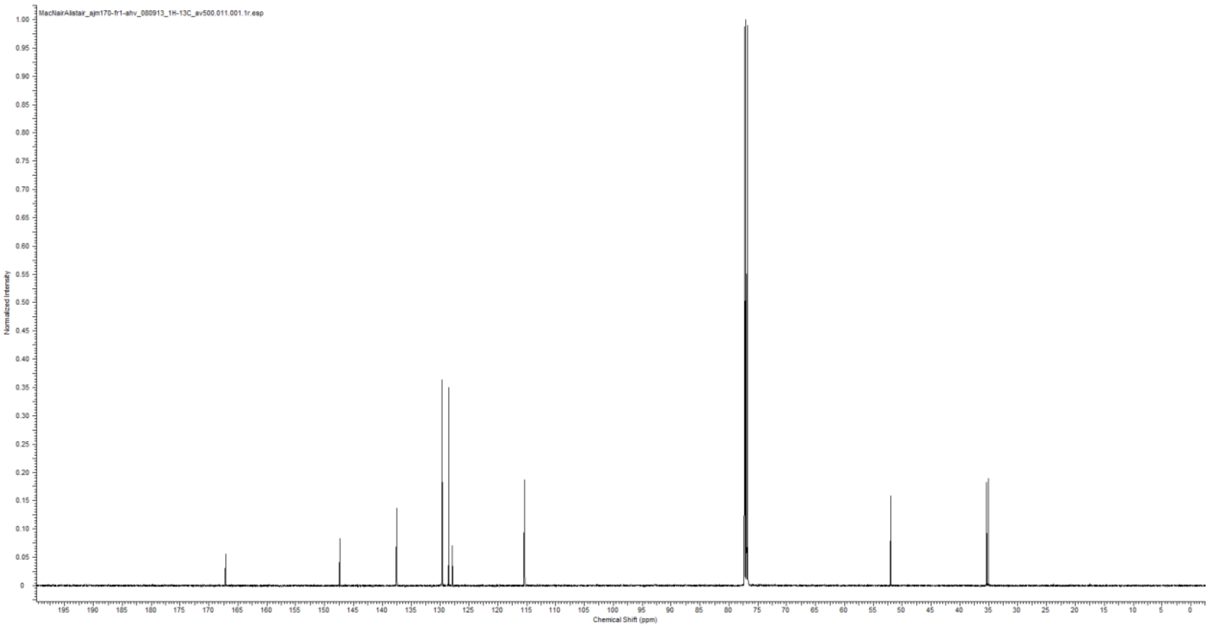

1-Benzyloxy-1-phenyl-3-butene (1i)

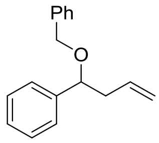

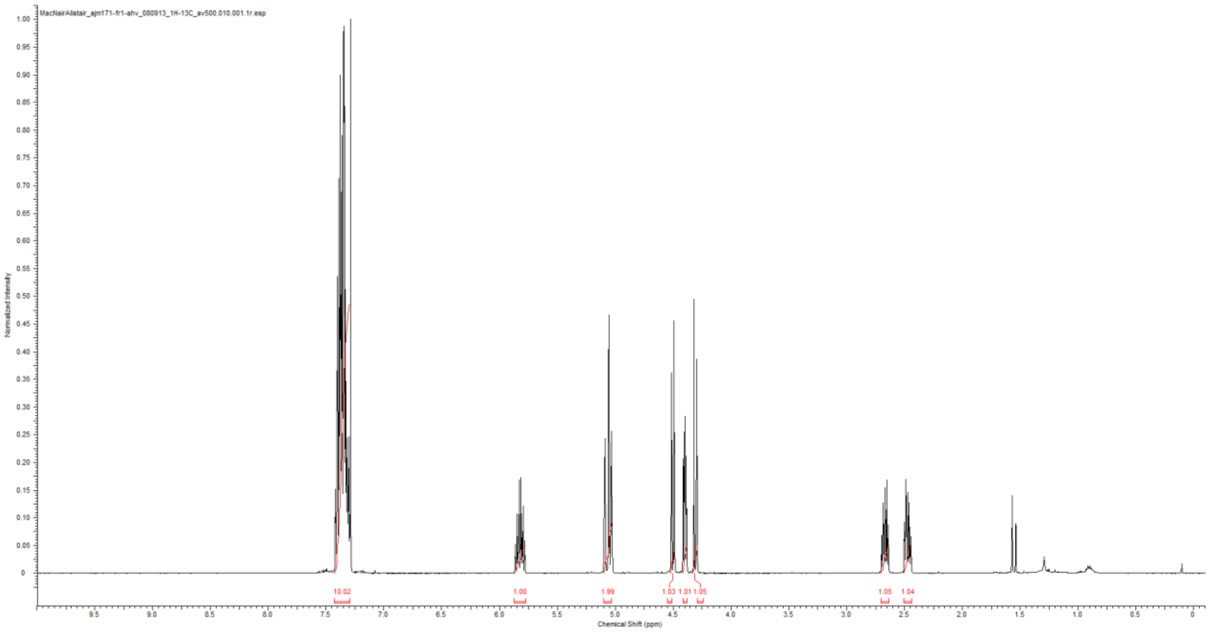

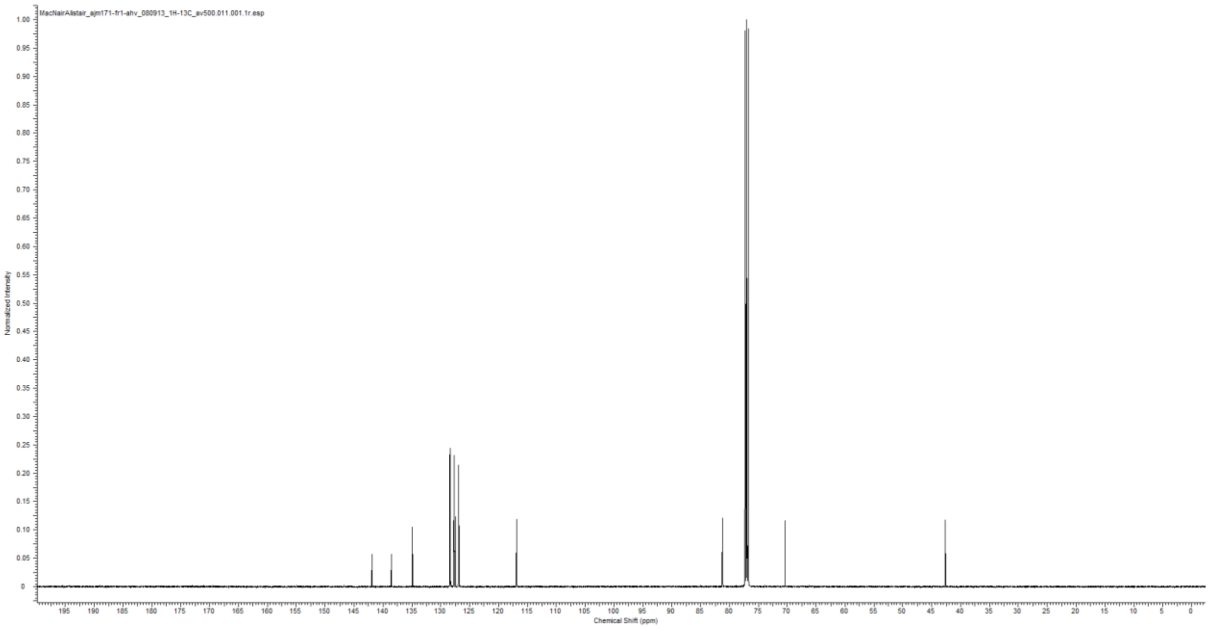

1-(4-Methylphenyl)-4-penten-1-one (1l)

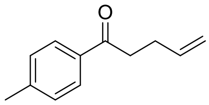

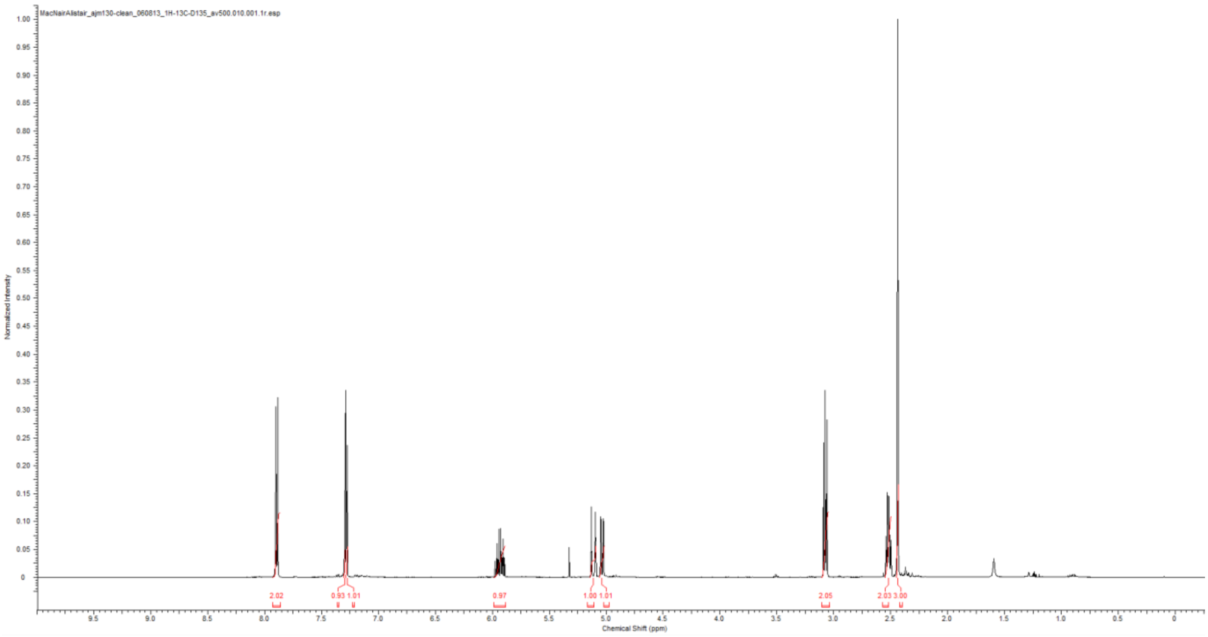

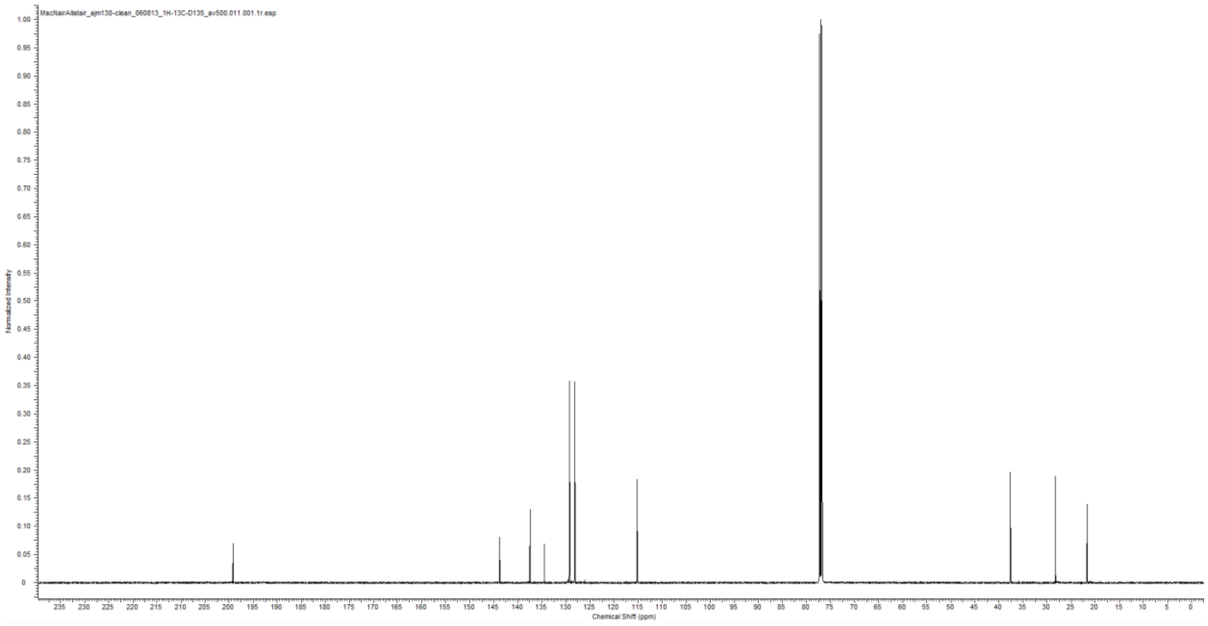

1-(4-Methylphenyl)-4-penten-1-ol (1m)

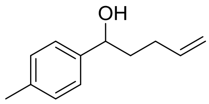

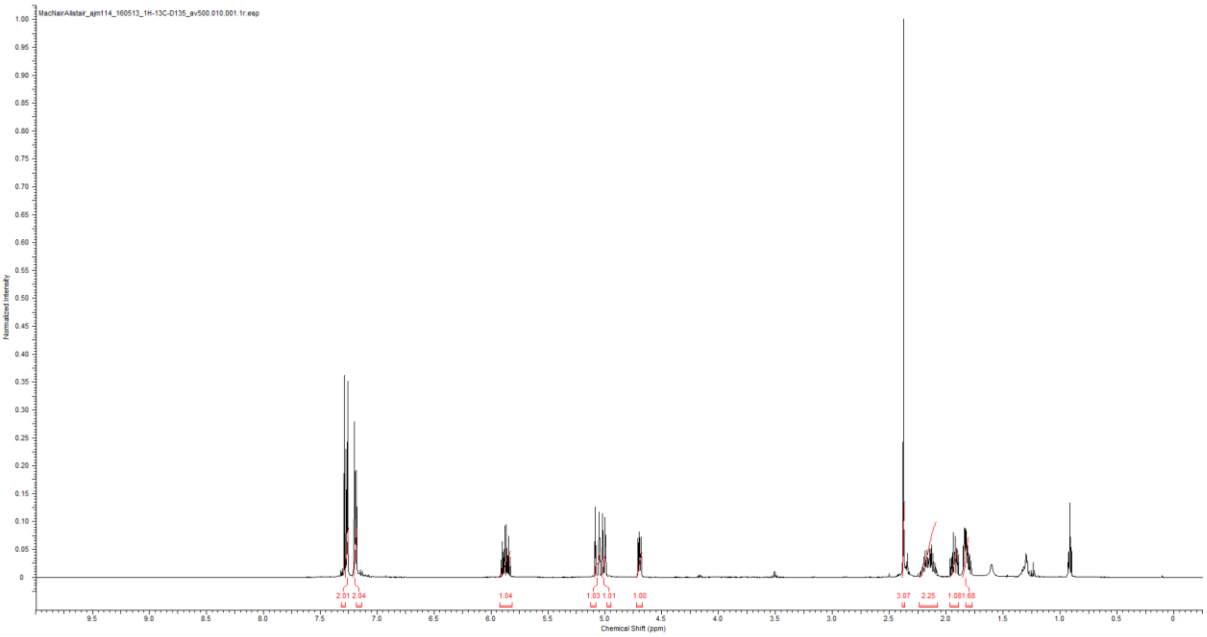

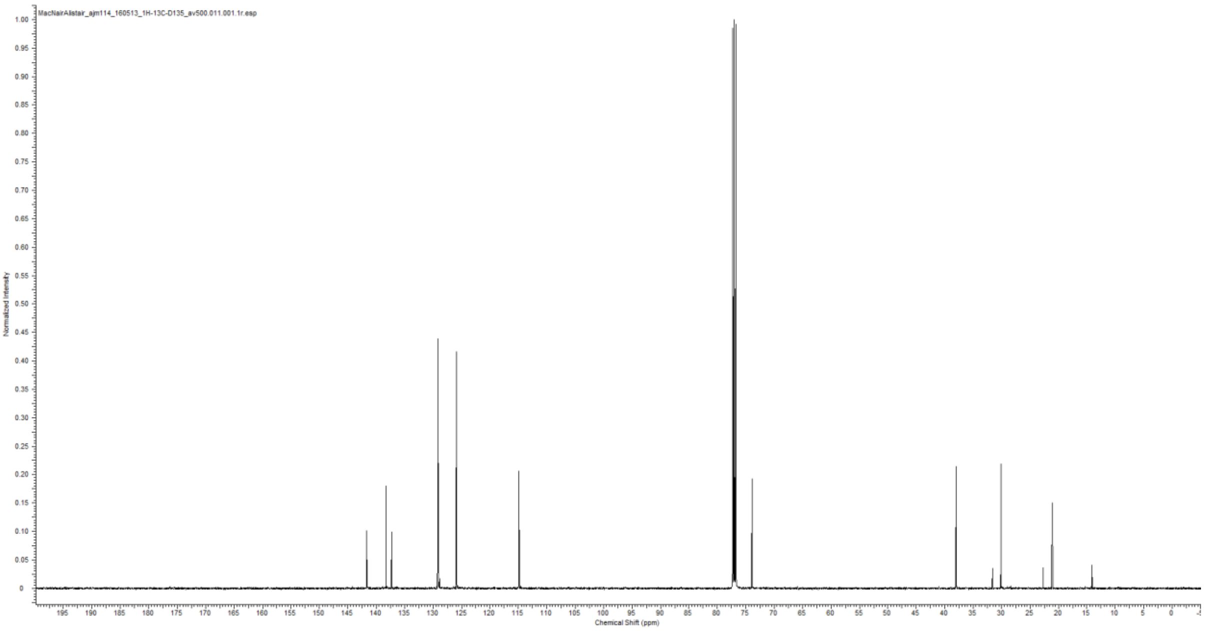

Methyl 4-nitrobenzoate (5q)

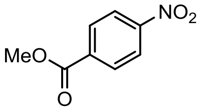

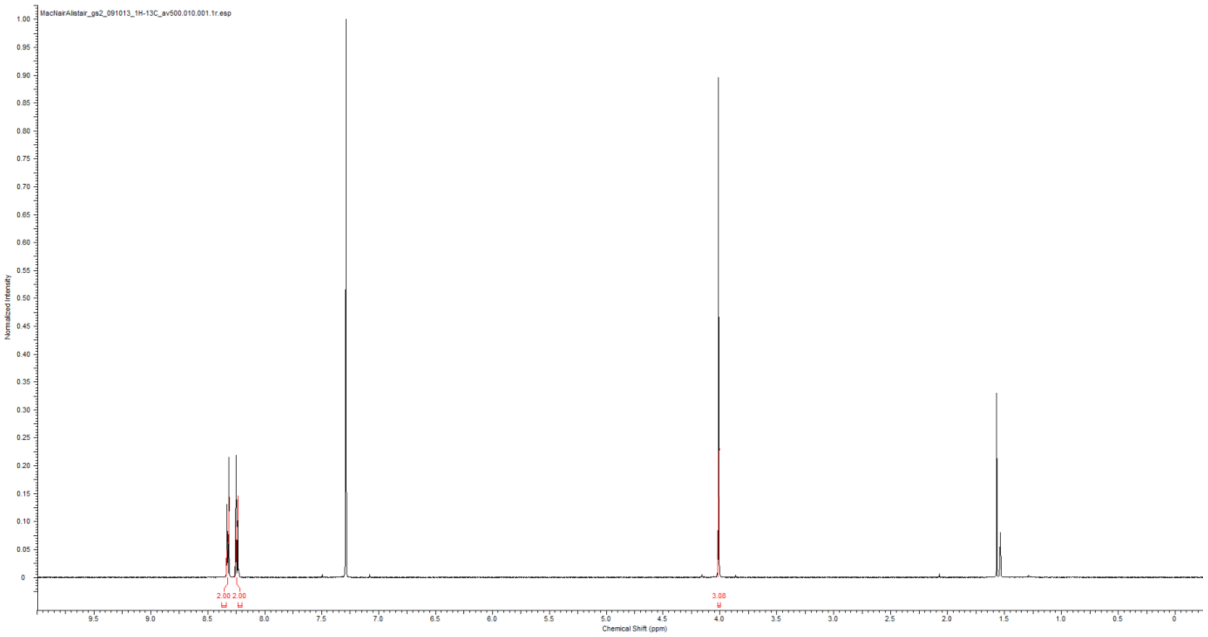

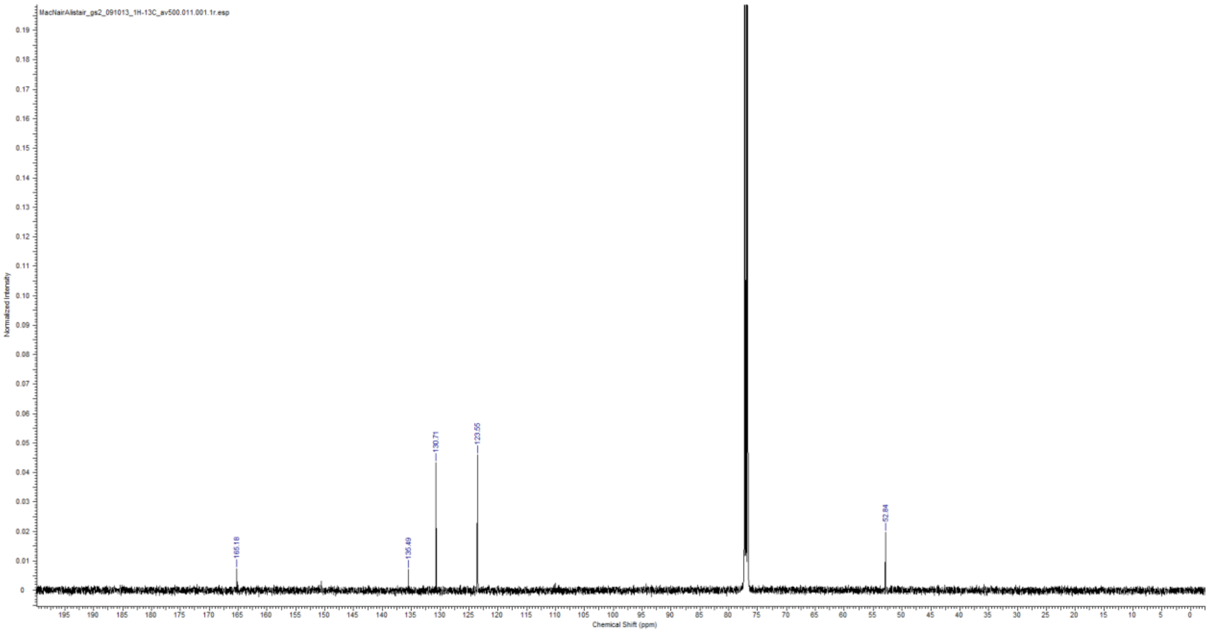

Ethyl 4-nitrobenzoate (5r)

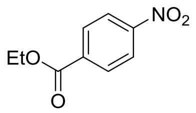

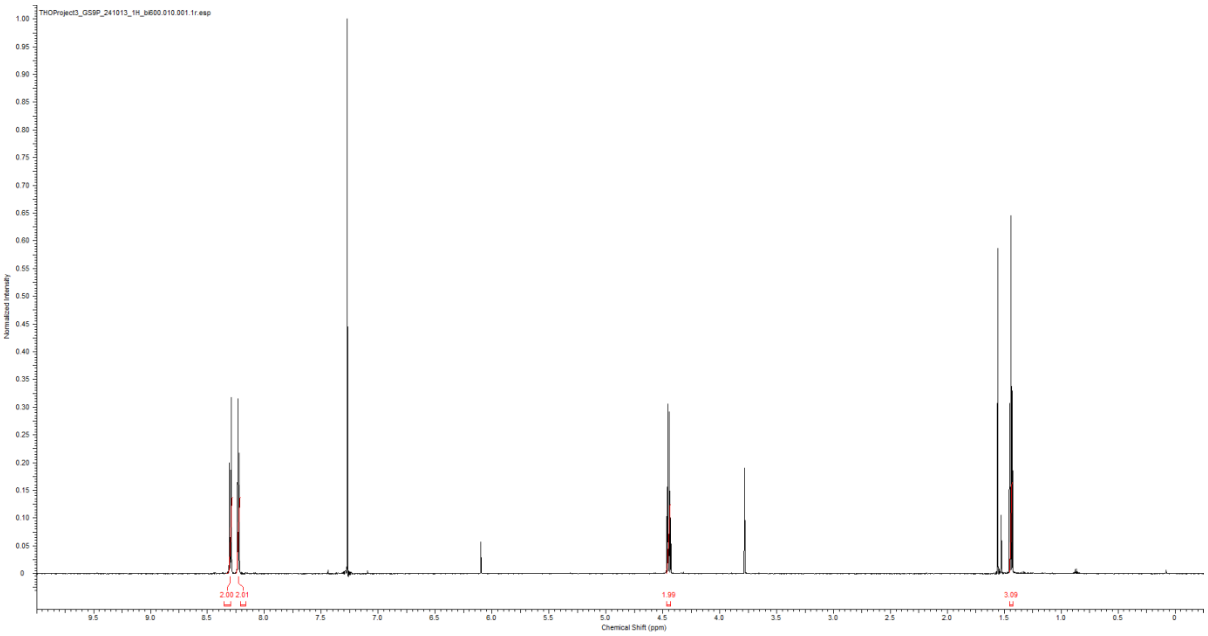

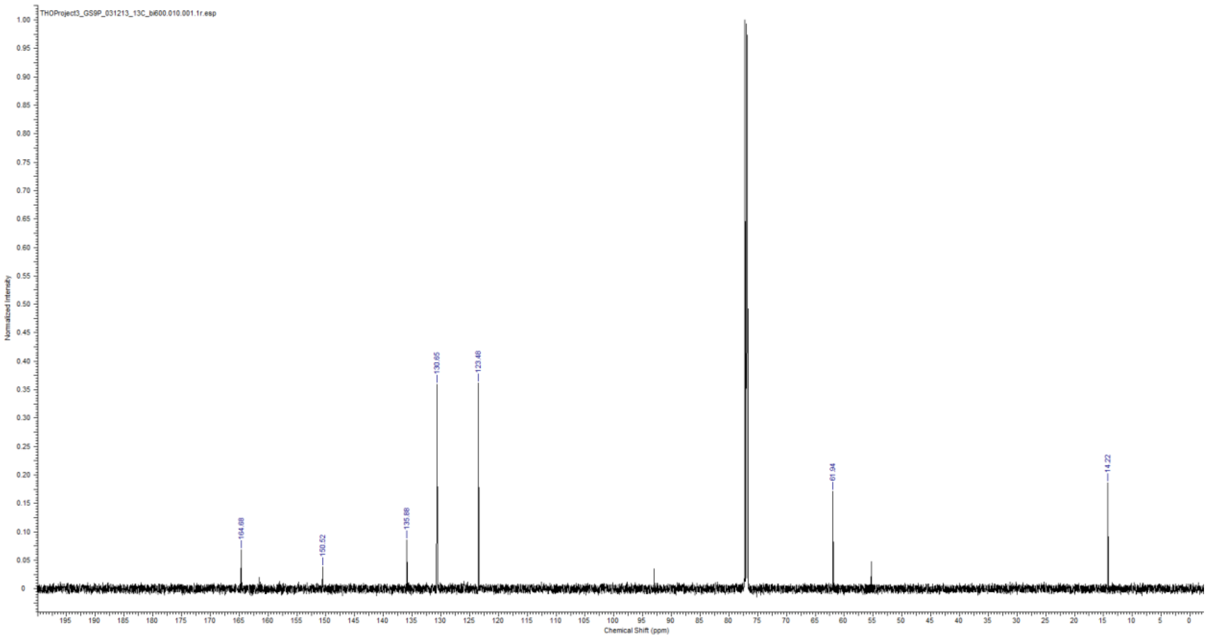

4-(4-Nitrobenzoyl)morpholine (5s)

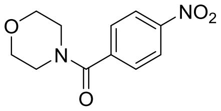

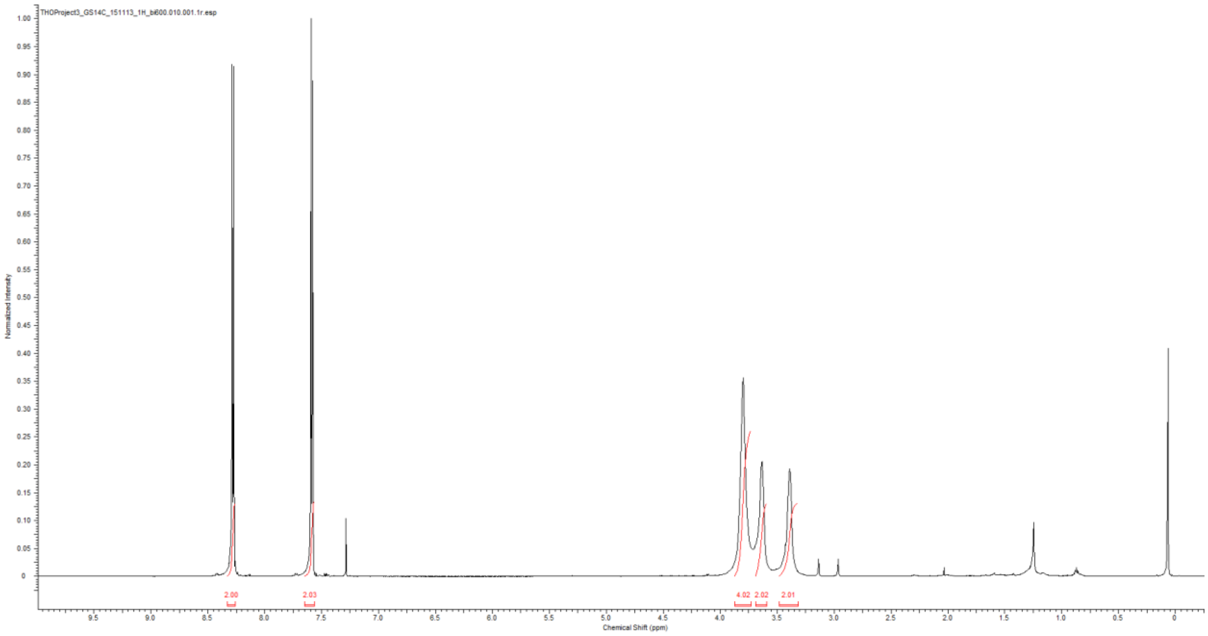

1-(Methylsulfonyl)-4-nitro-benzene (5u)

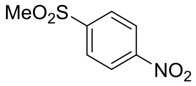

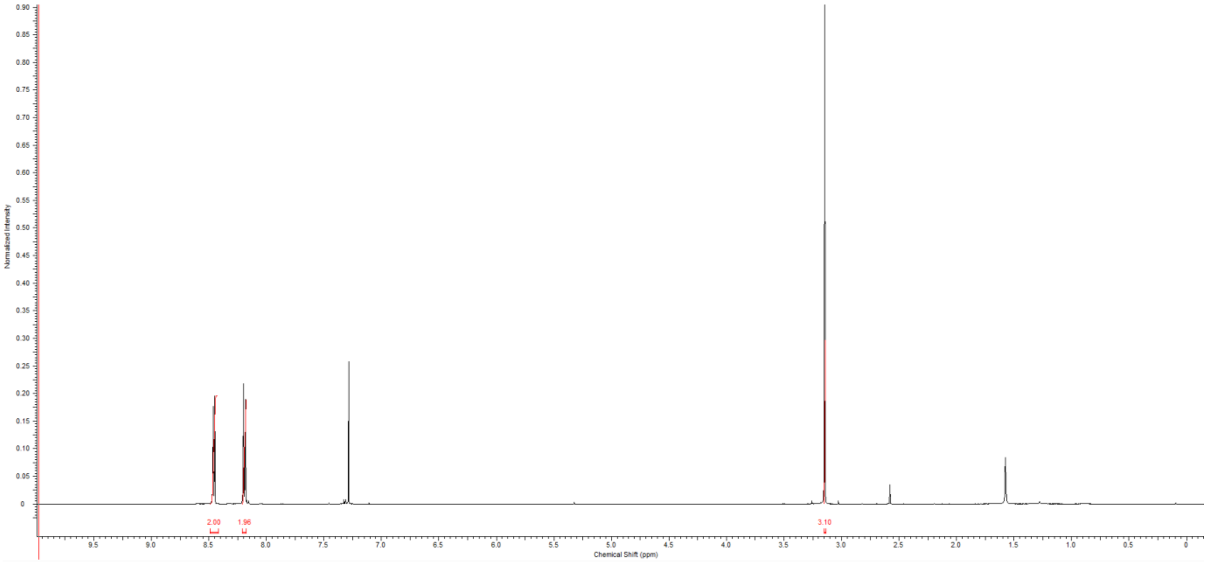

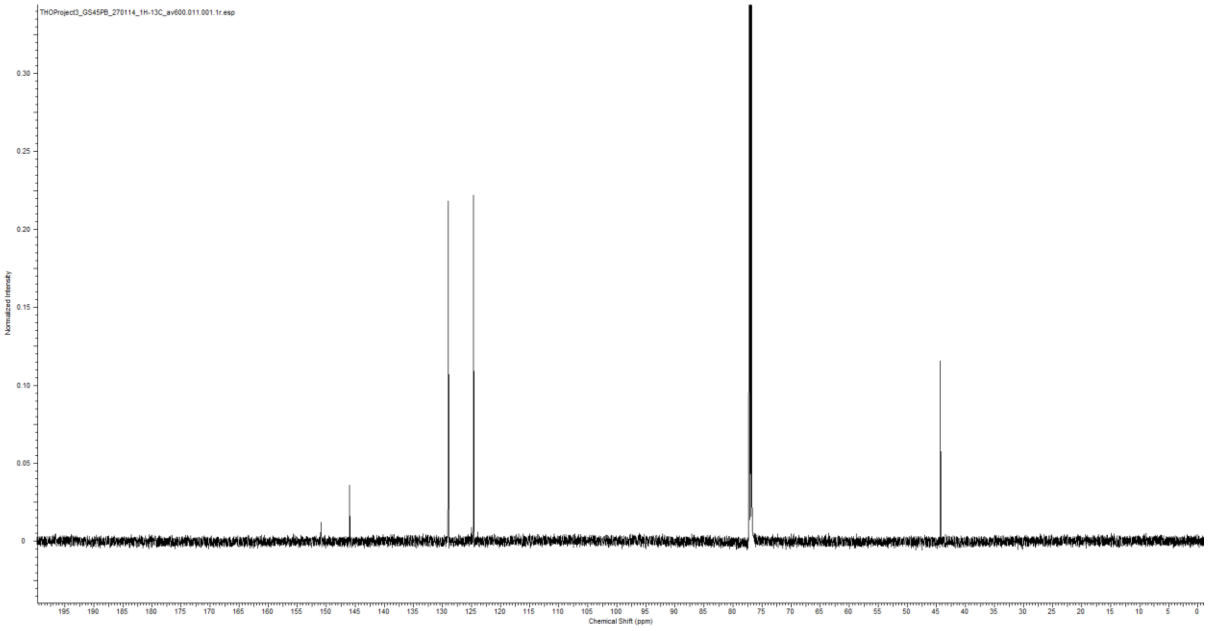

Butylbenzene (2a)

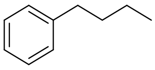

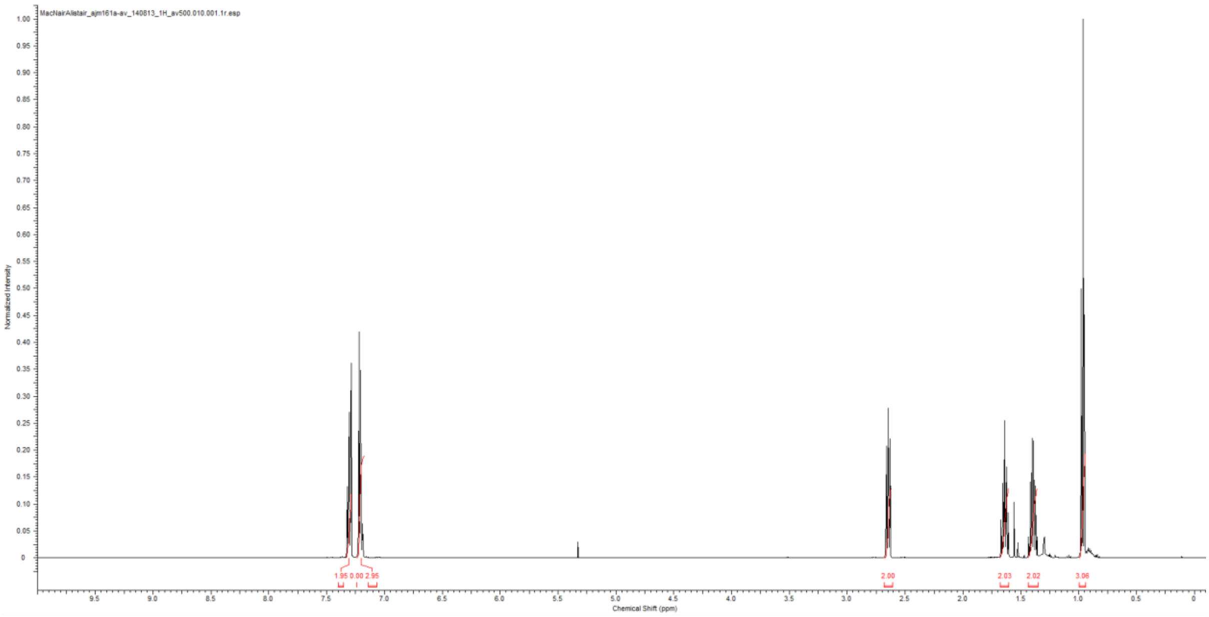

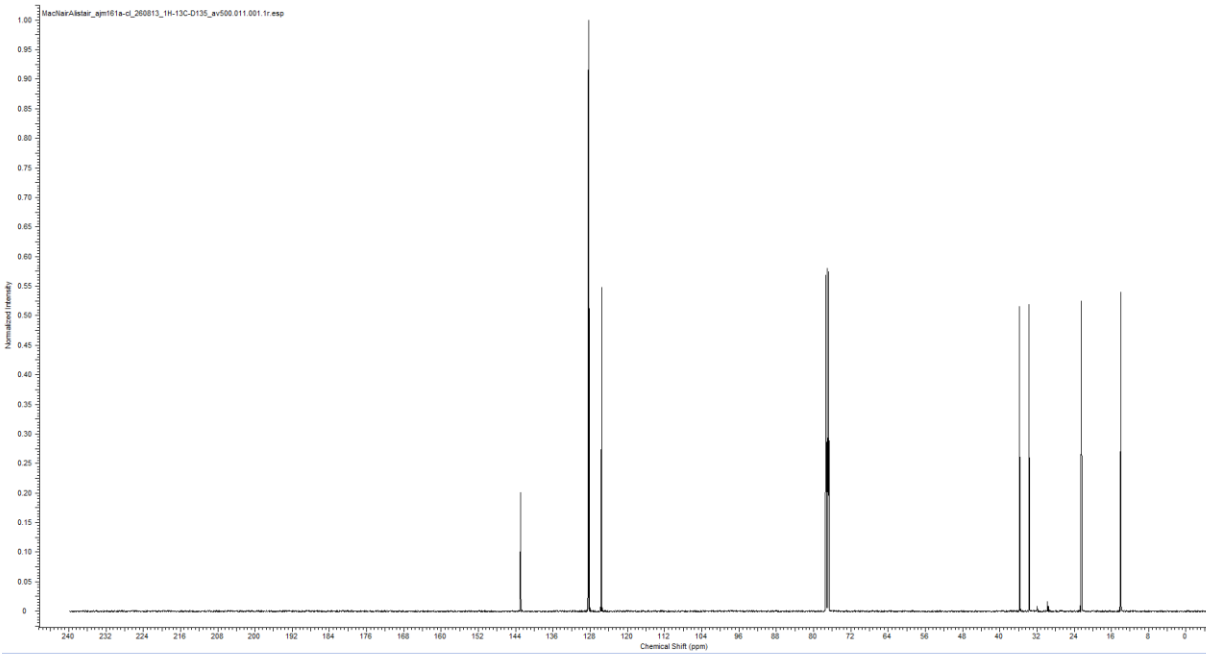

1-Fluoro-4-butylbenzene (2b)

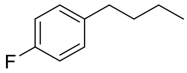

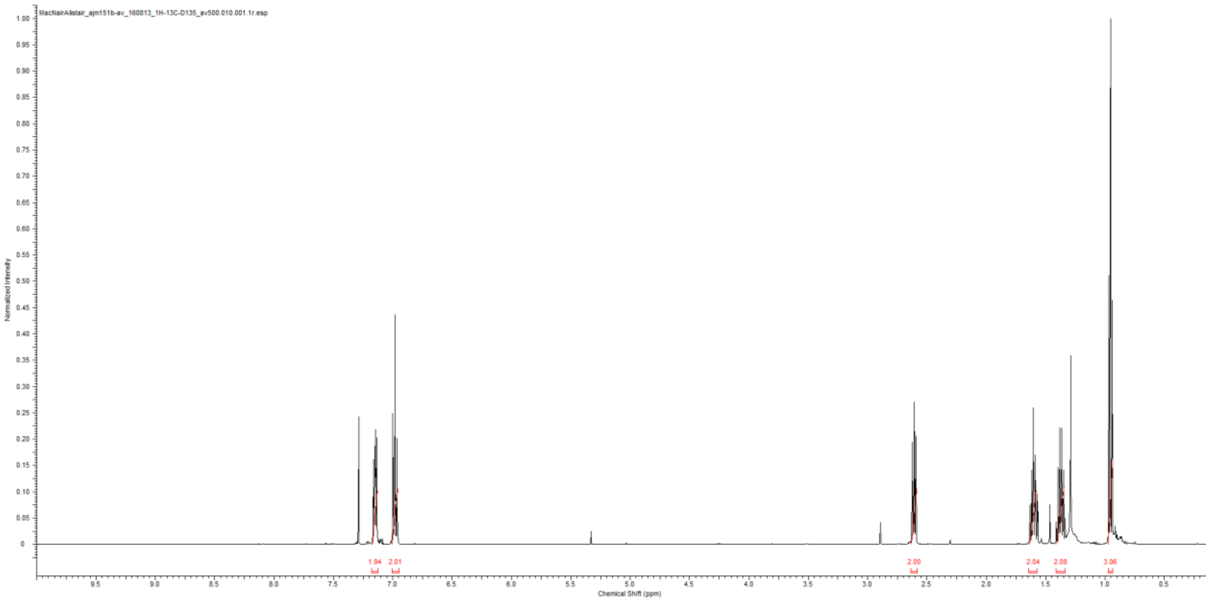

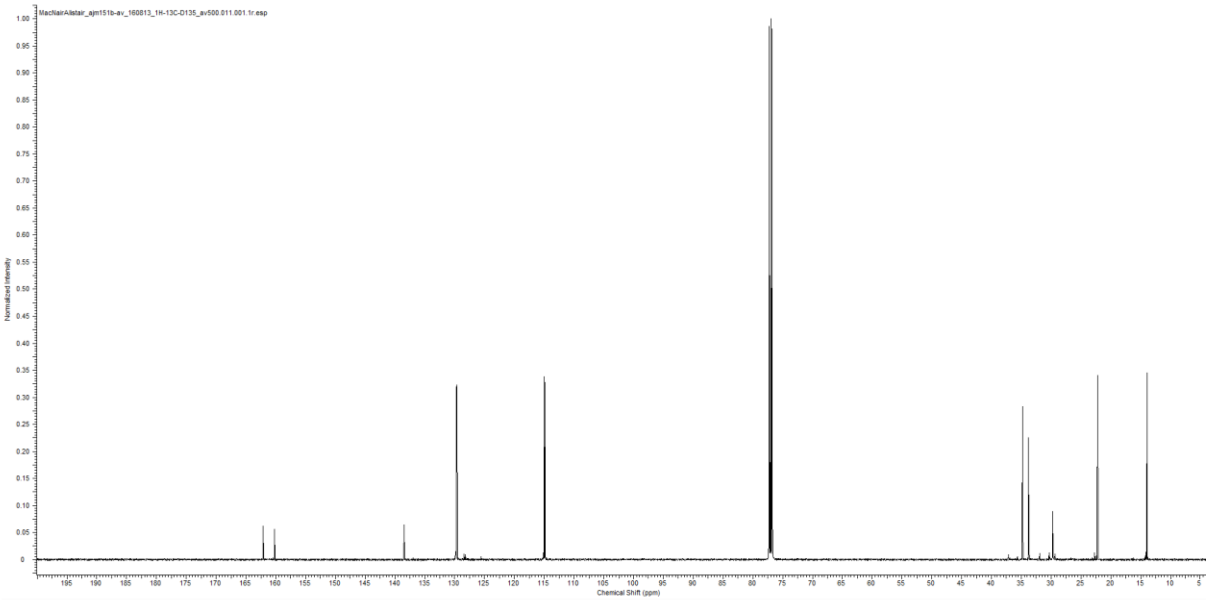

1-Chloro-4-butylbenzene (2c)

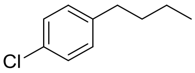

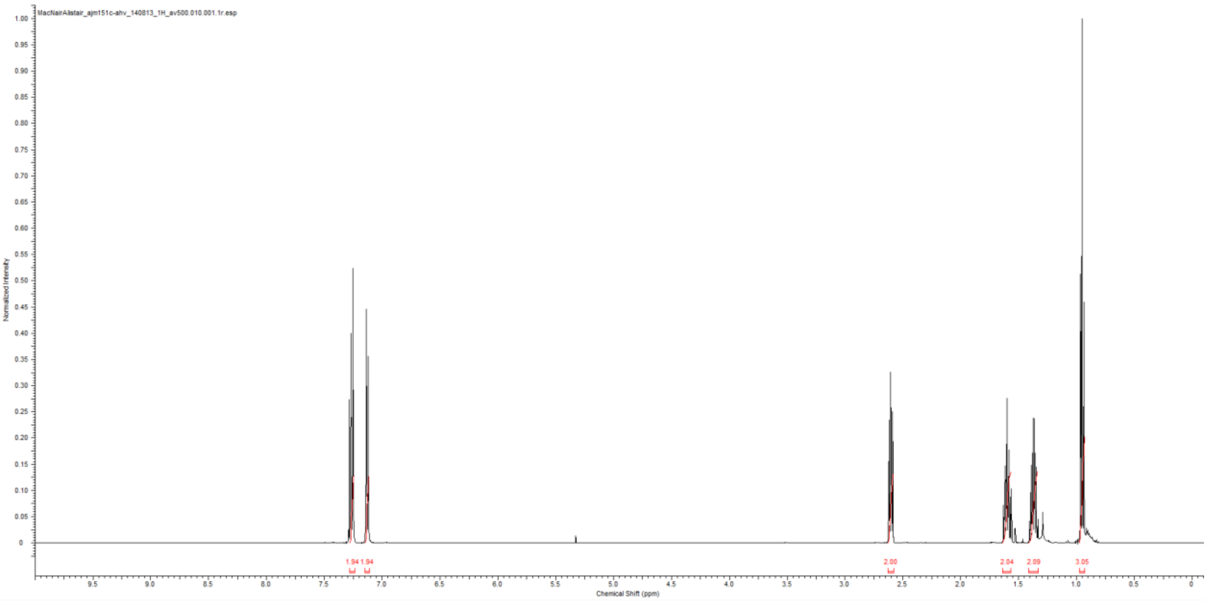

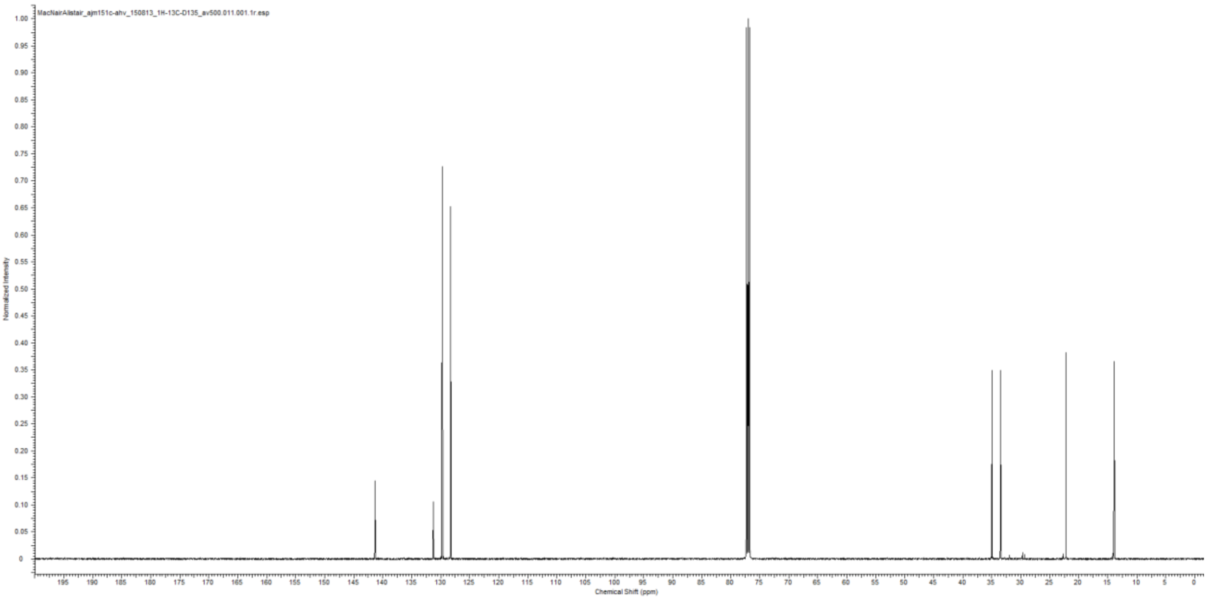

1-Bromo-4-butylbenzene (2d)

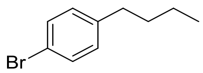

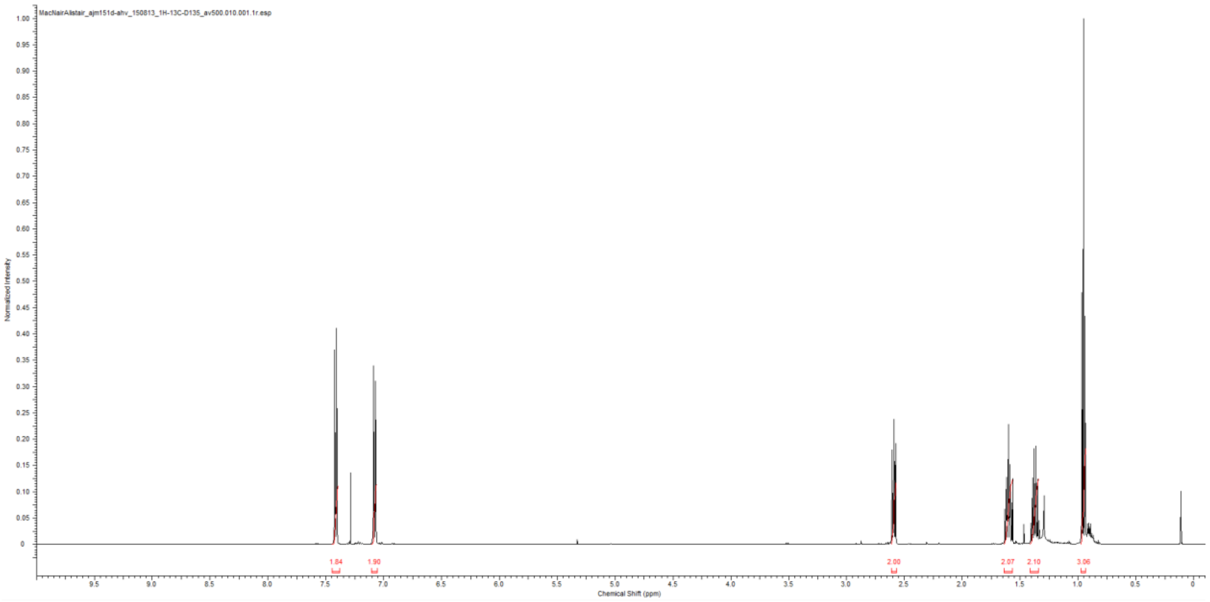

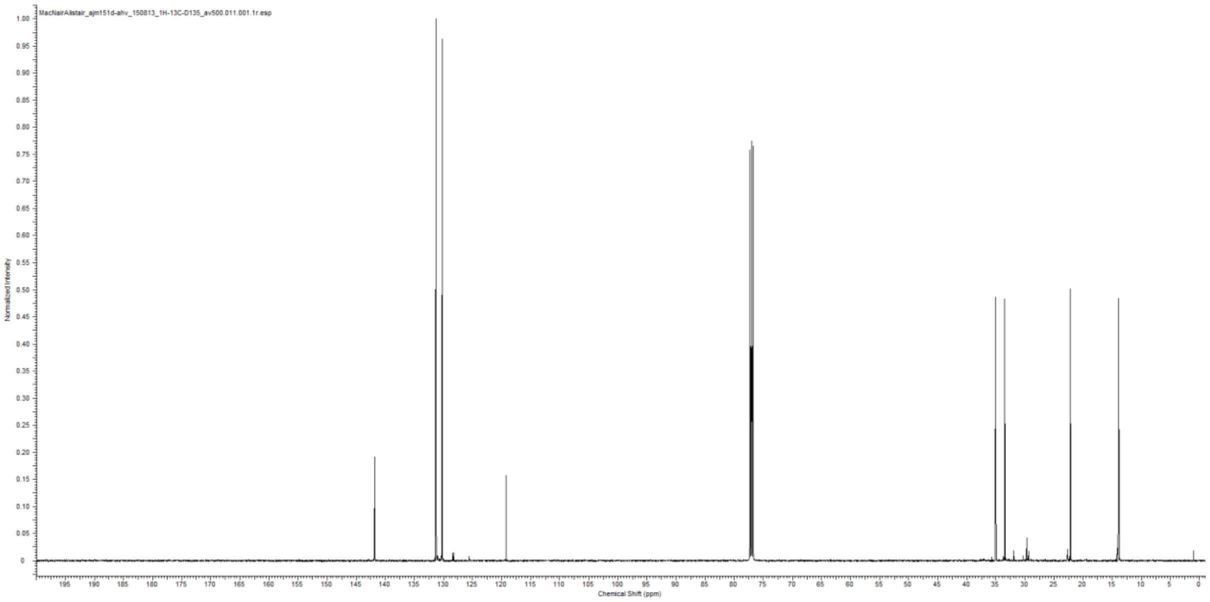

4-Butyl-benzoic acid, methyl ester (2f)

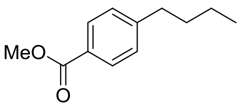

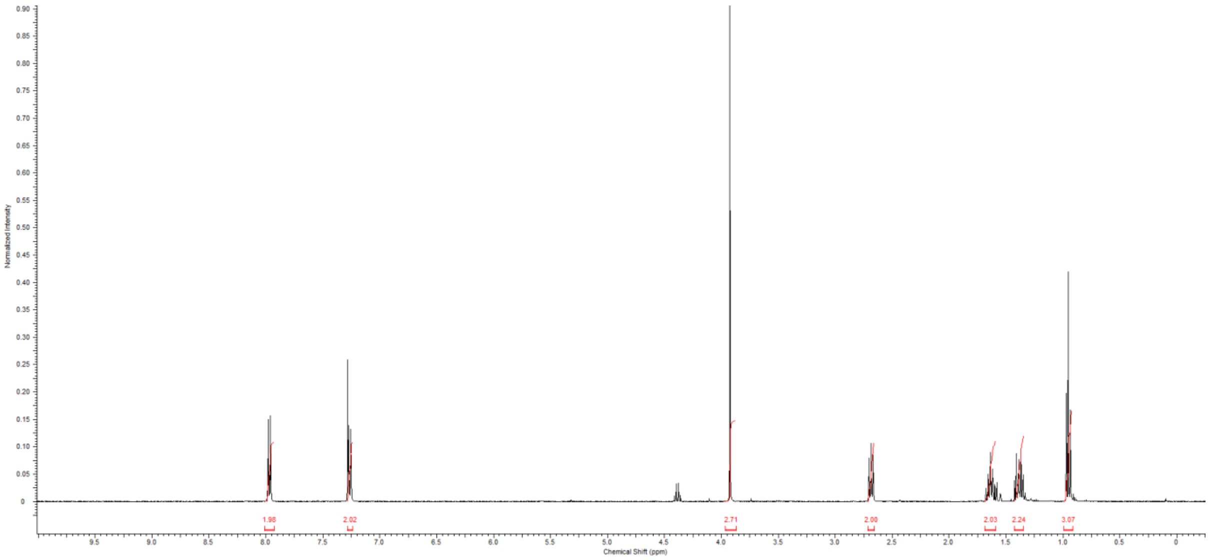

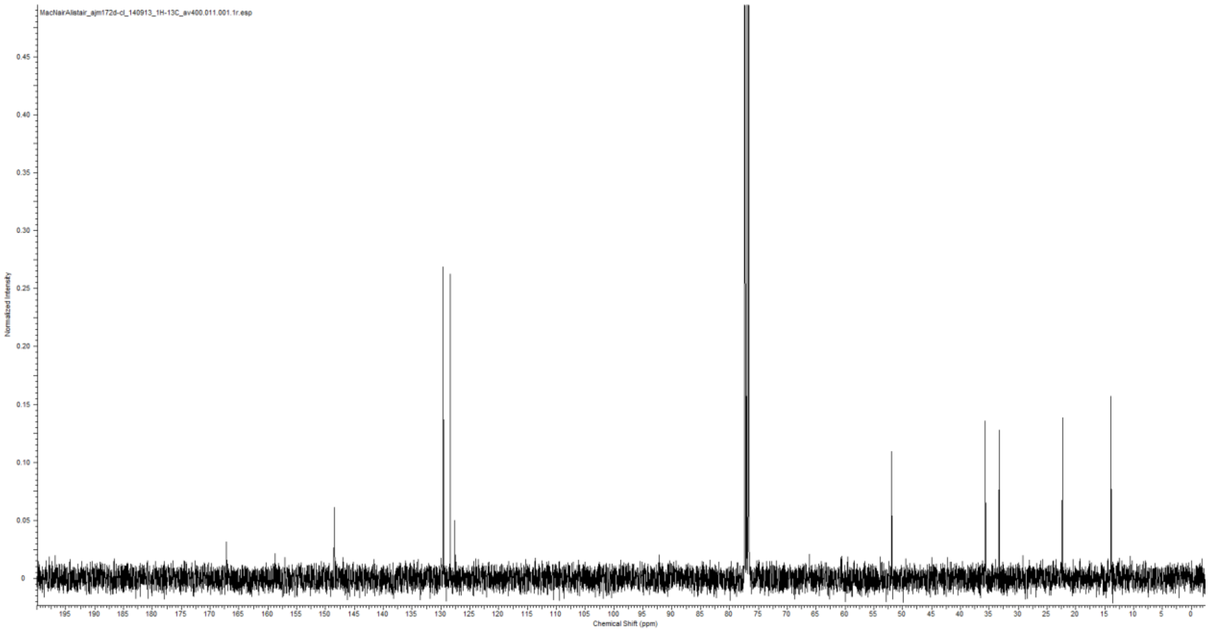

1-(4-Butylbenzoyl)-piperidine (2h)

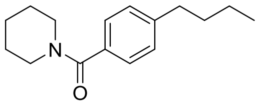

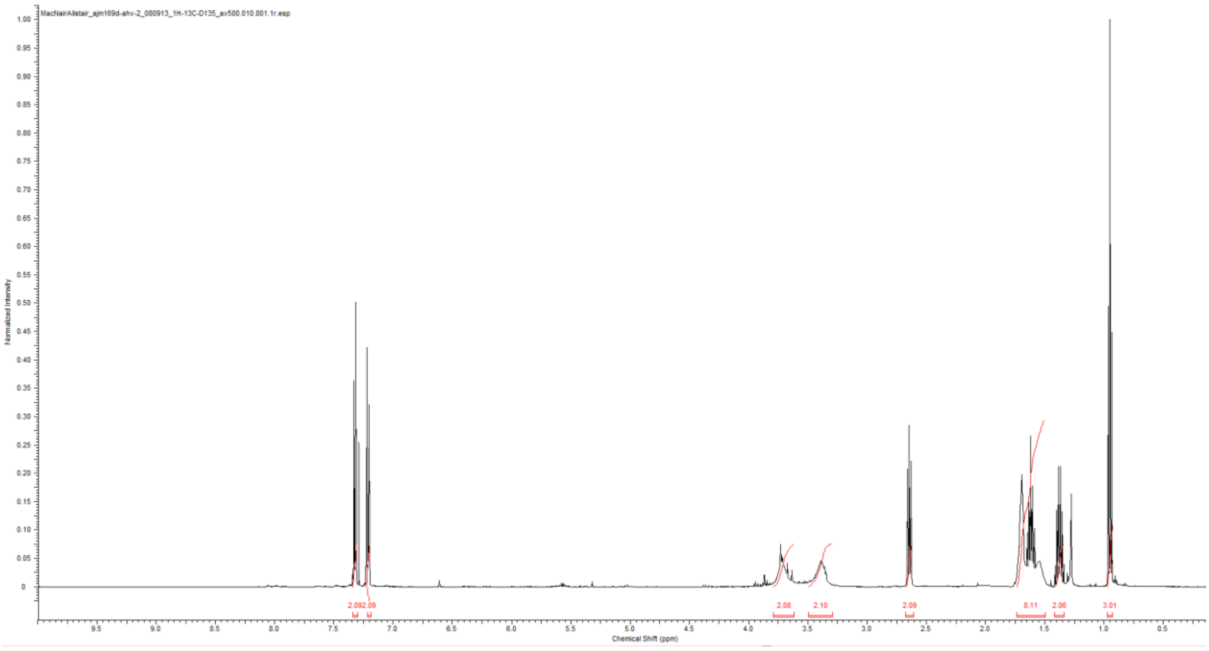

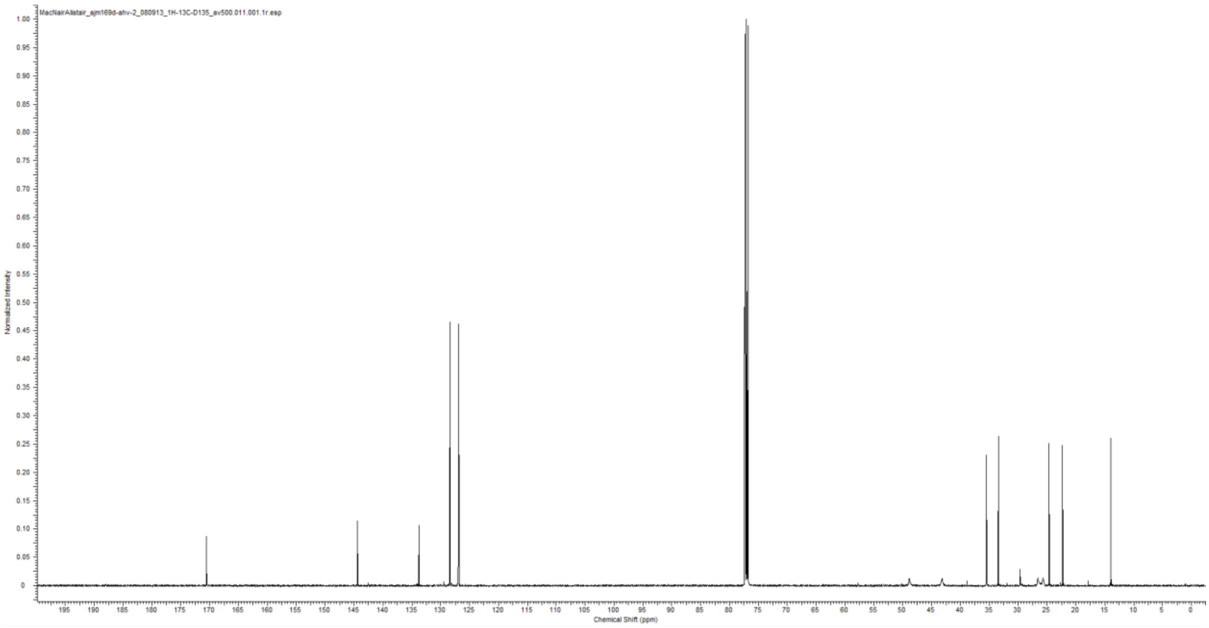

1-Benzyloxy-1-phenylbutane (2i)

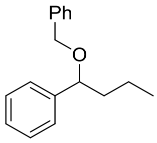

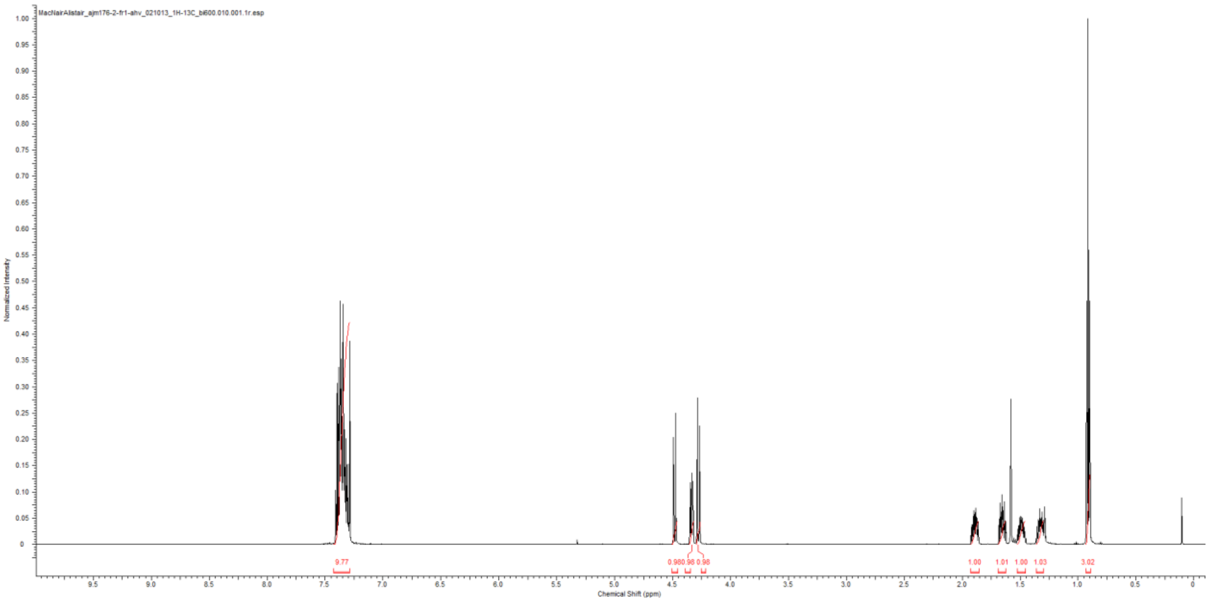

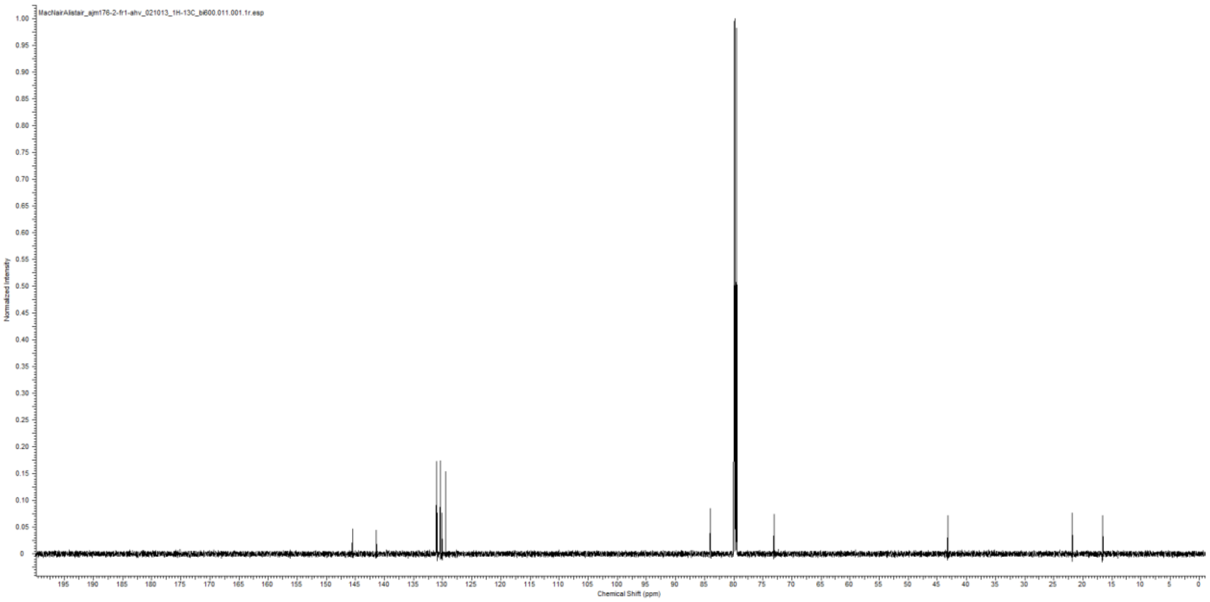

Propylcyclohexane (2k)

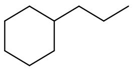

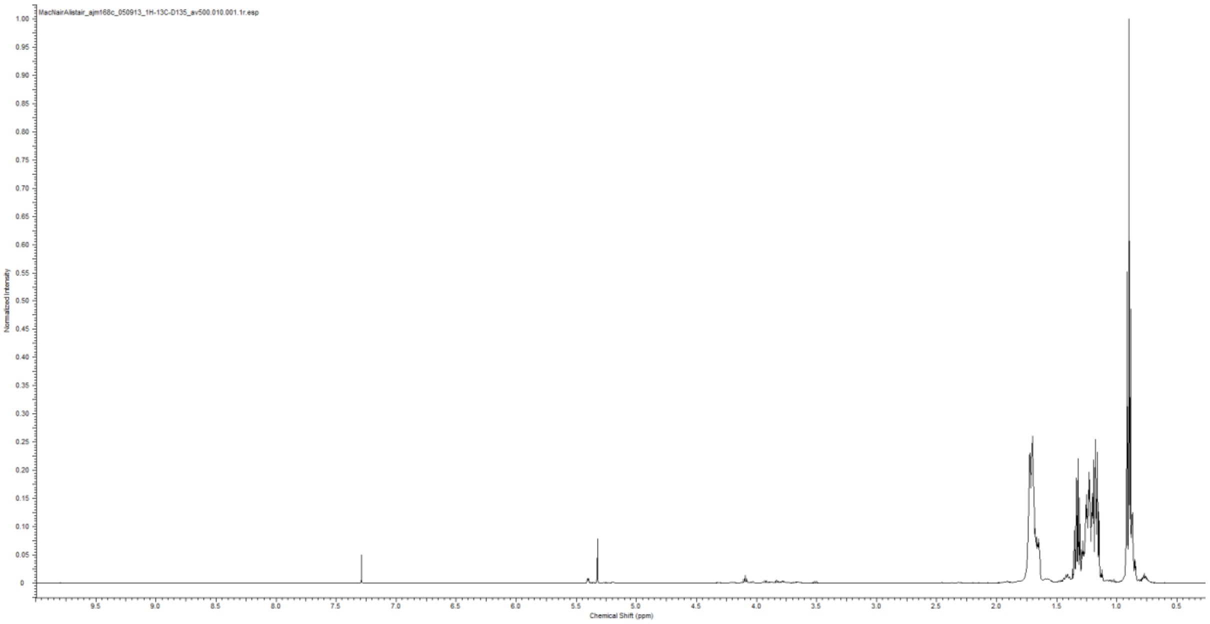

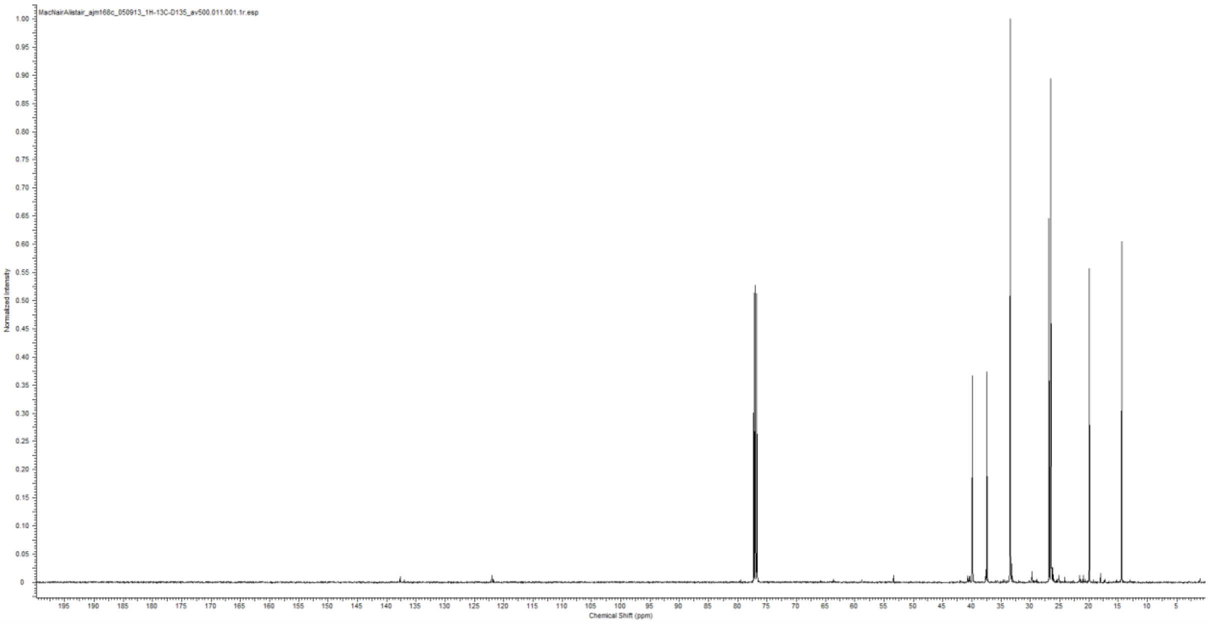

1-Ethyl-4-chlorobenzene (2n)

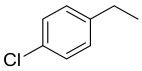

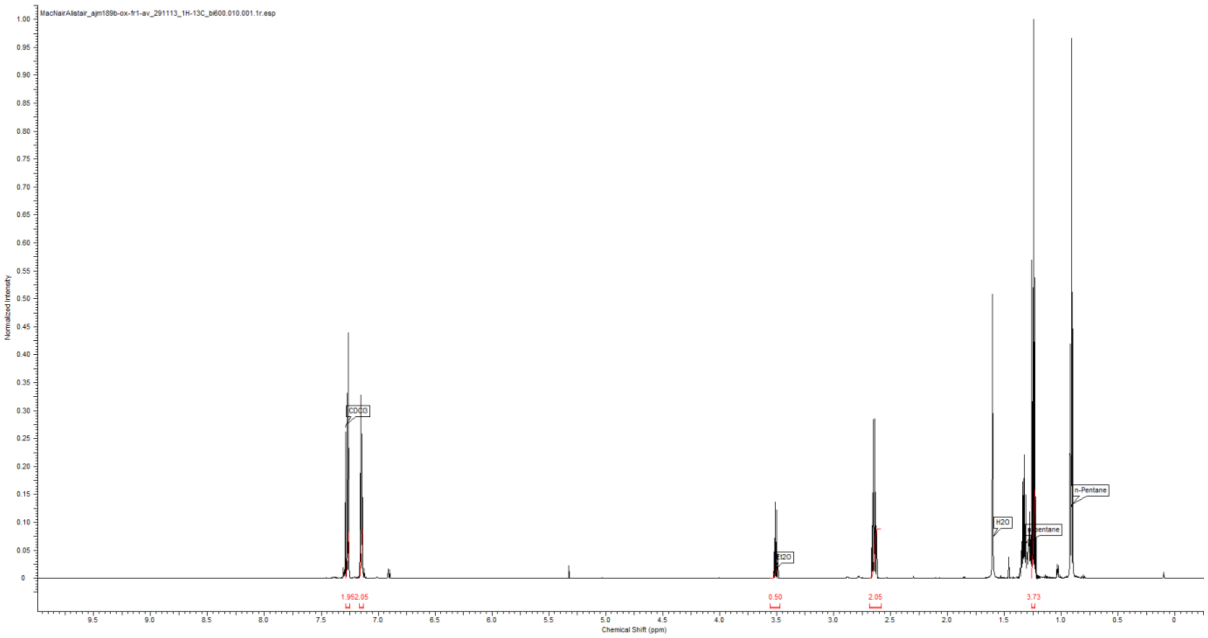

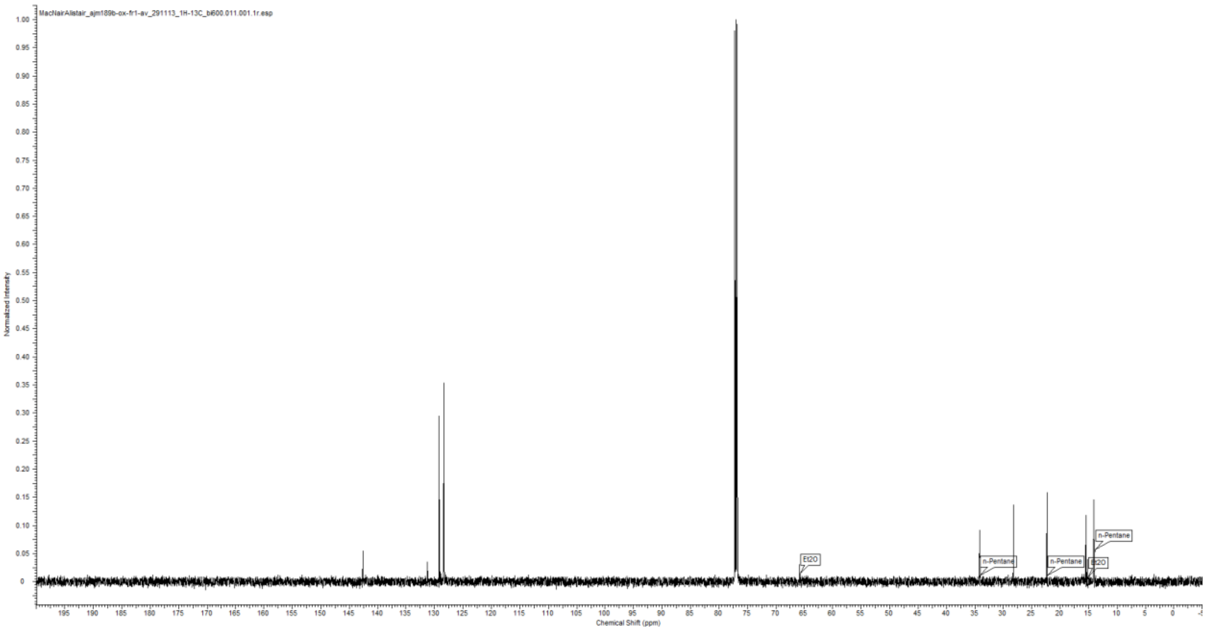

1-Ethyl-4-*tert*-butylbenzene (2o)

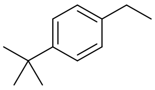

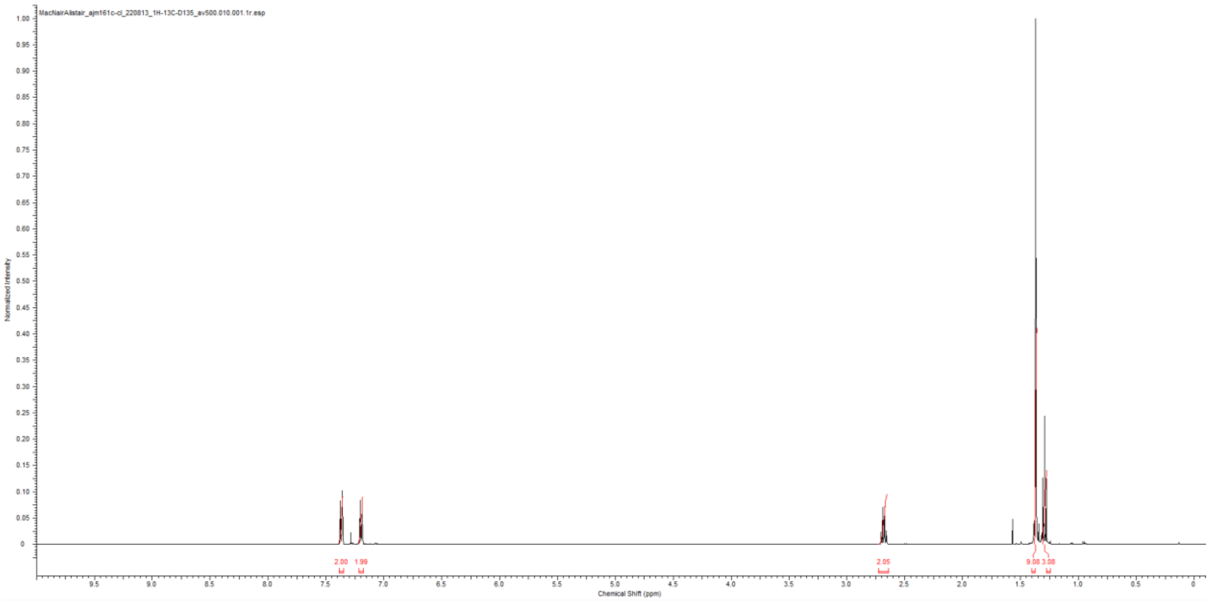

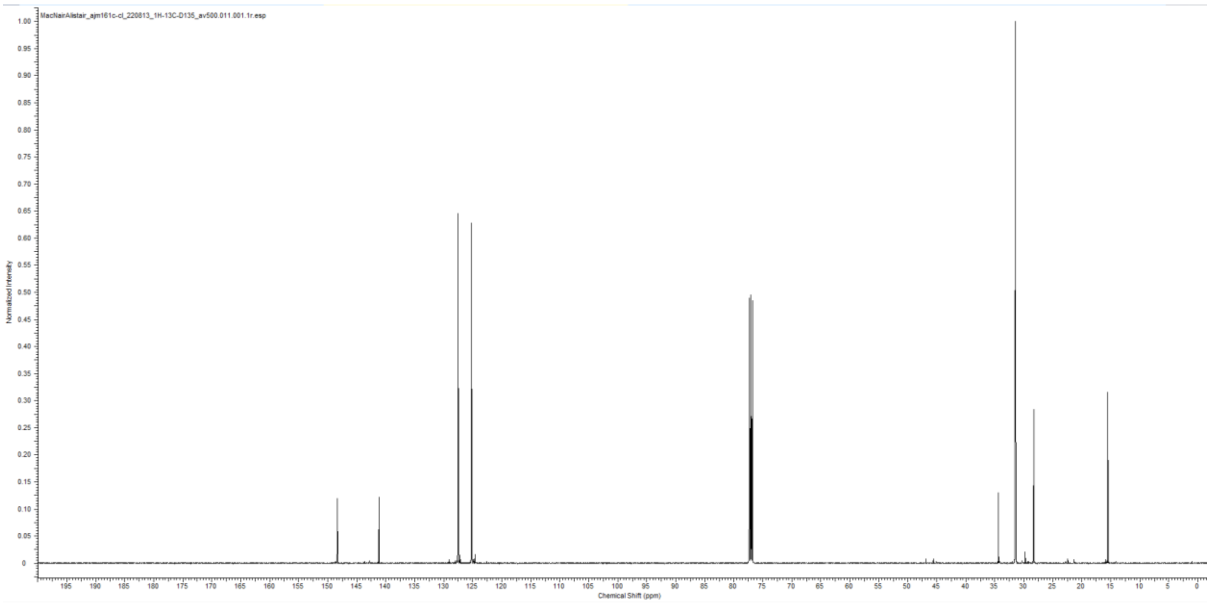

1-Ethyl-4-methoxybenzene (2p)

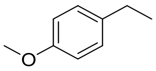

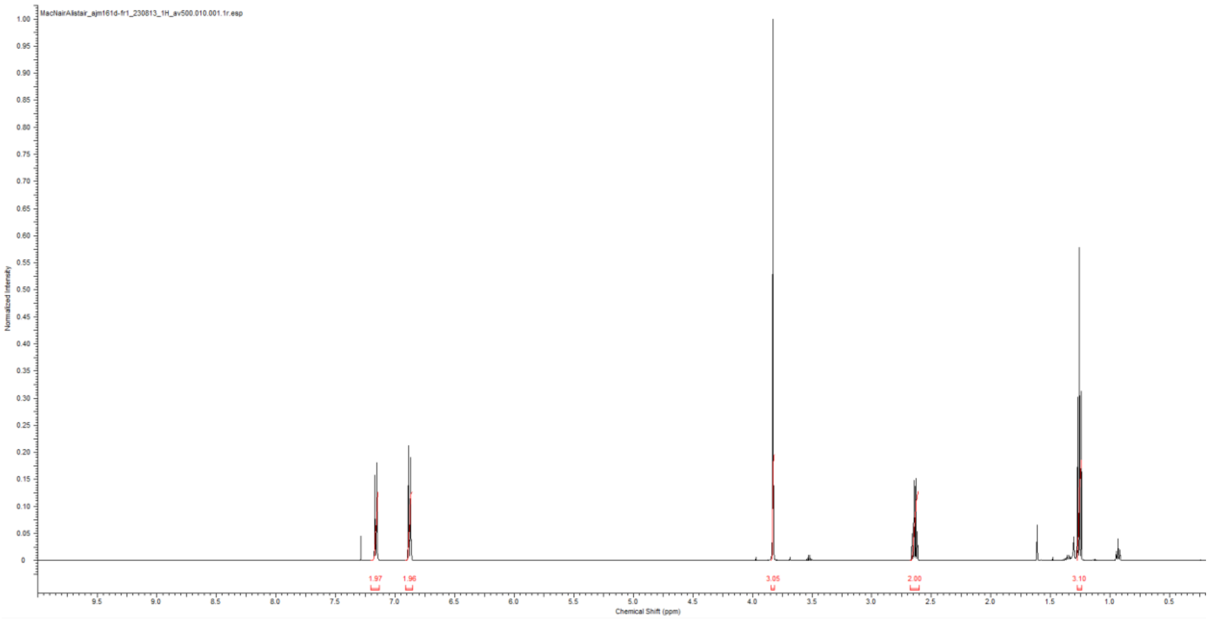

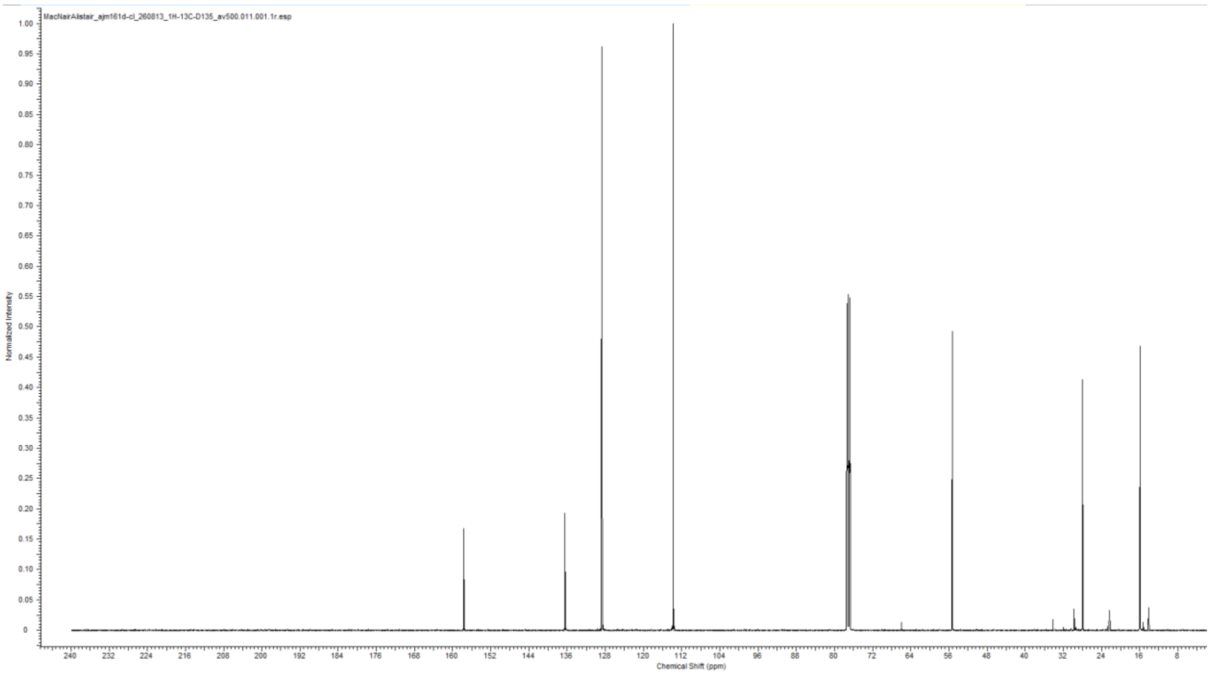

tert-Butyl propionate (2r)

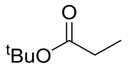

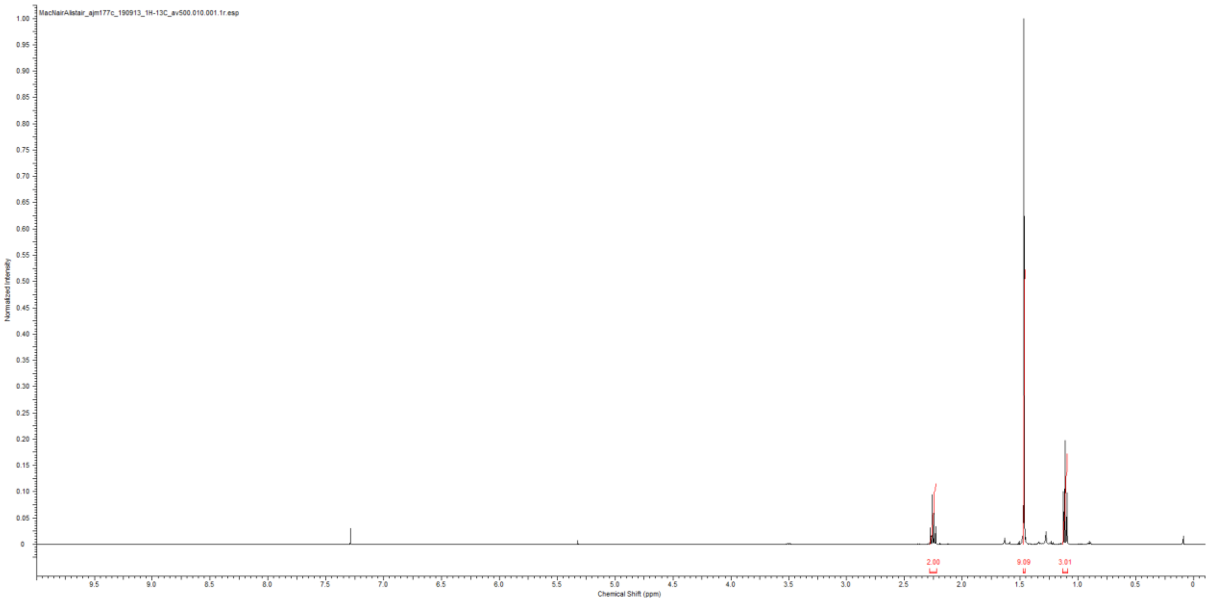

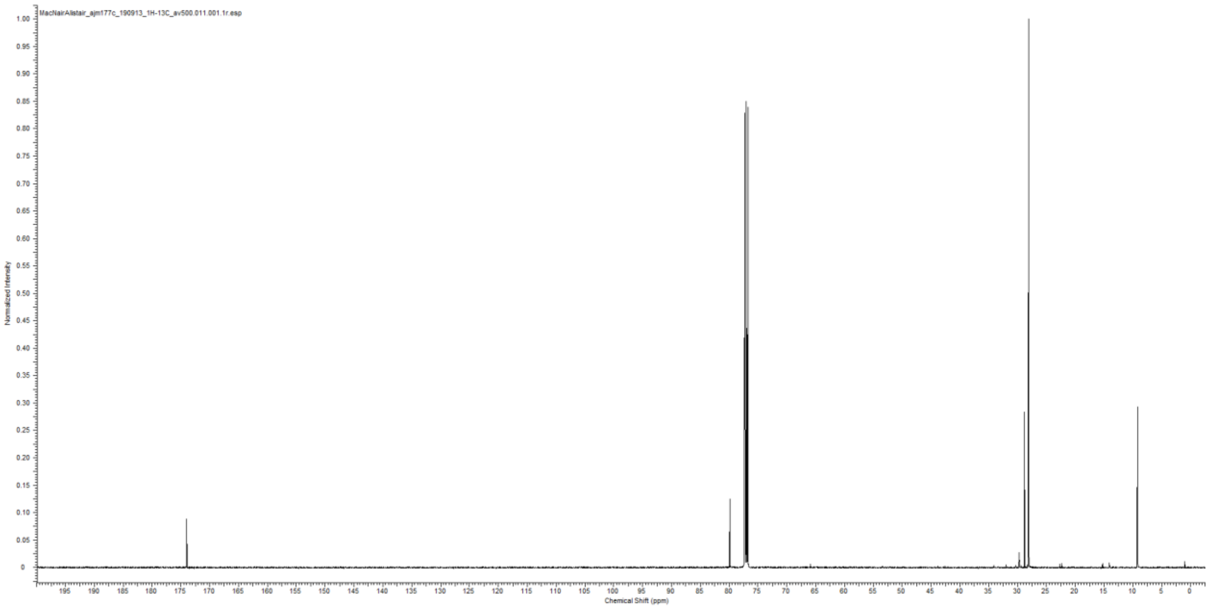

***N*,*N*-Dimethylpropionamide (2s)** (mixture with *N*,*N*-dimethylacrylamide)

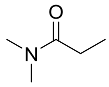

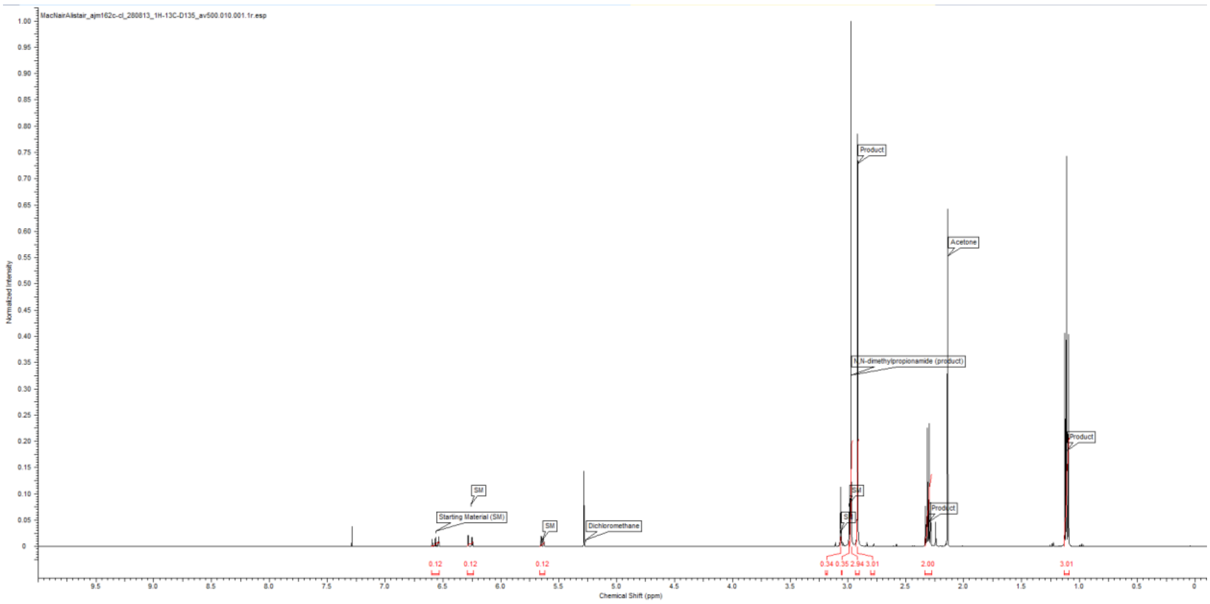

Aniline hydrochloride (6a)

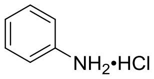

Isolated product: 500 MHz

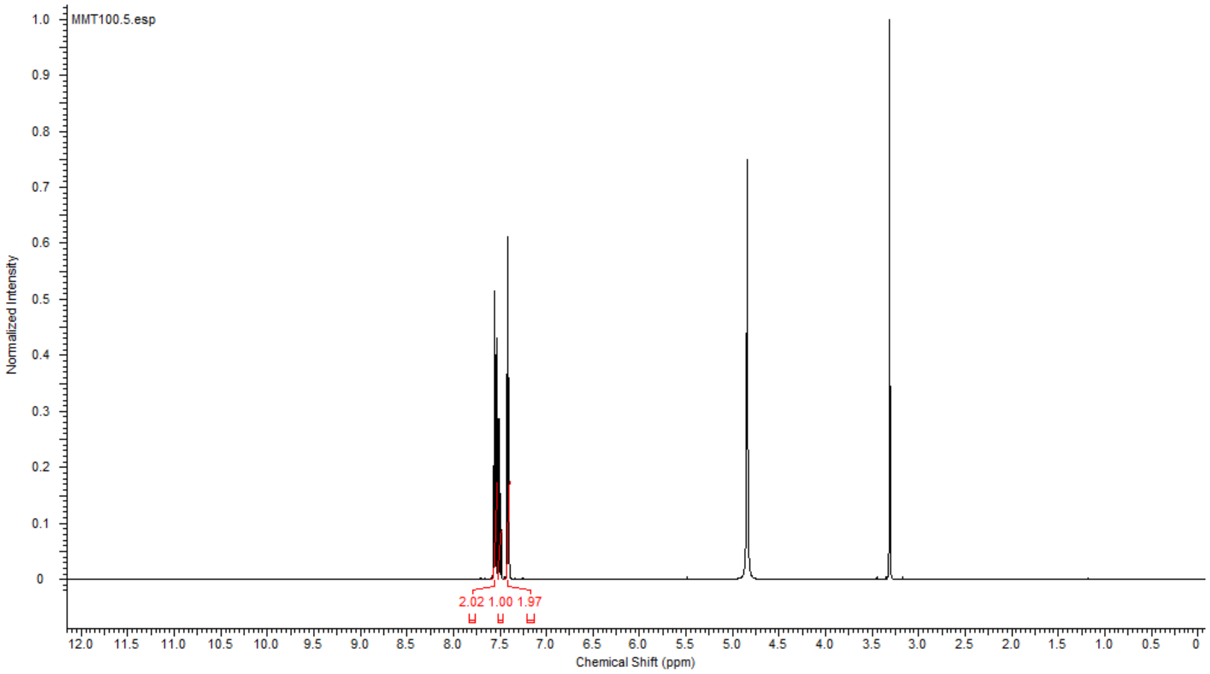

126 MHz

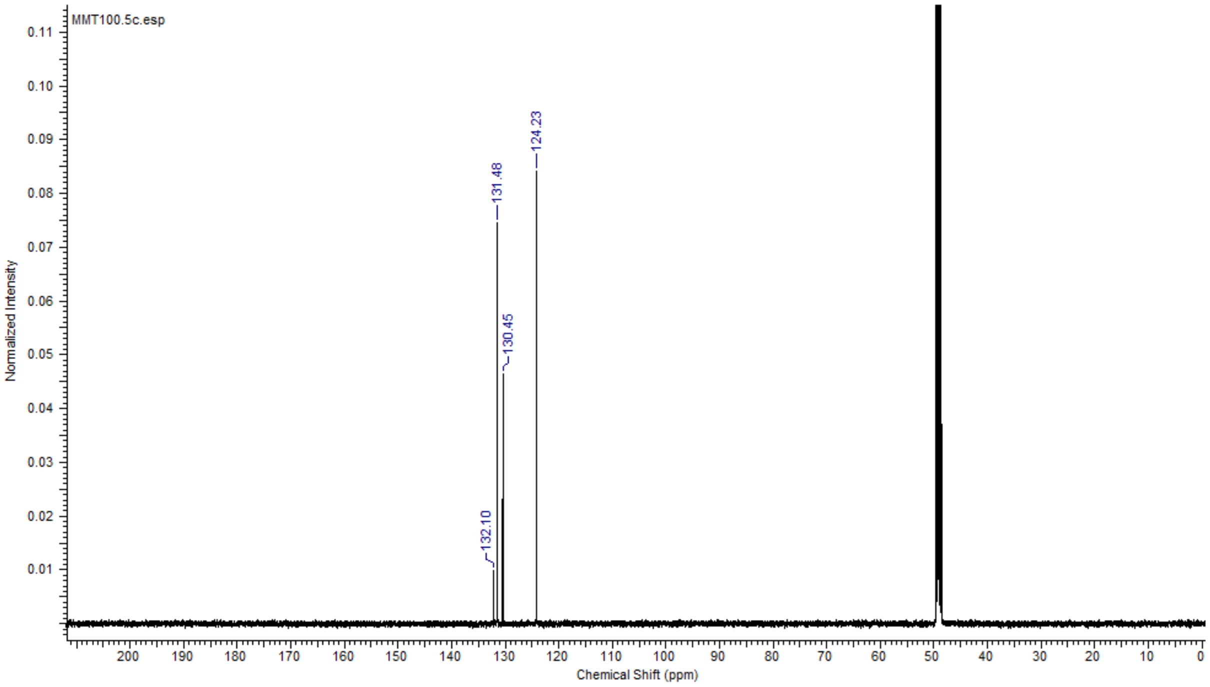

2-Aminotoluene (6b)

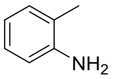

Isolated product: 500 MHz

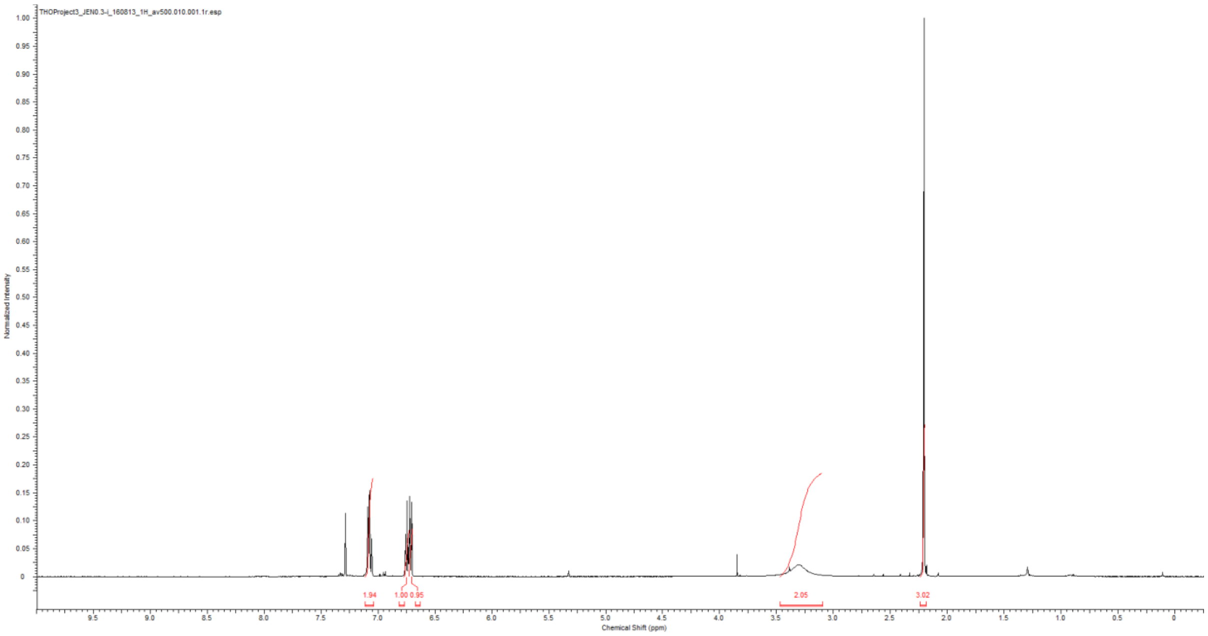

126 MHz

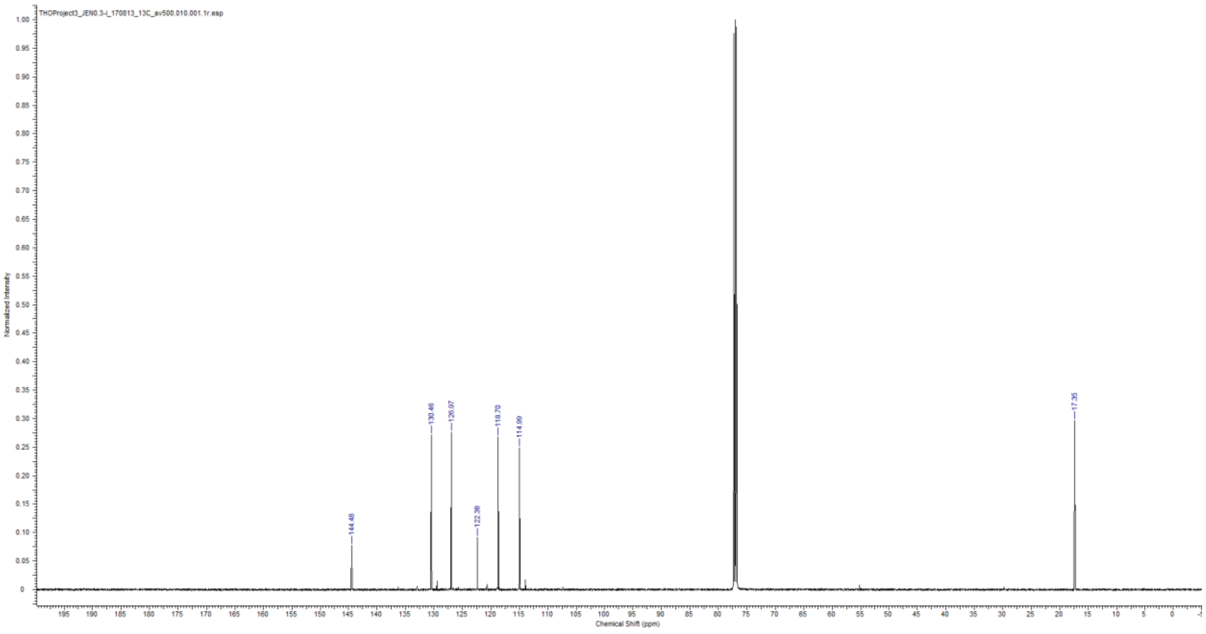

3-Aminotoluene (6c)

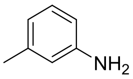

Isolated product: 500 MHz

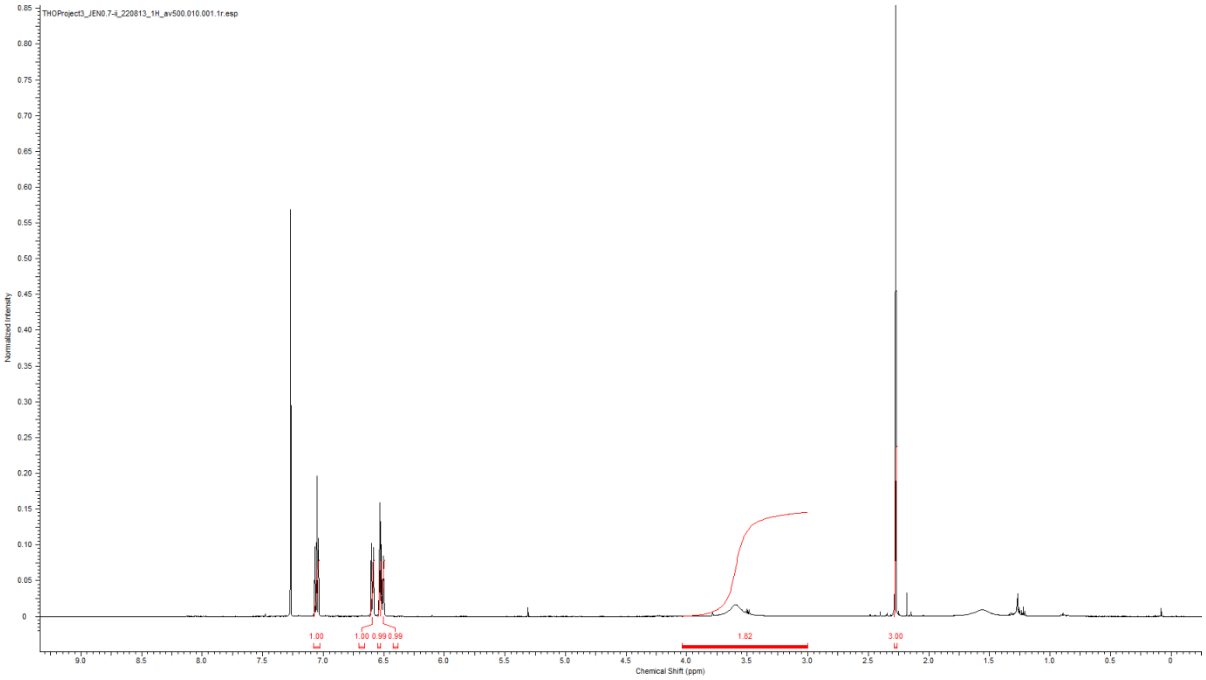

126 MHz

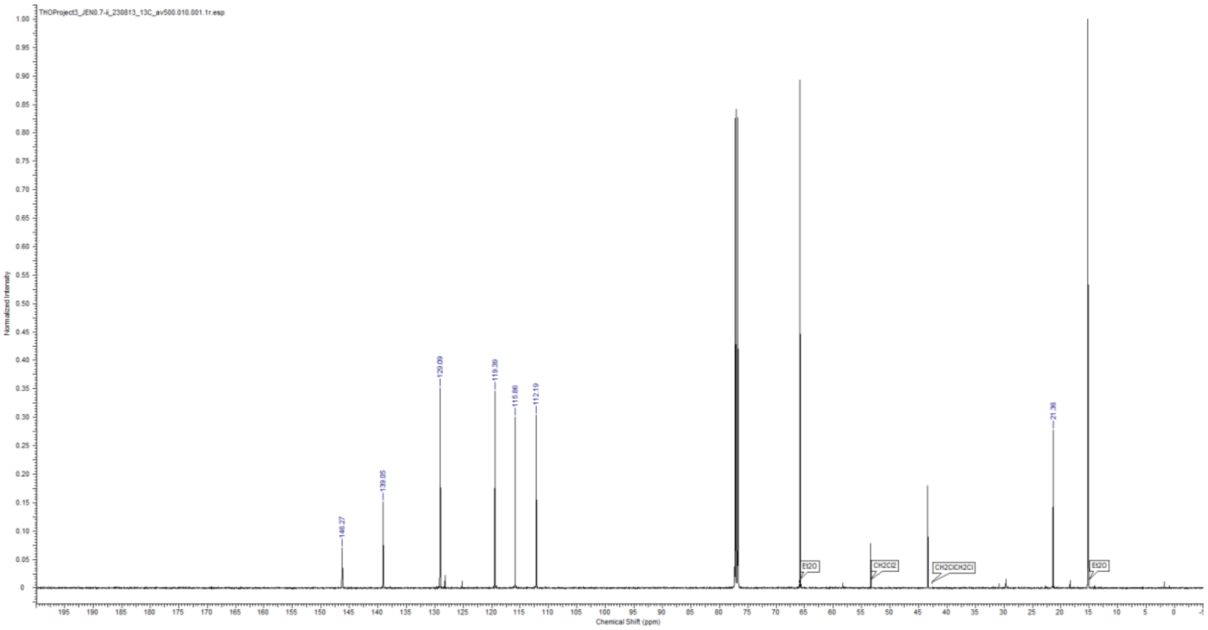

4-Aminotoluene (6d)

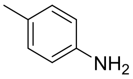

Isolated product: 400 MHz

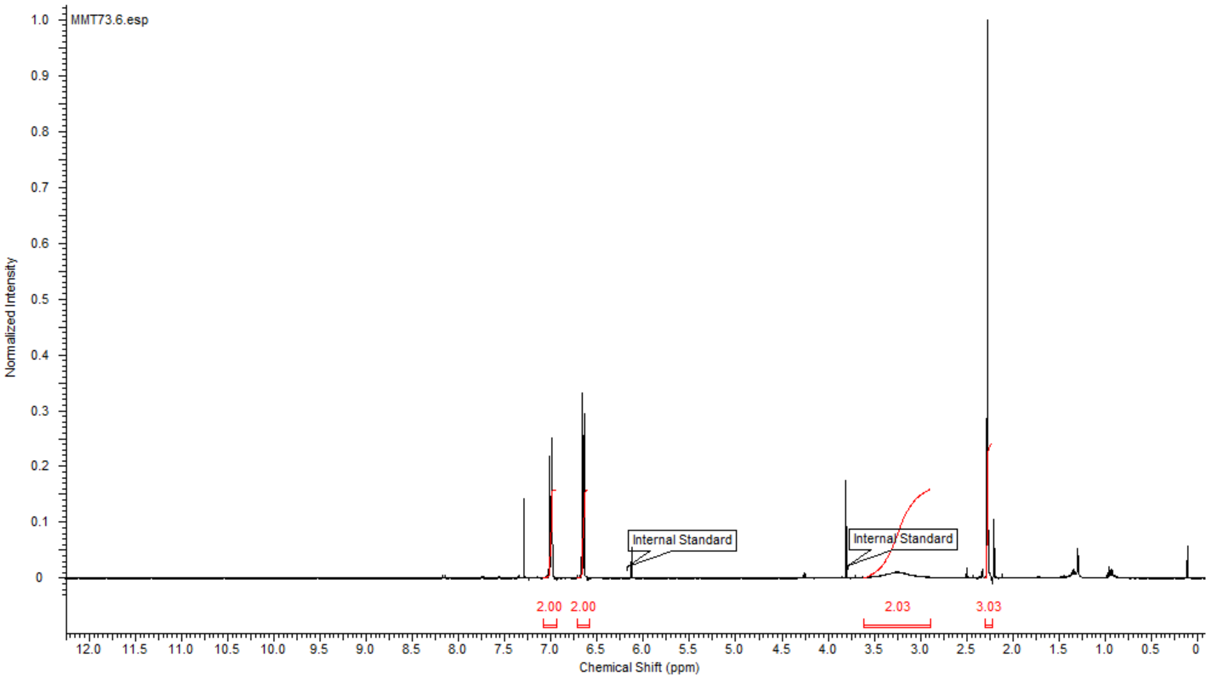

100 MHz

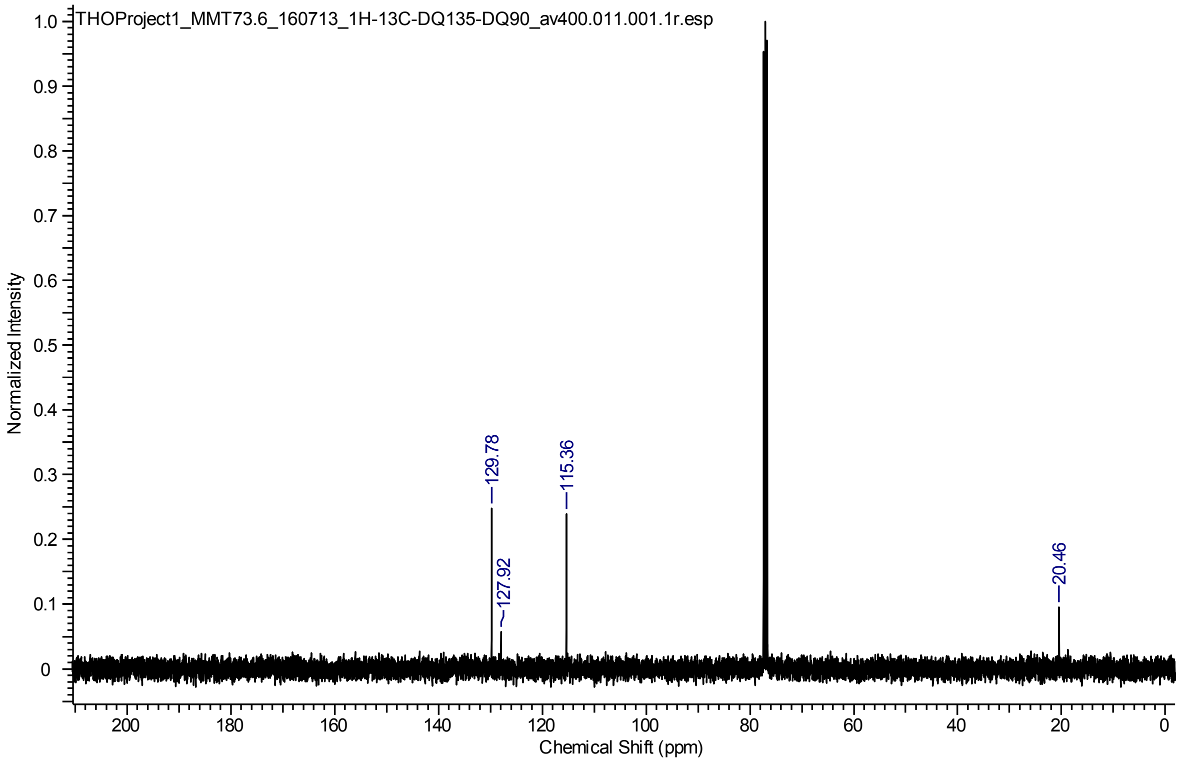

2,6-Dimethylaniline·HCl (6e)

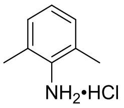

Isolated product: 500 MHz

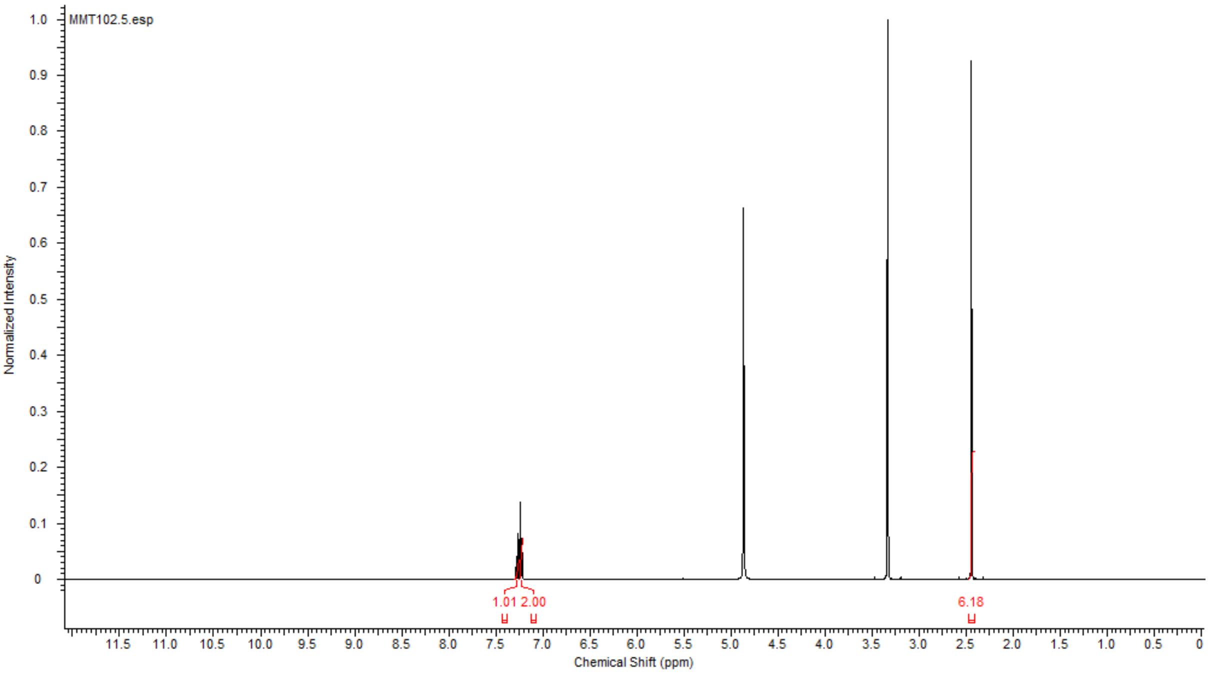

125 MHz

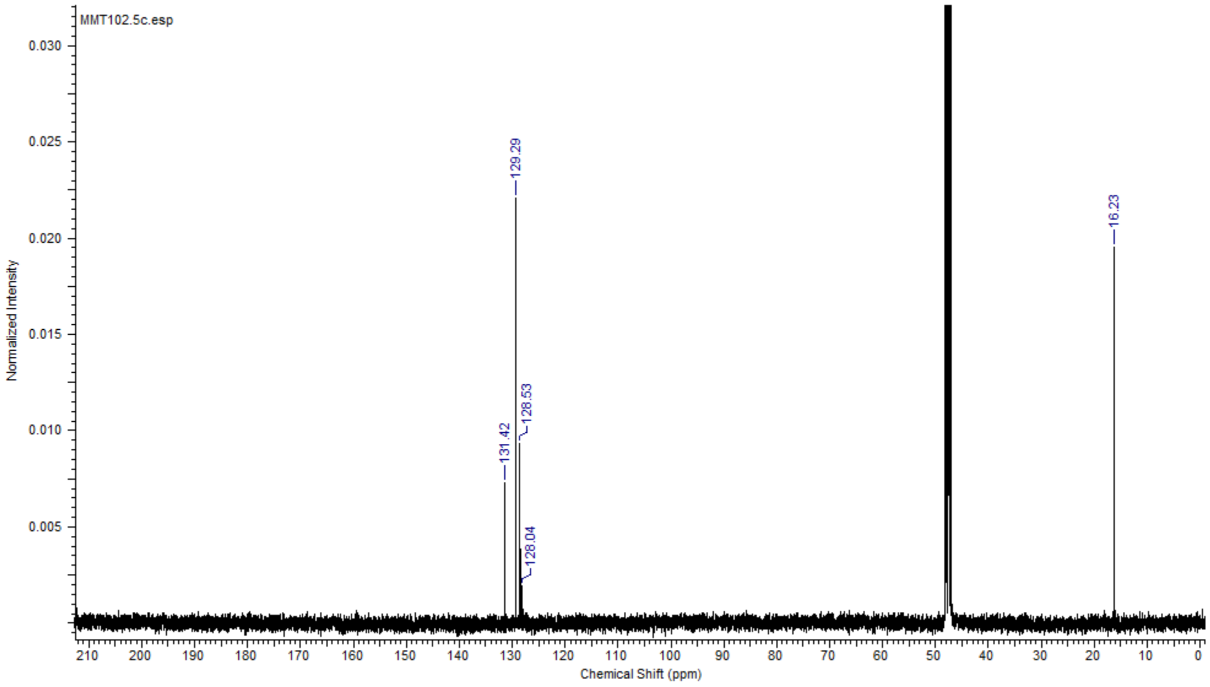

2-Methoxyaniline (6f)

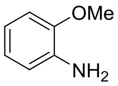

Isolated product: 500 MHz

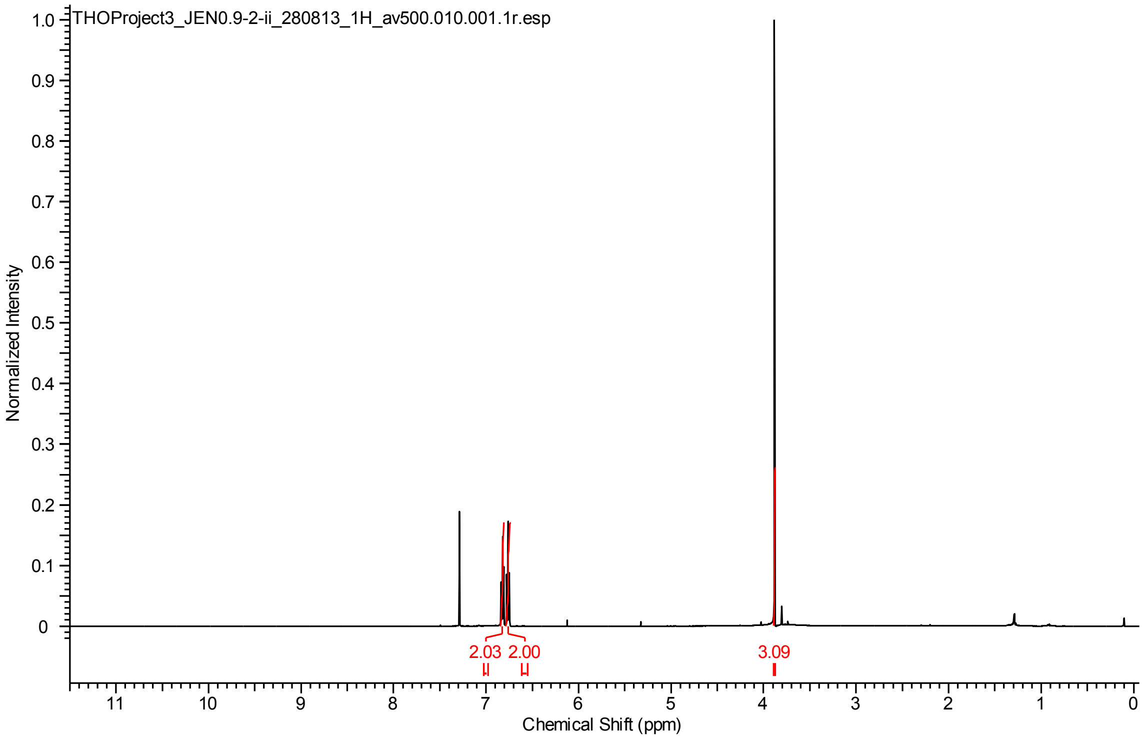

100 MHz

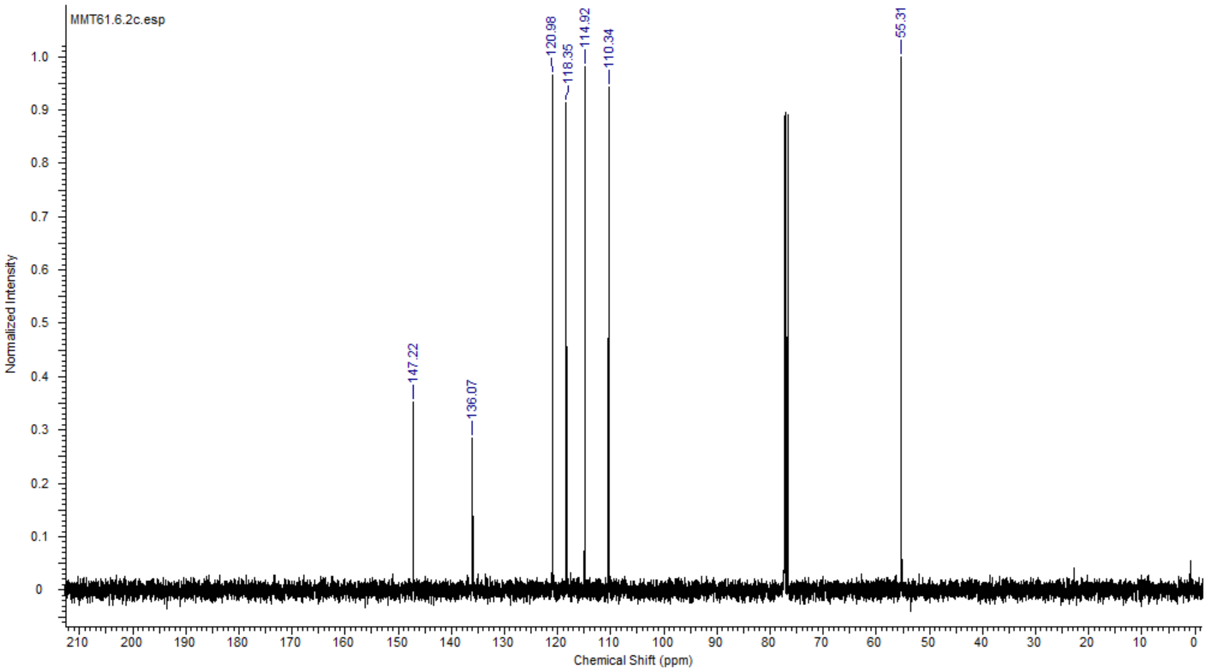

3-Methoxyaniline hydrochloride (6g)

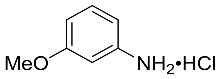

Isolated product: 400 MHz

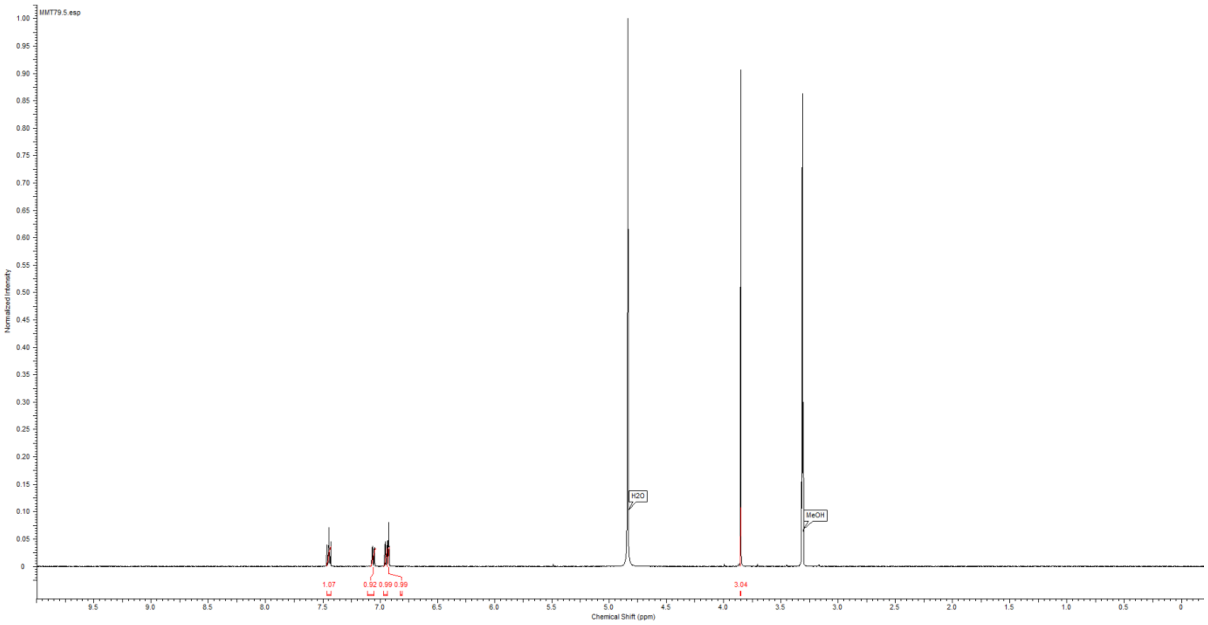

126 MHz

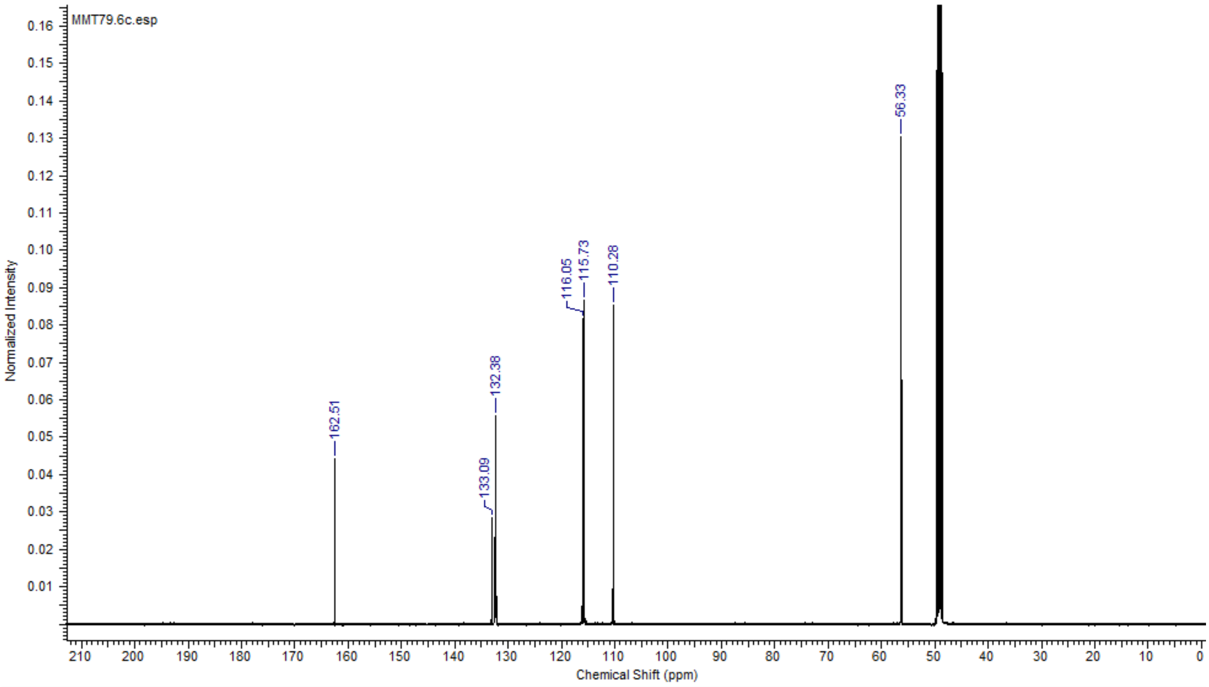

4-Methoxyaniline (6h)

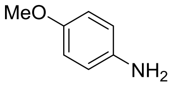

Isolated product: 400 MHz

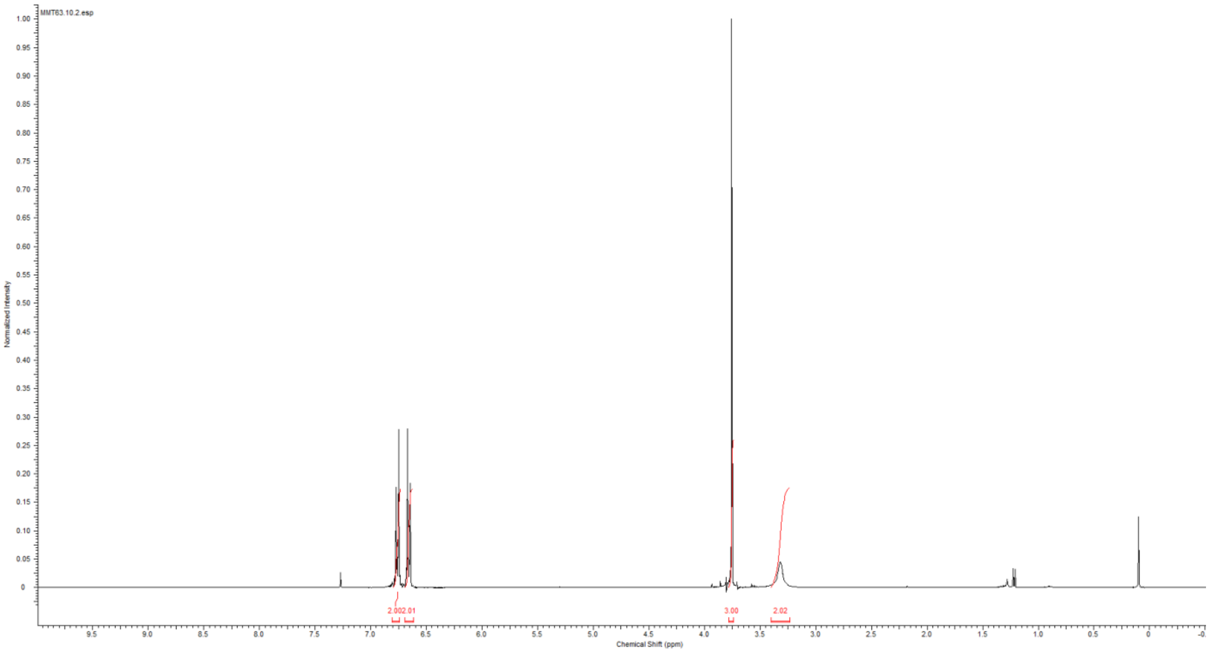

100 MHz

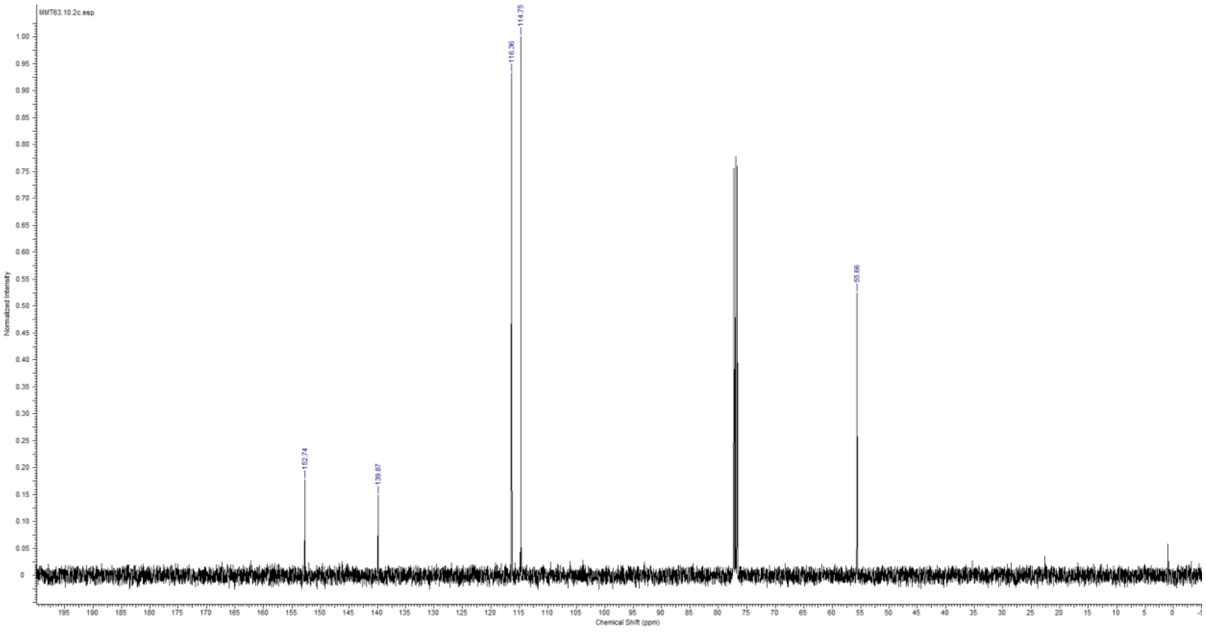

3-(Trifluoromethyl)aniline (6i)

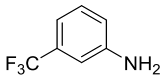

Isolated product: 500 MHz

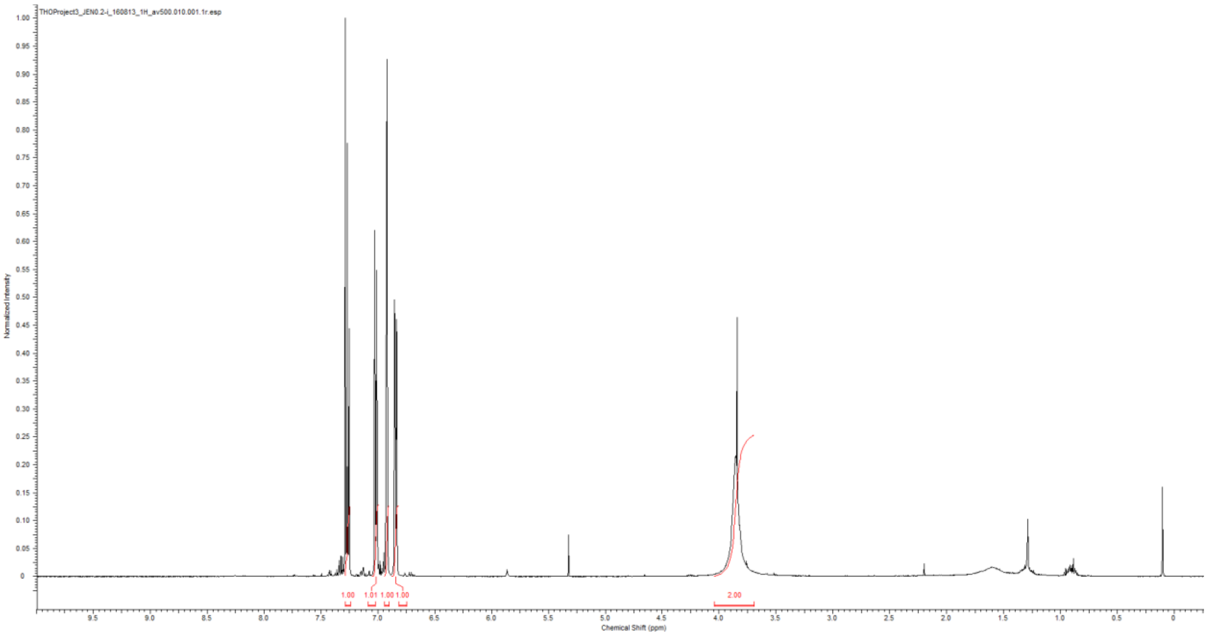

126 MHz

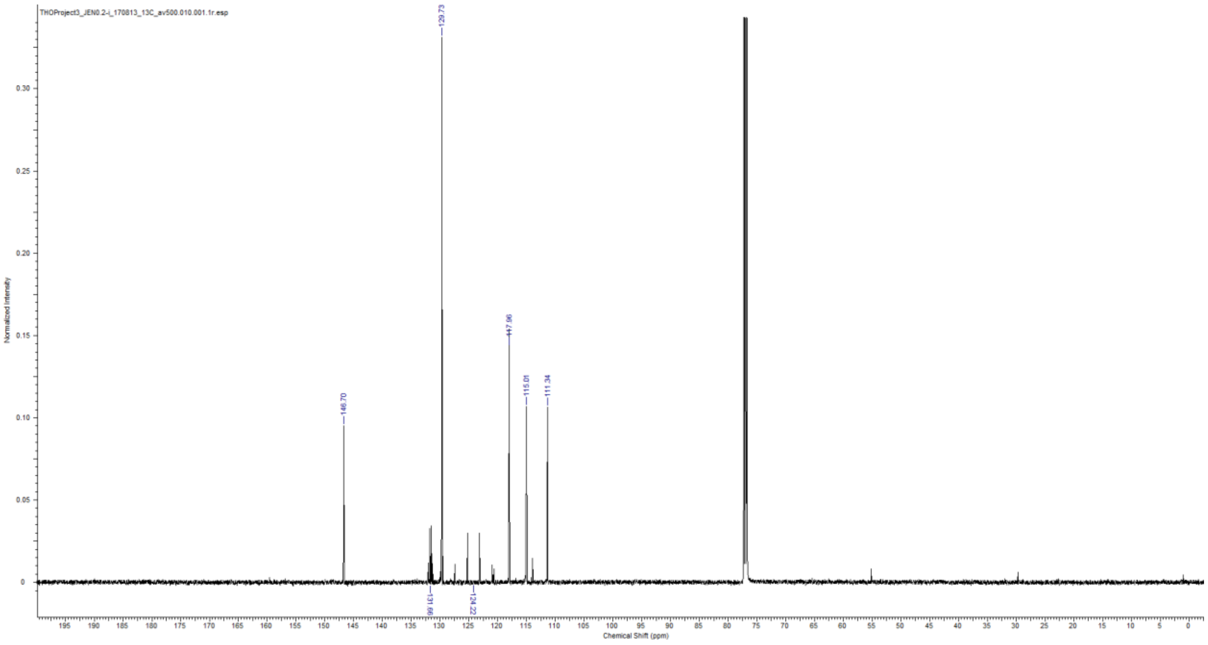

4-(Trifluoromethyl)aniline hydrochloride (6j)

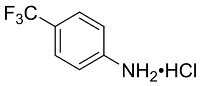

Isolated product: 500 MHz

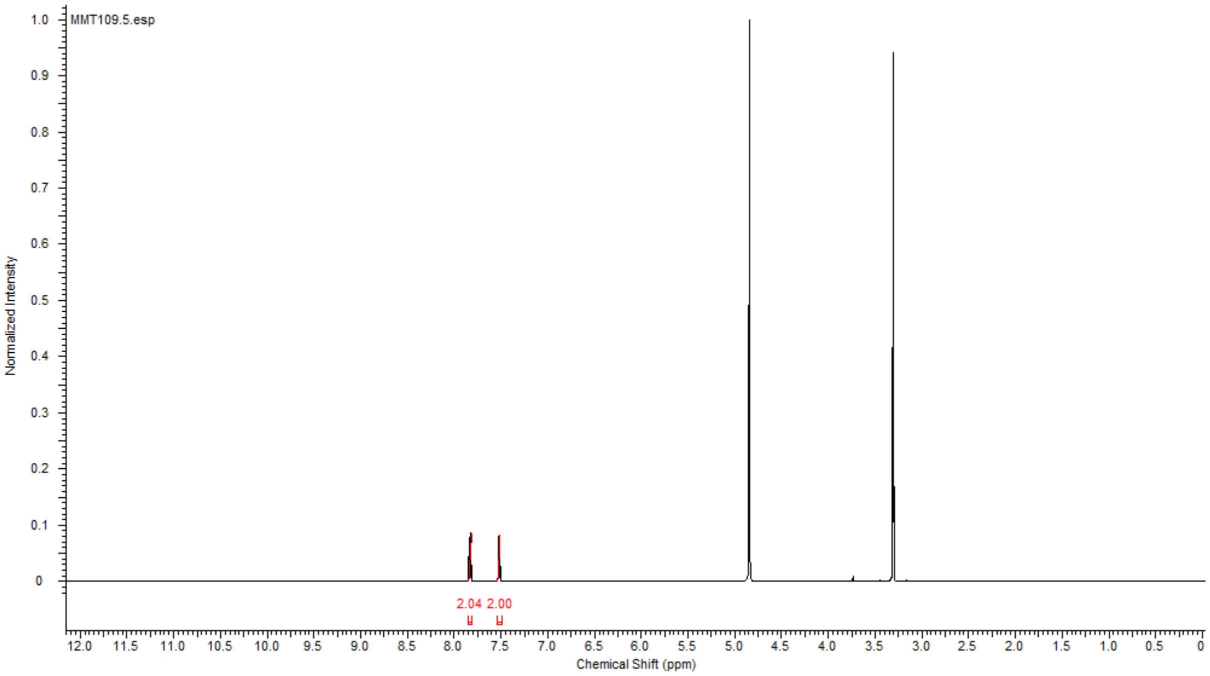

2-Fluoroaniline (6k)

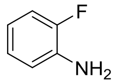

Crude reaction mixture: 400 MHz

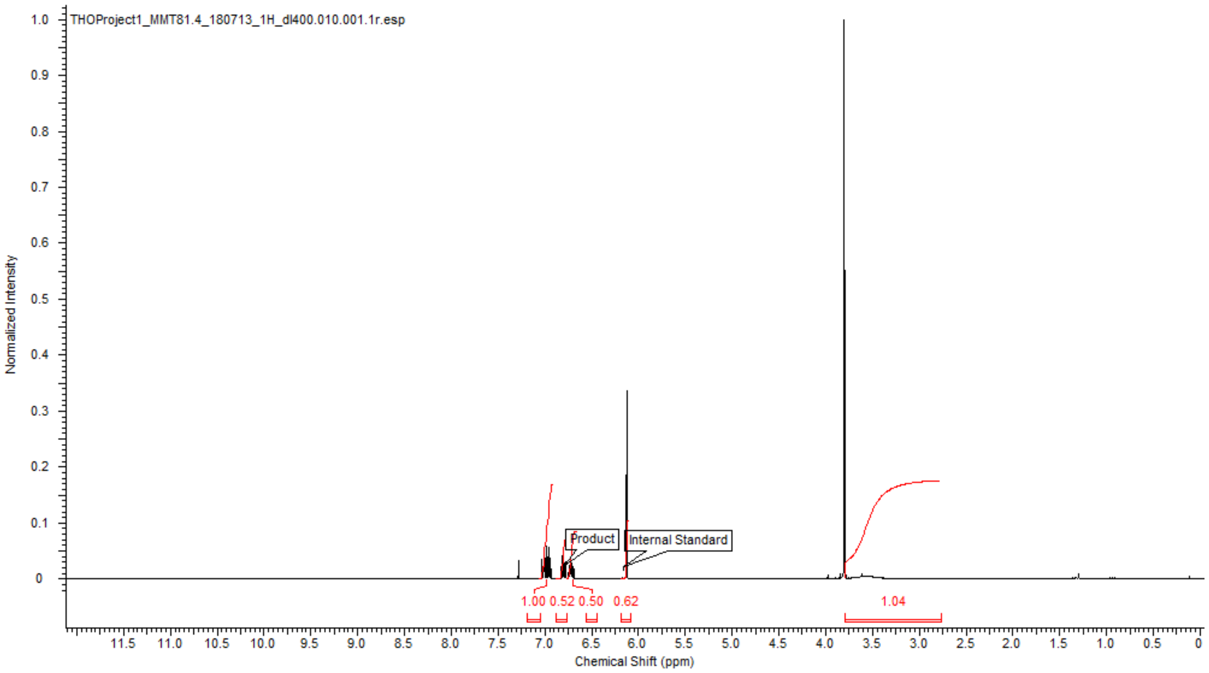

4-Fluoroaniline (6l)

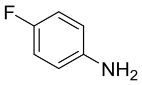

Crude reaction mixture: 400 MHz

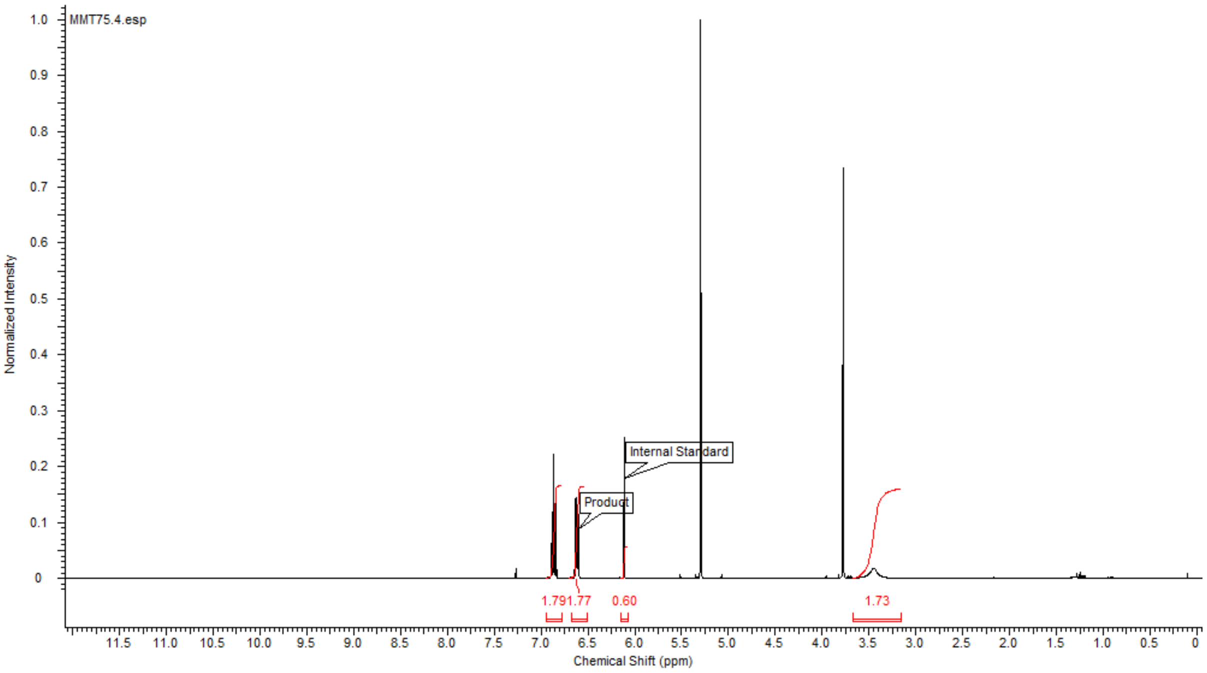

2-Chloroaniline (6m)

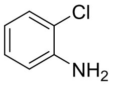

Isolated product: 500 MHz

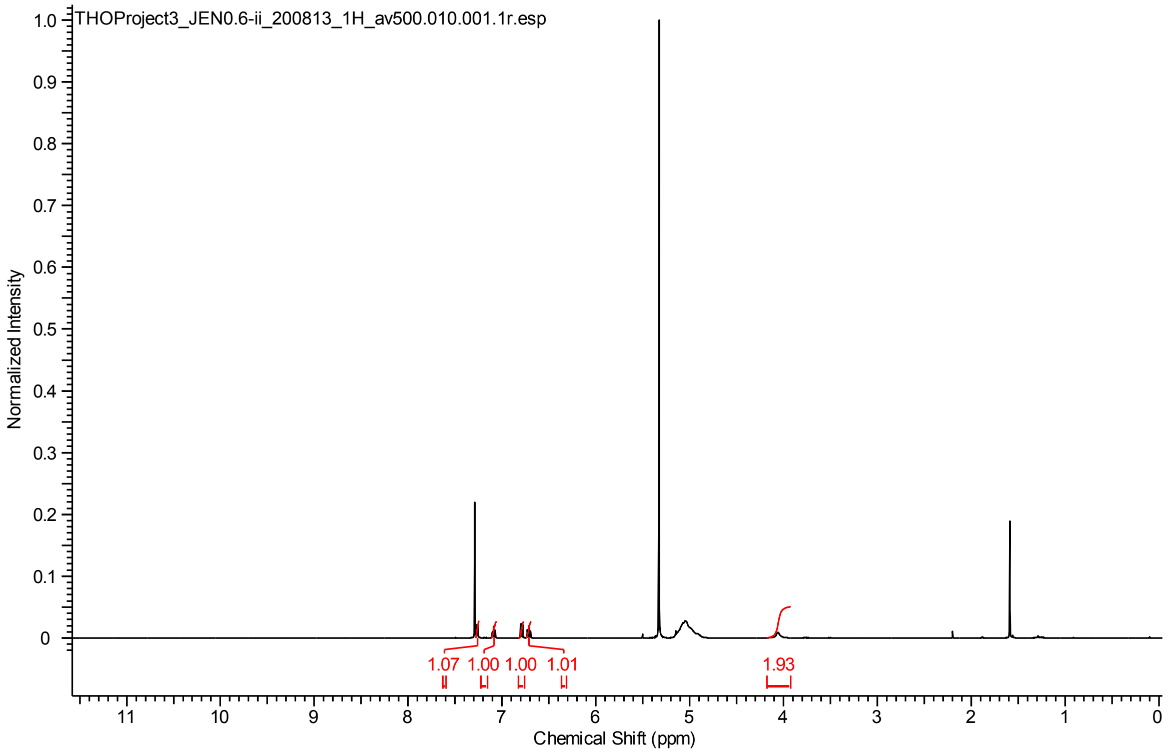

126 MHz

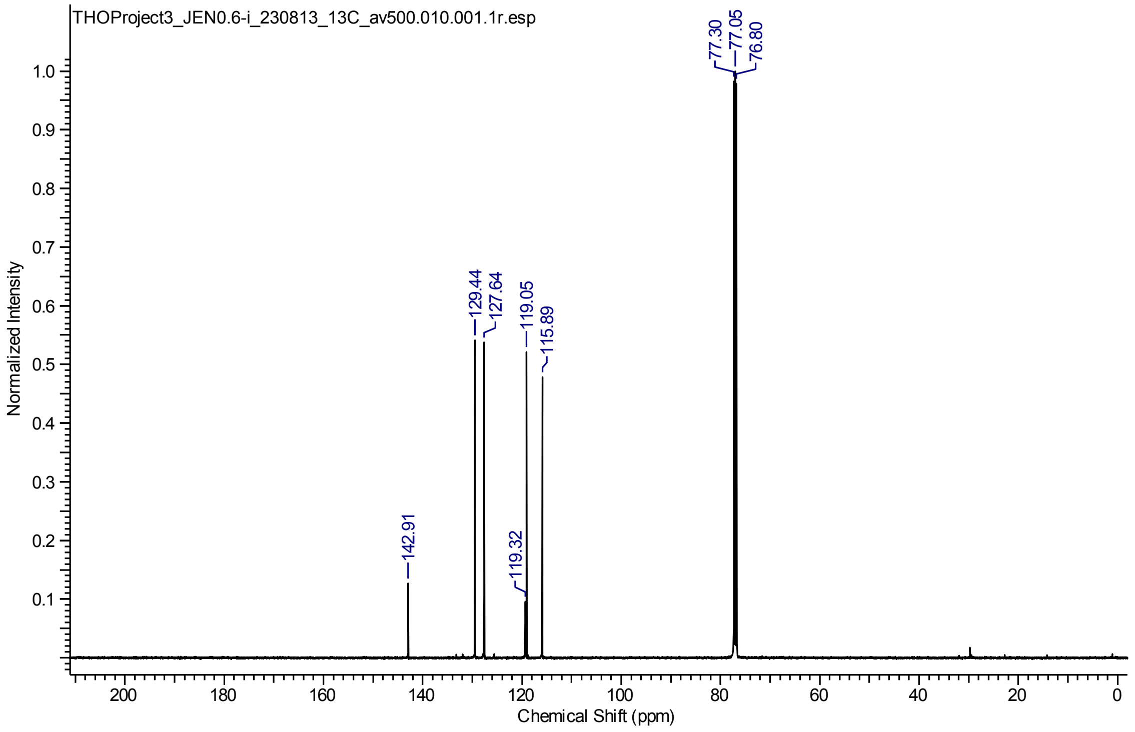

4-Chloroaniline (6n)

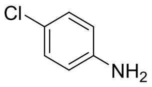

Isolated product: 500 MHz

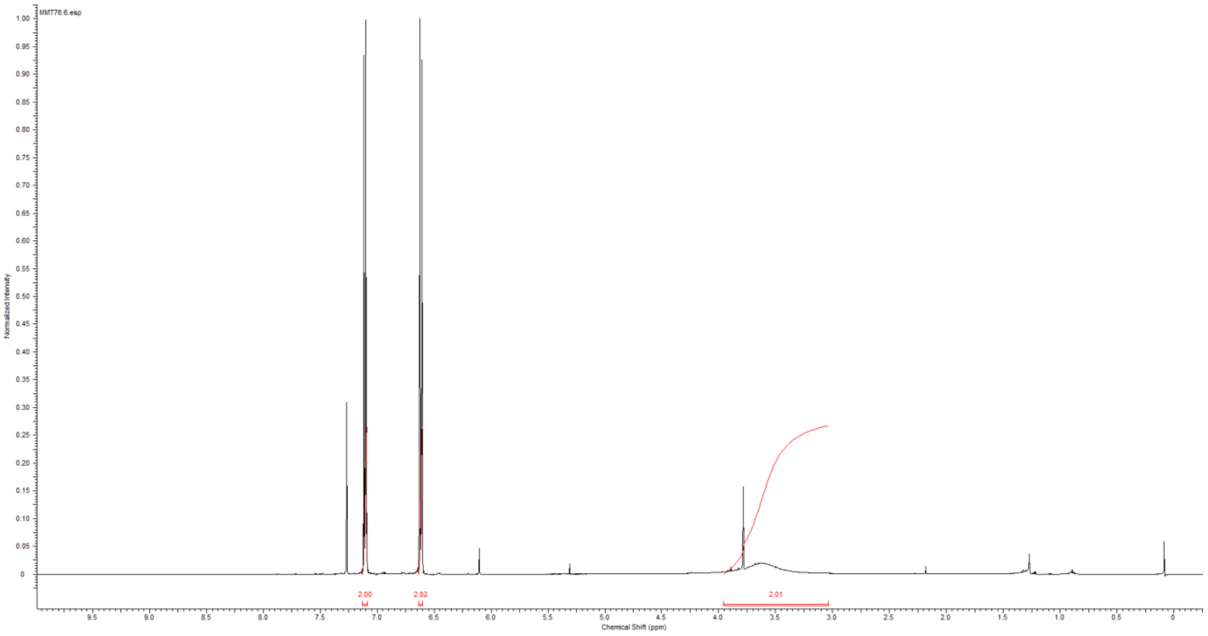

126 MHz

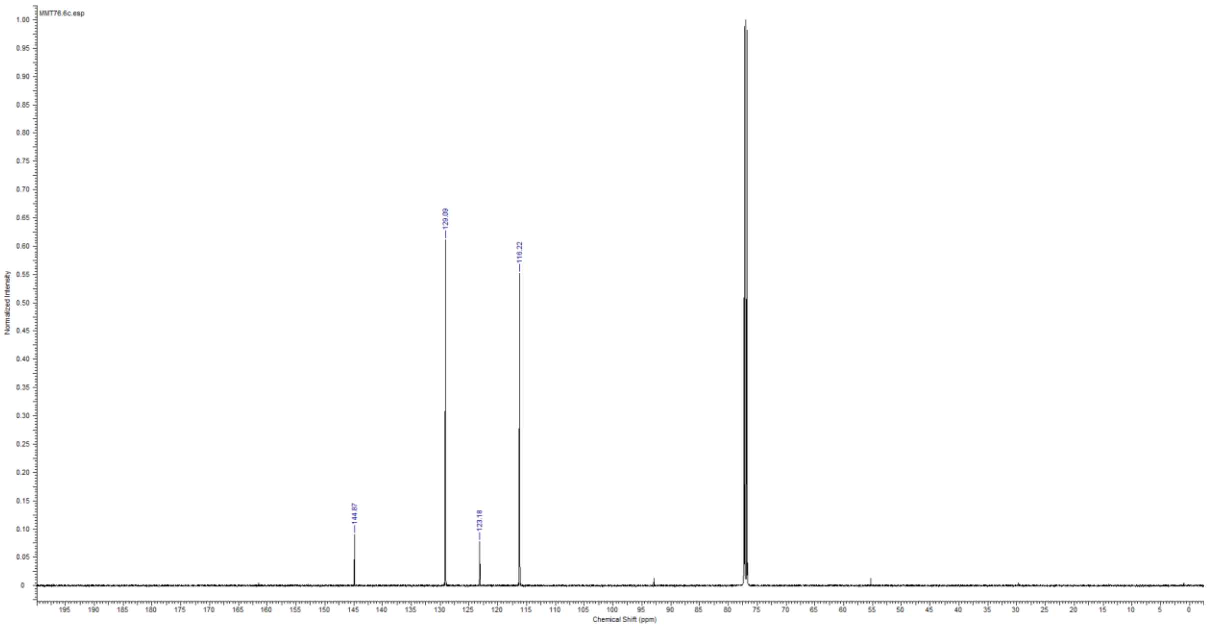

4-Bromoaniline (6o)

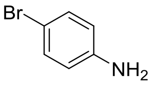

Isolated product: 600 MHz

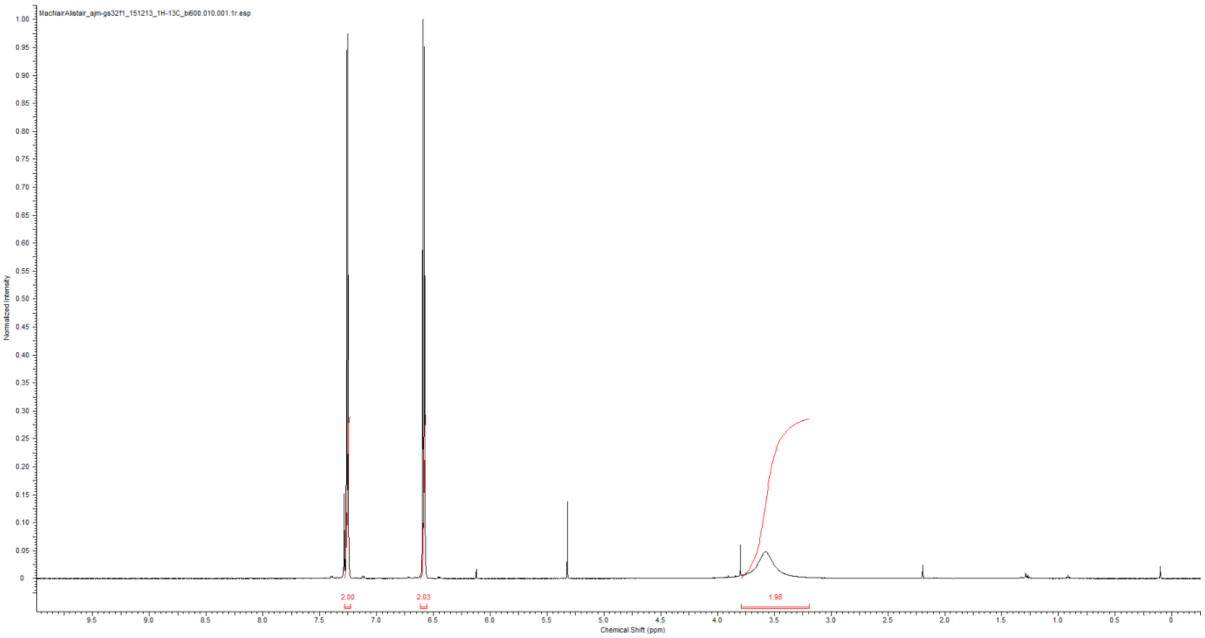

150 MHz

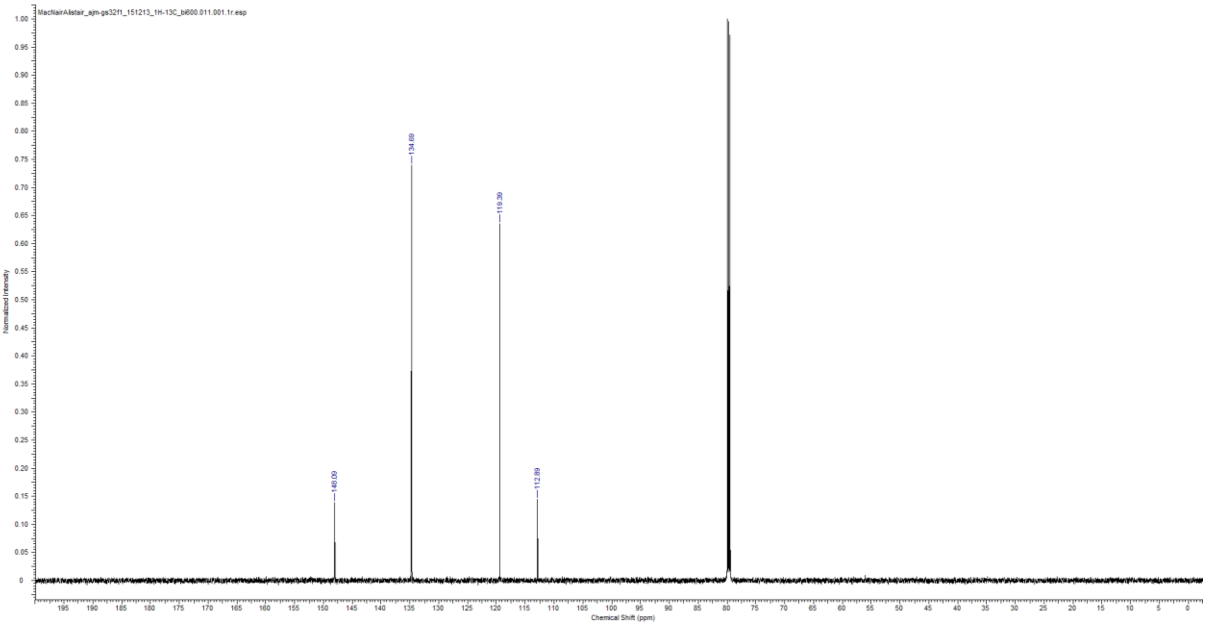

1-(4-Aminophenyl)ethanol hydrochloride (6p)

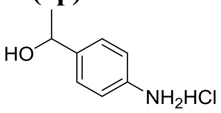

Isolated product: 400 MHz

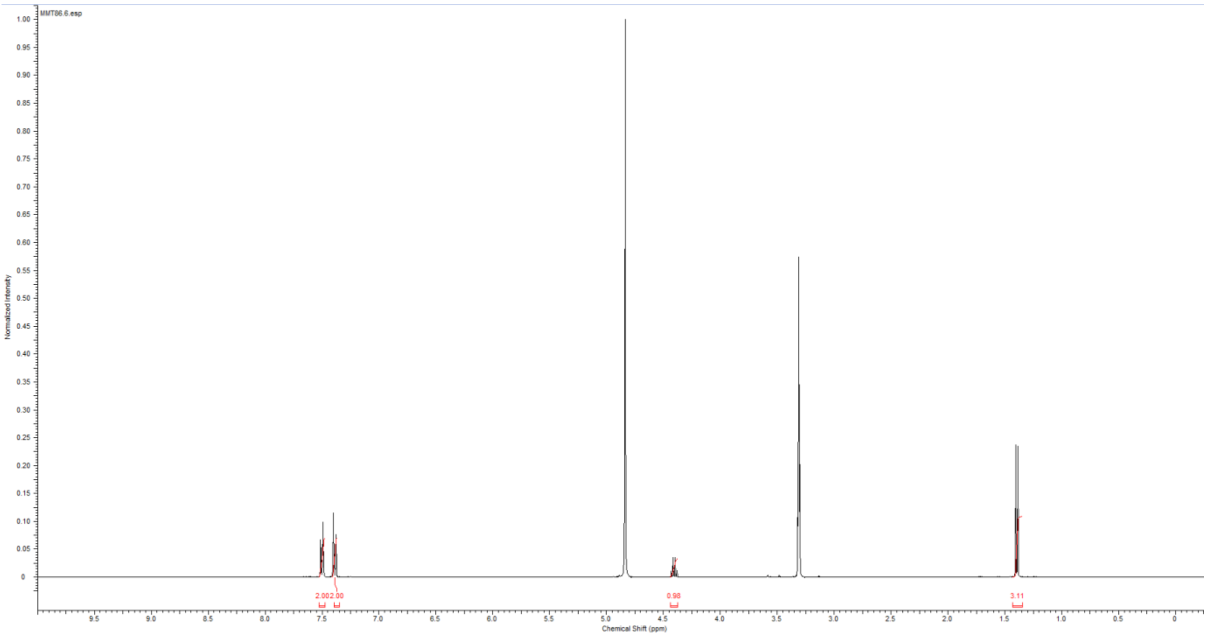

126 MHz

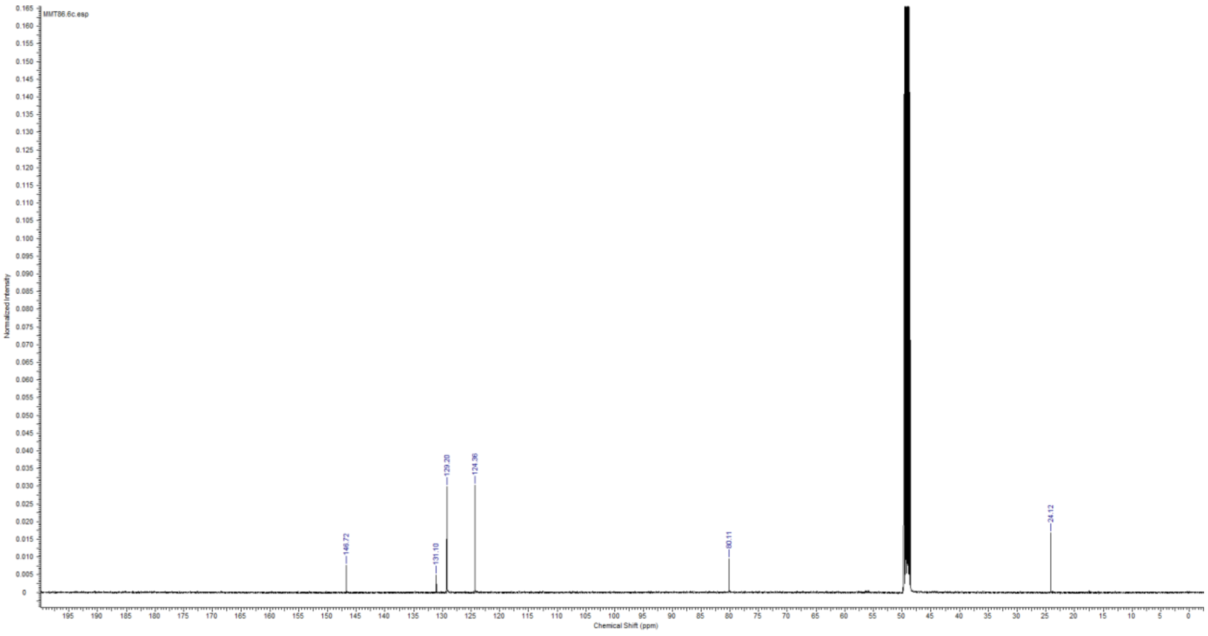

Methyl 4-aminobenzoate (6q)

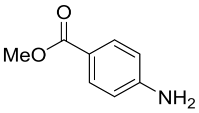

,

Isolated product: 600 MHz

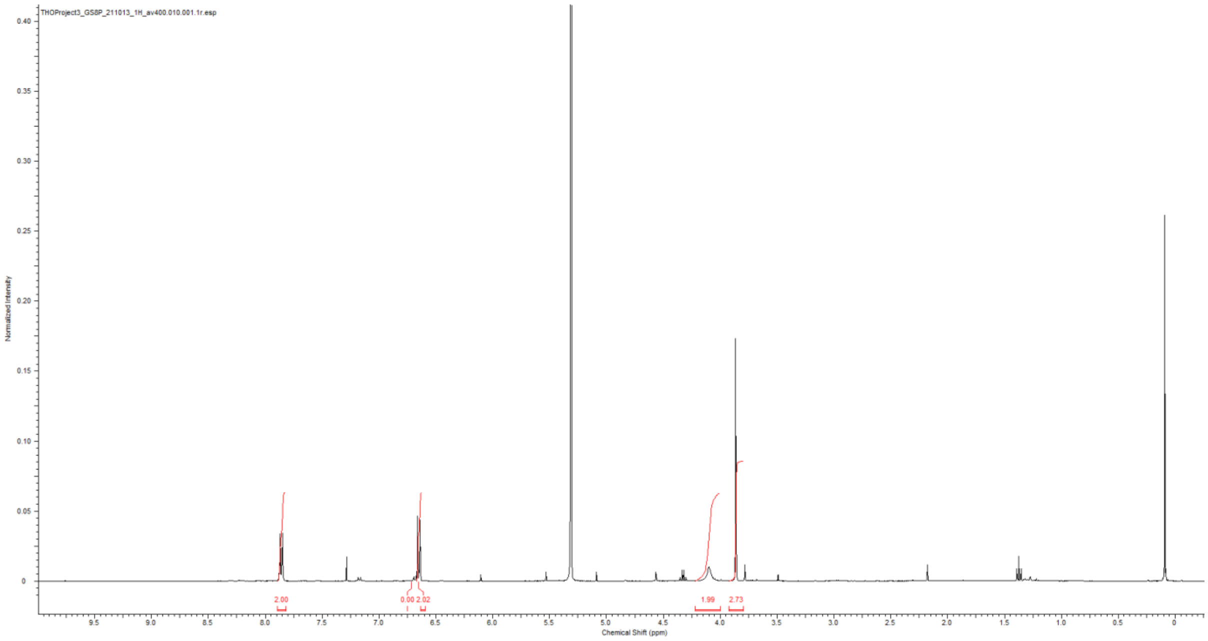

150 MHz

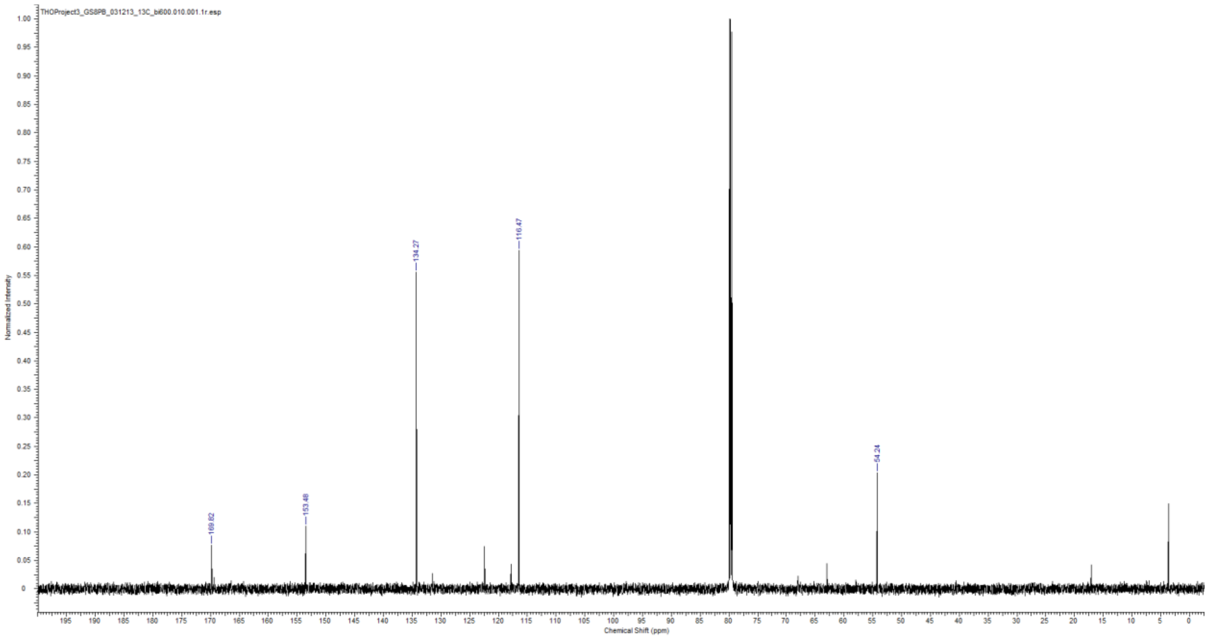

Ethyl 4-aminobenzoate (6r)

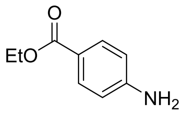

Isolated product: 600 MHz

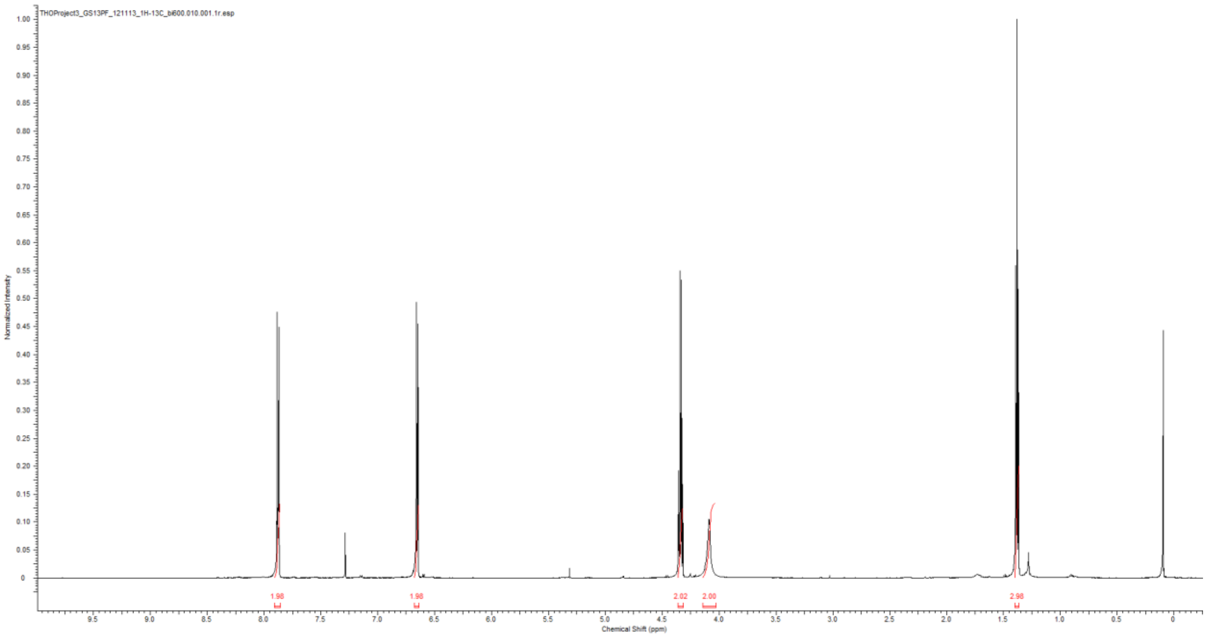

150 MHz

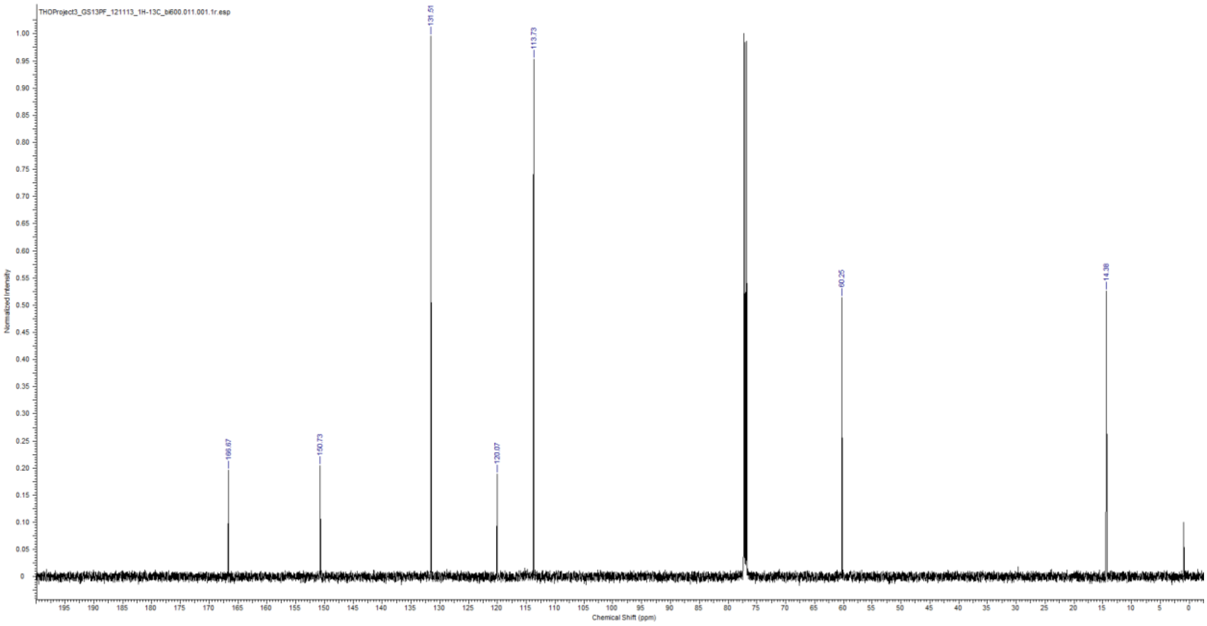

4-(4-Aminobenzoyl)morpholine (6s)

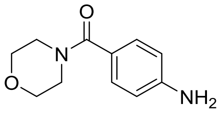

Isolated product: 600 MHz

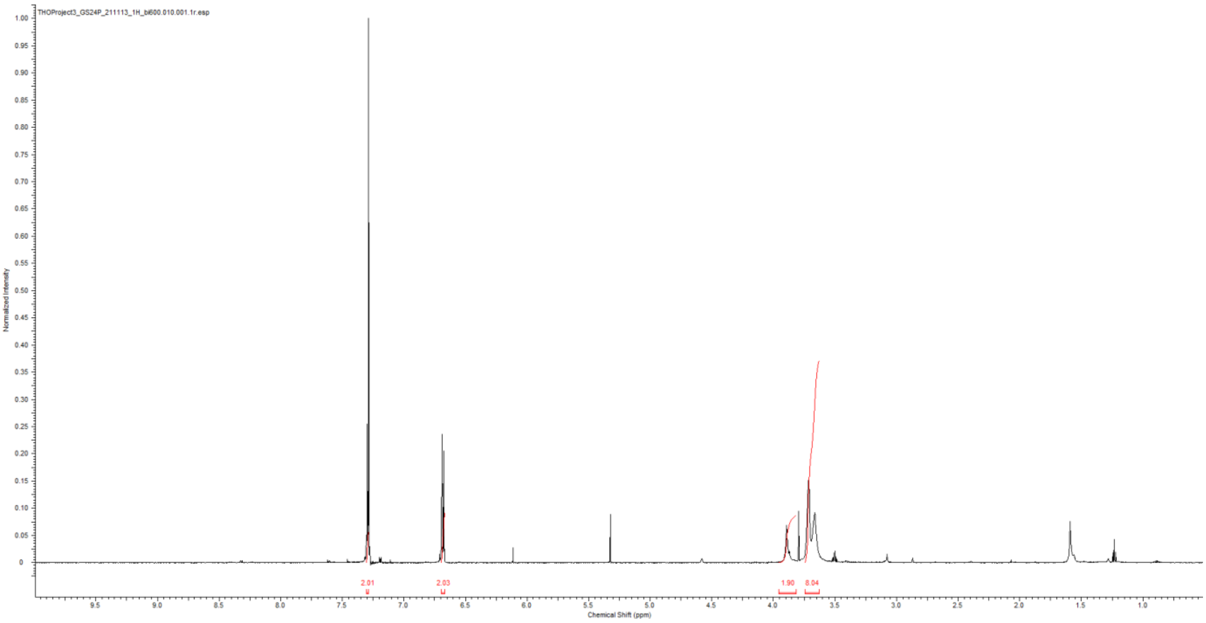

150 MHz

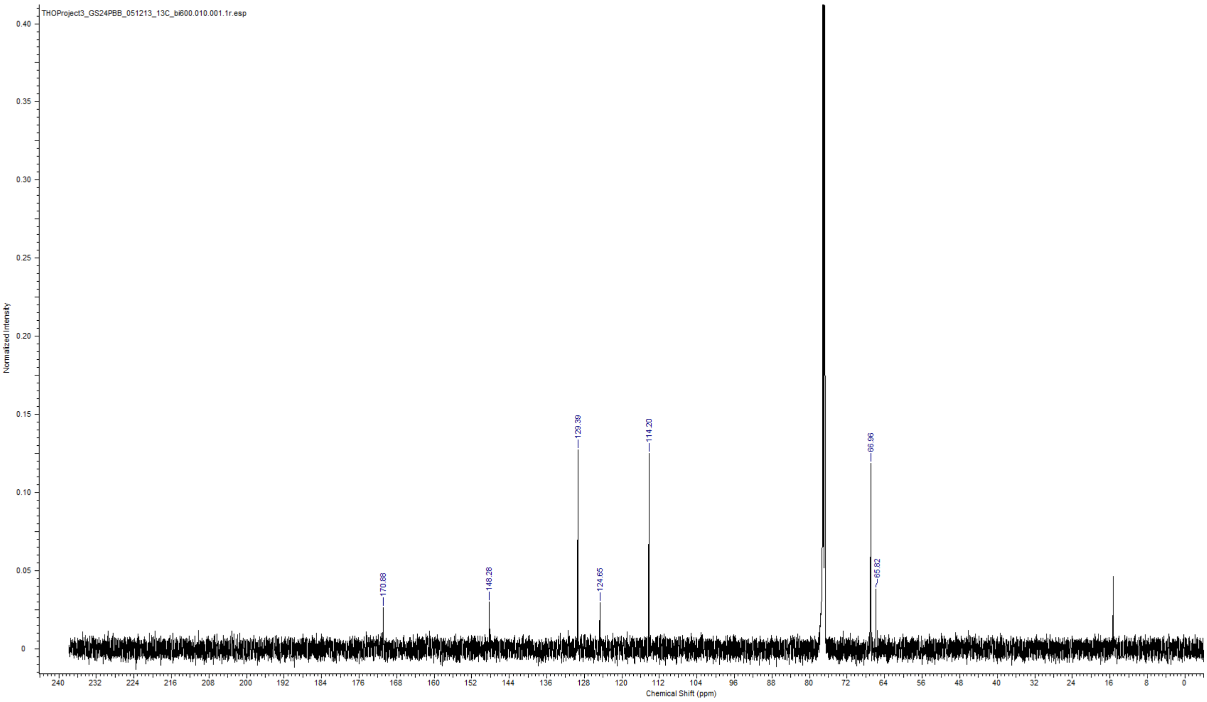

4-(Methylthio)-aniline (6t)

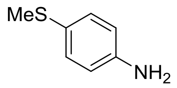

Isolated product: 500 MHz

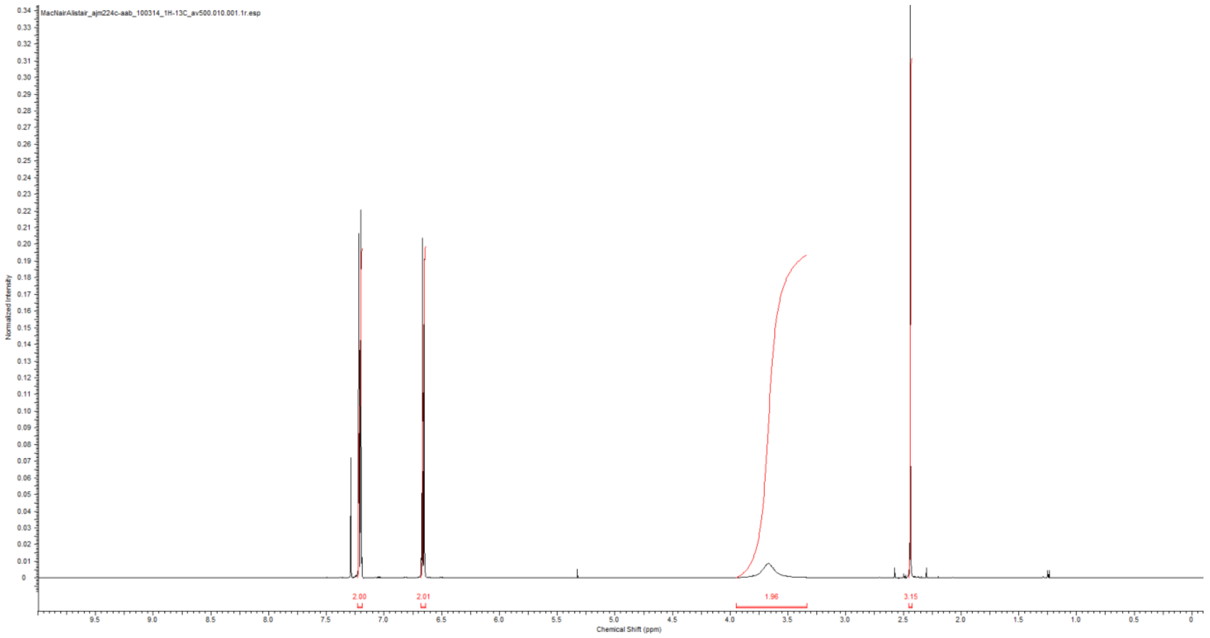

126 MHz

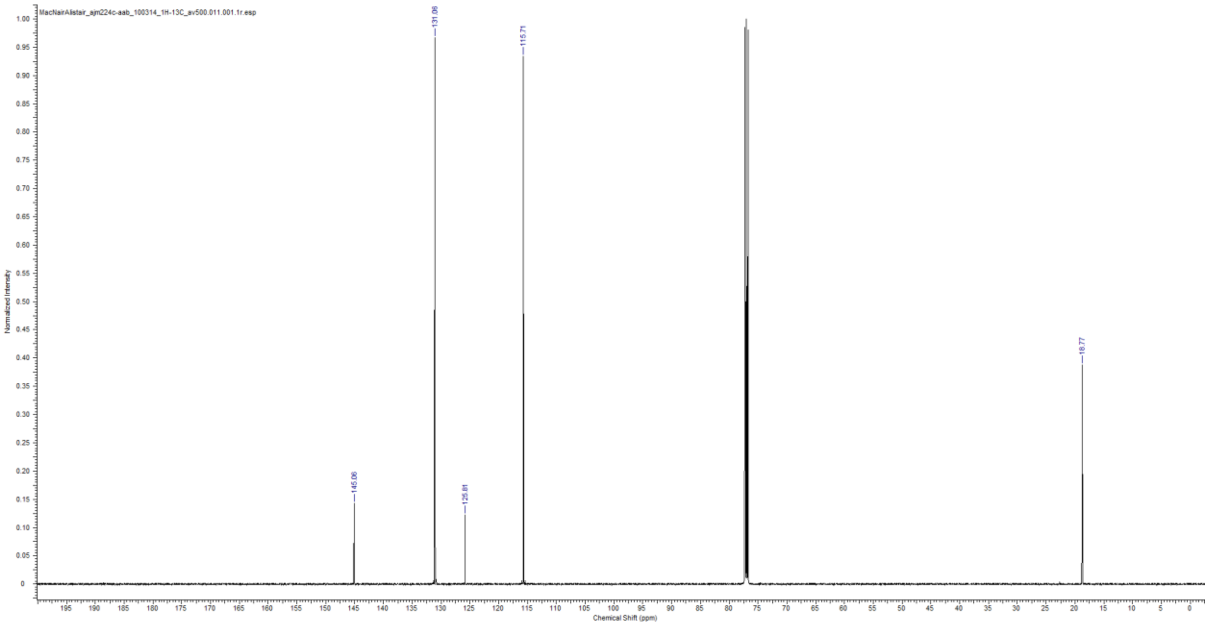

4-(Methylsulfonyl)-aniline (6u)

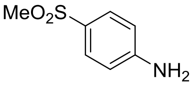

Isolated product: 500 MHz

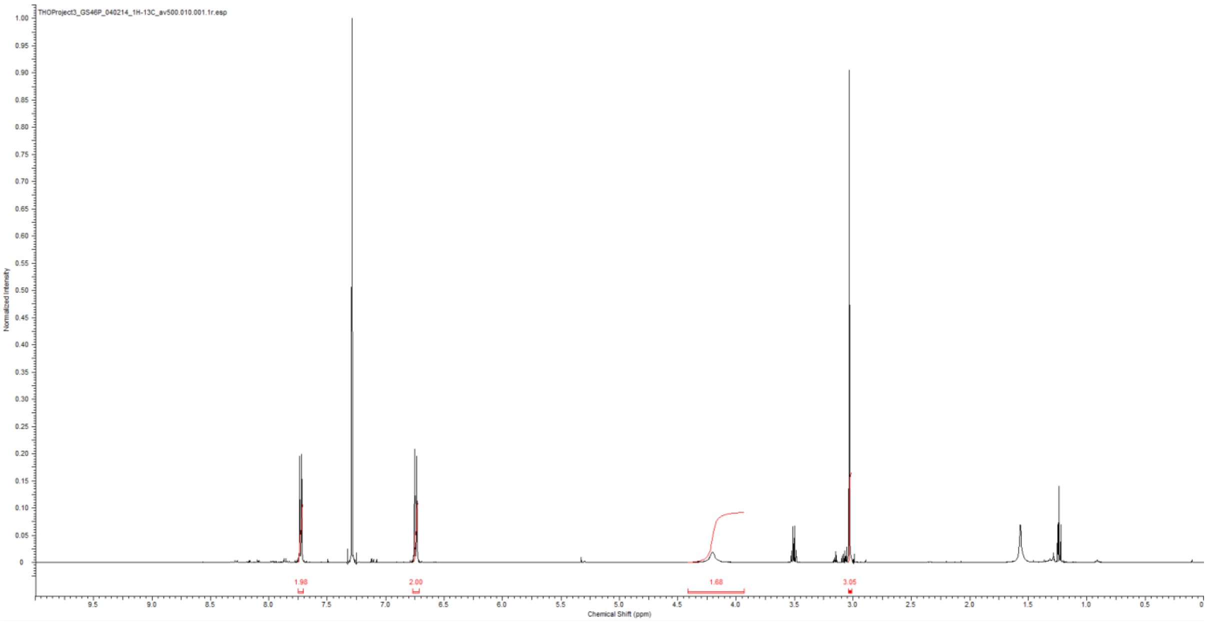

126 MHz

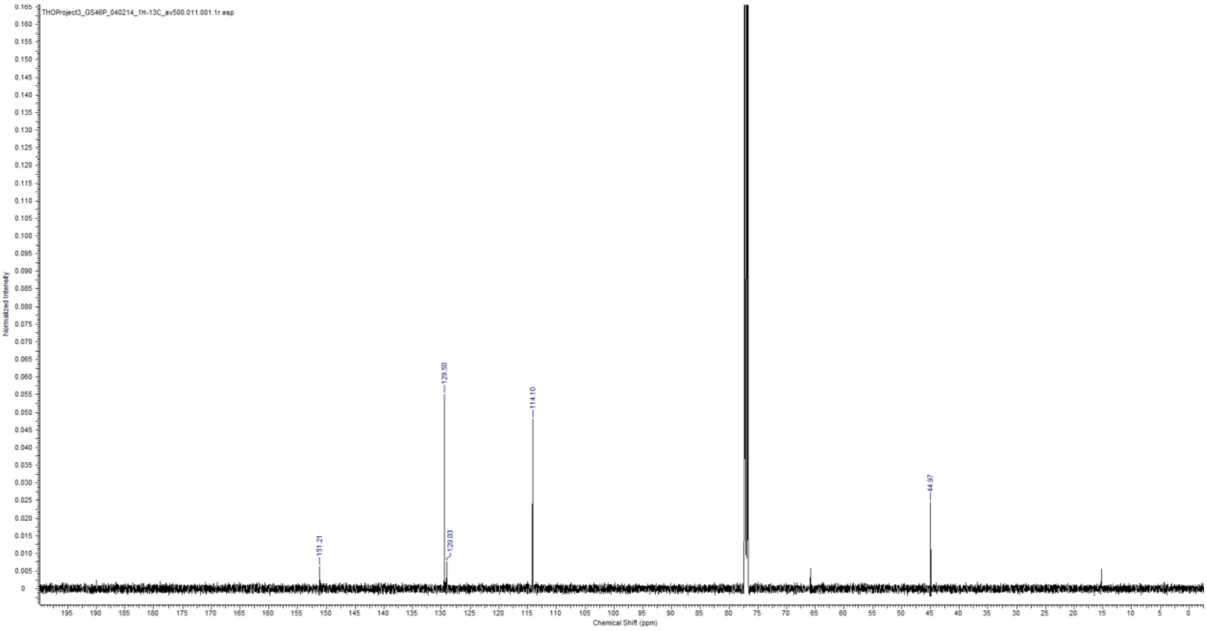

4-Aminobenzonitrile hydrochloride (6v)

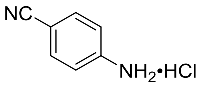

Isolated product: 600 MHz

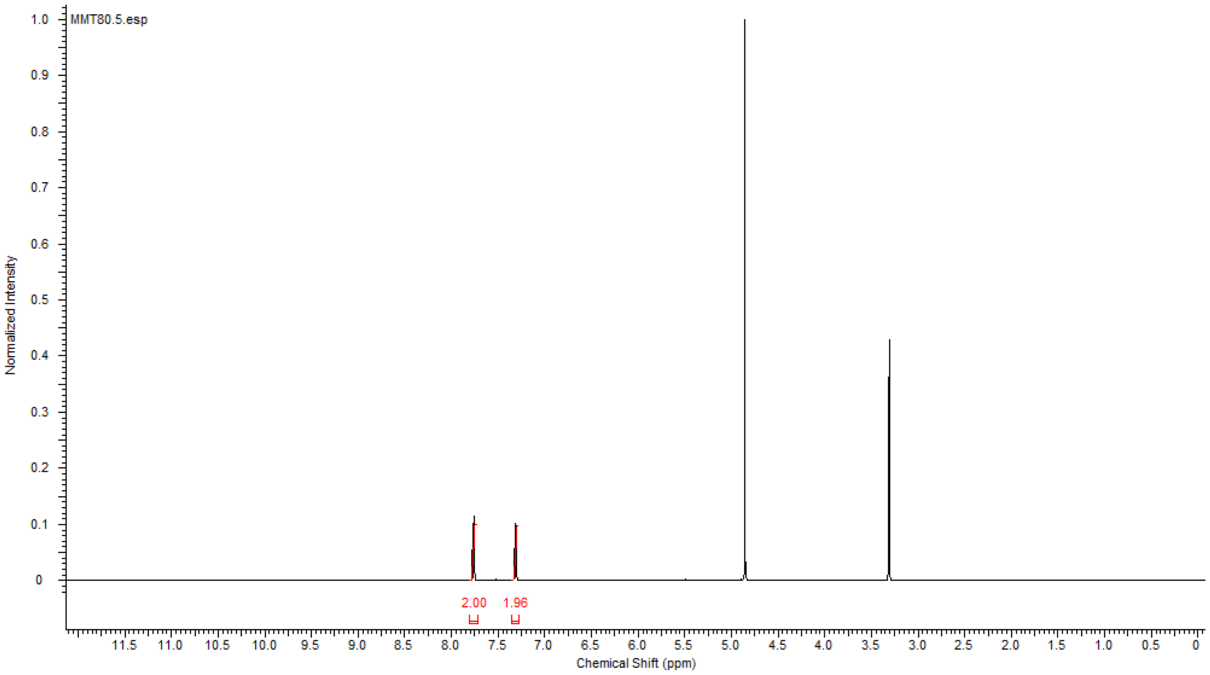

150 MHz

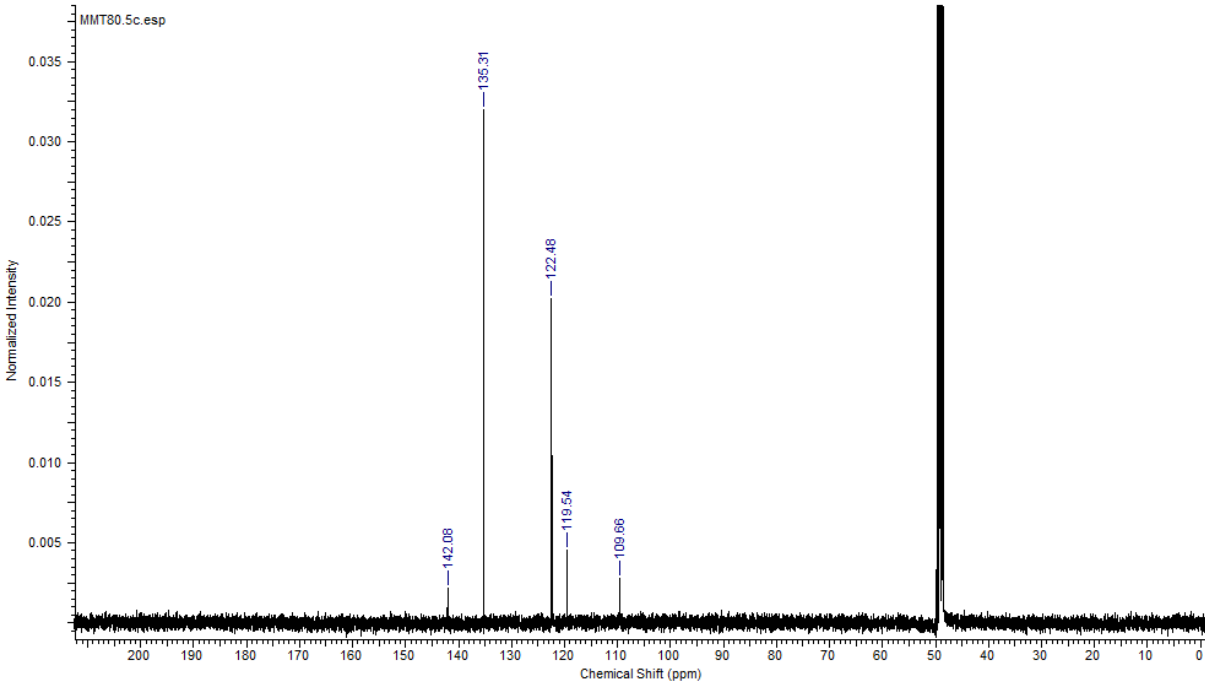

8-Aminoquinoline (6w)

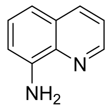

Isolated product: 500 MHz

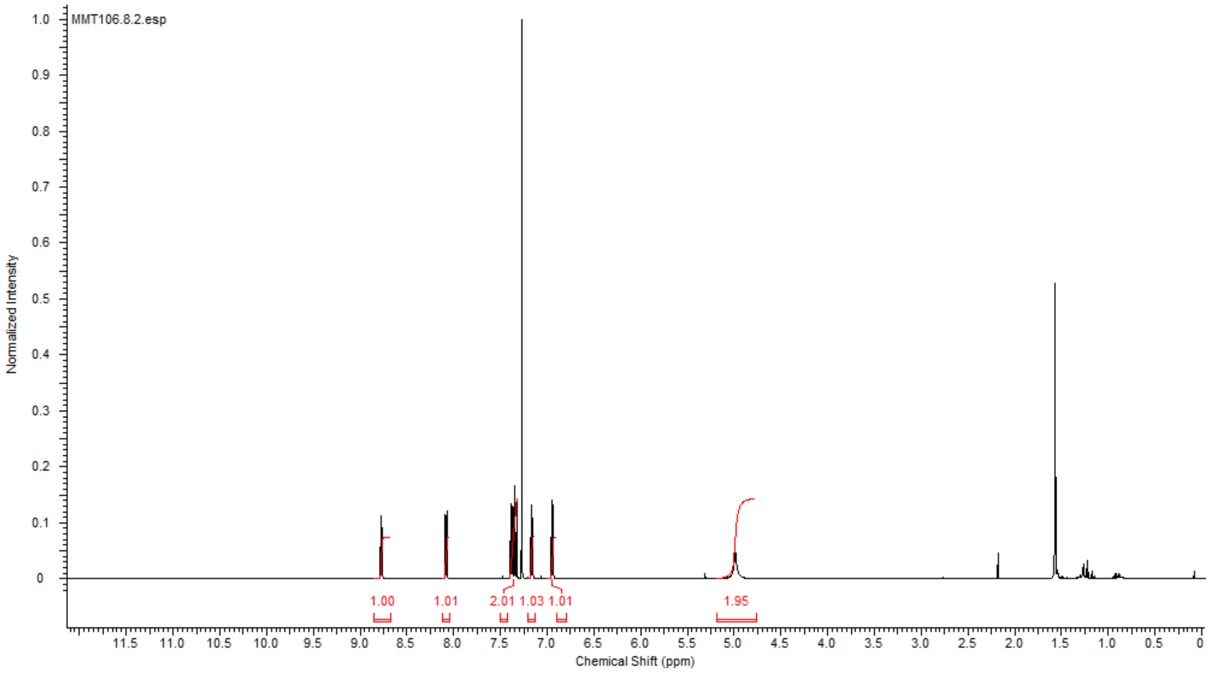

126 MHz

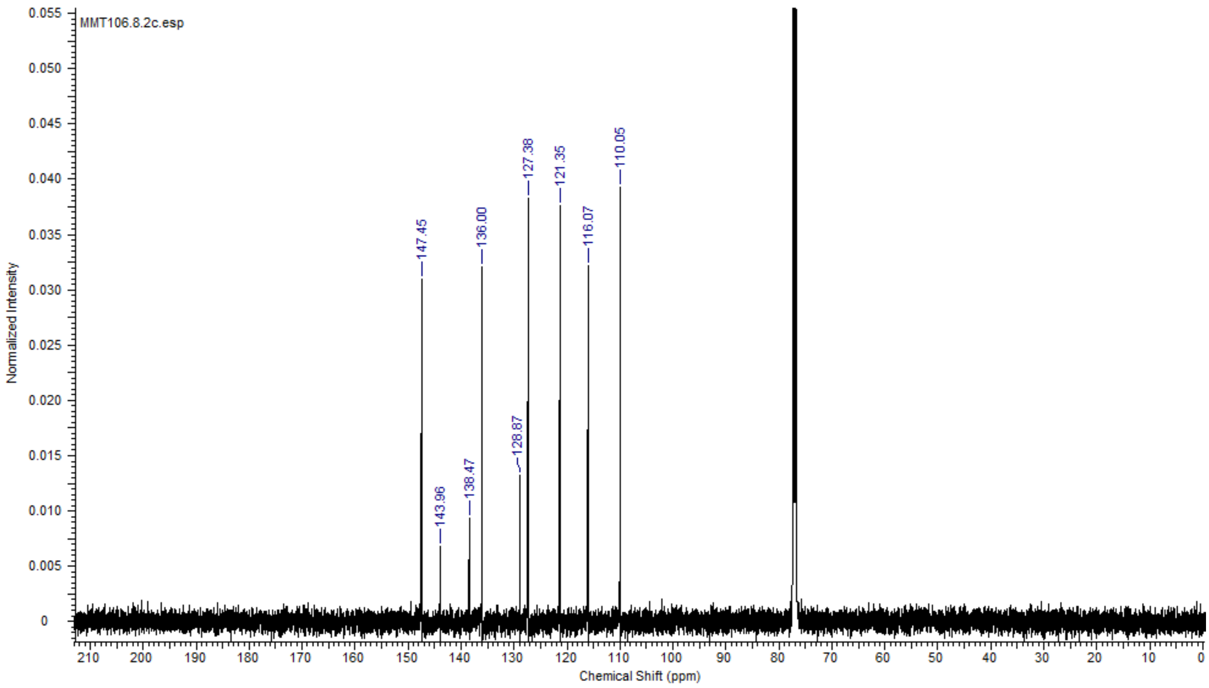

2-Methyl-6-benzoxazolamine (6x)

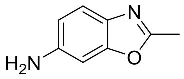

Isolated product: 500 MHz

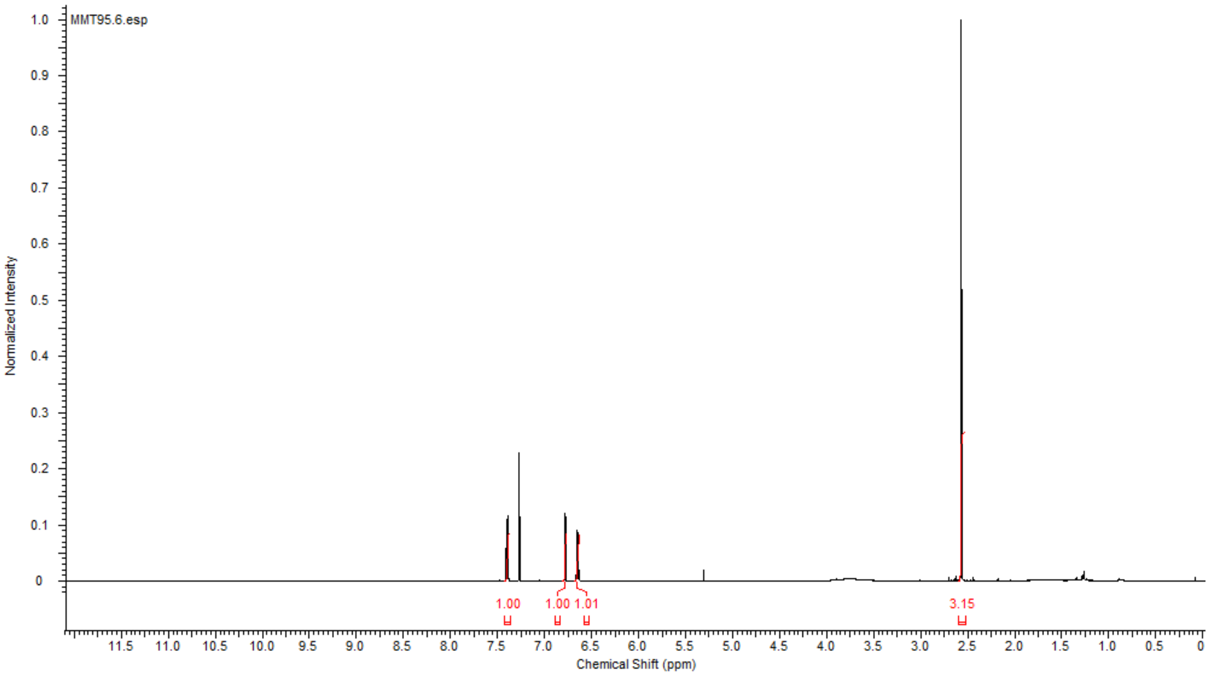

126 MHz

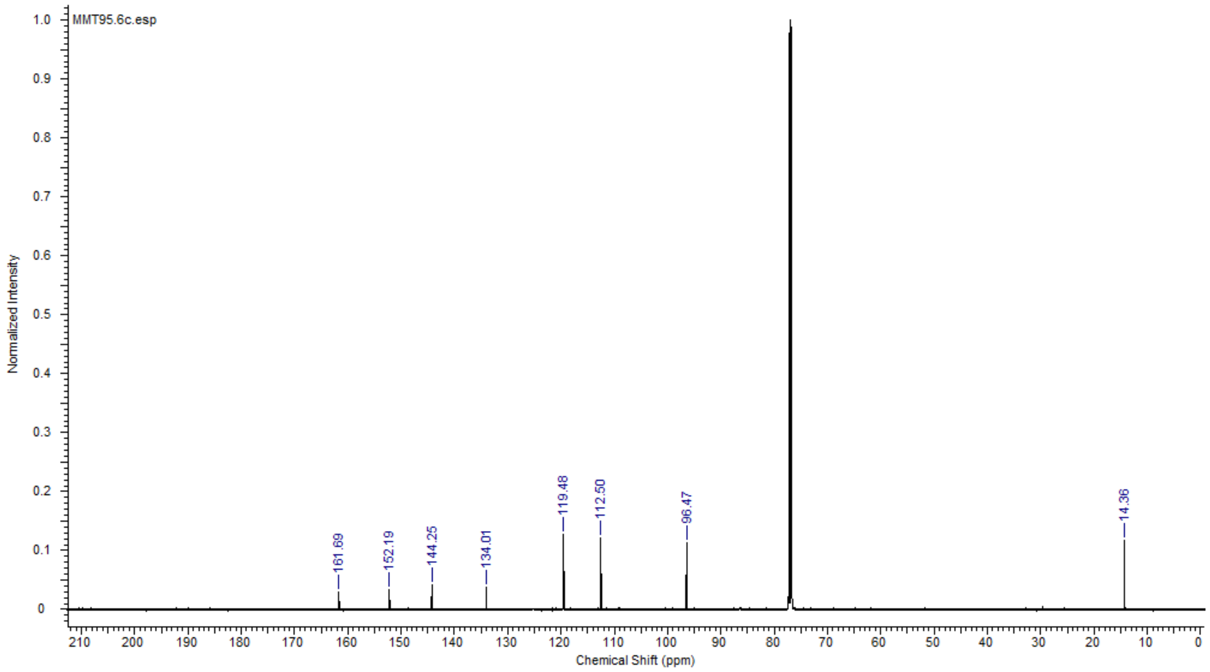

2-Methyl-6-benzothiazolamine (6y)

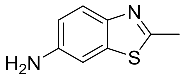

Isolated product: 500 MHz

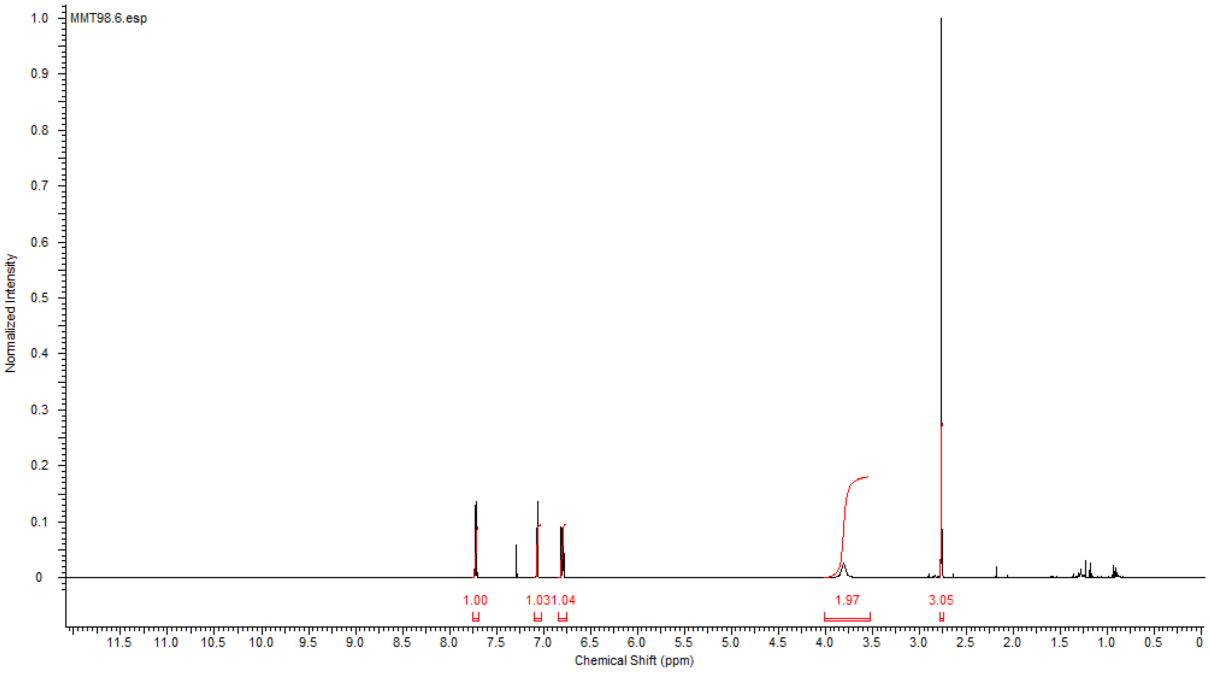

126 MHz

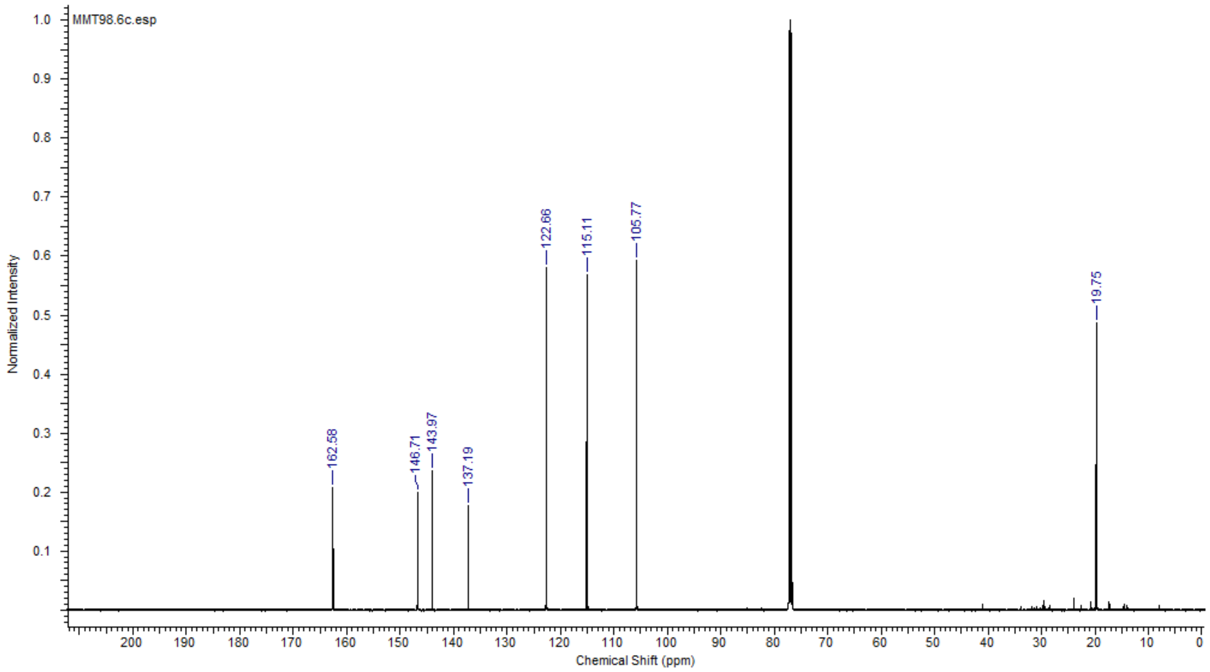

Deuterium Labelling Experiments (NMRs of crude products)

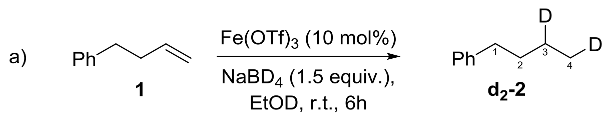

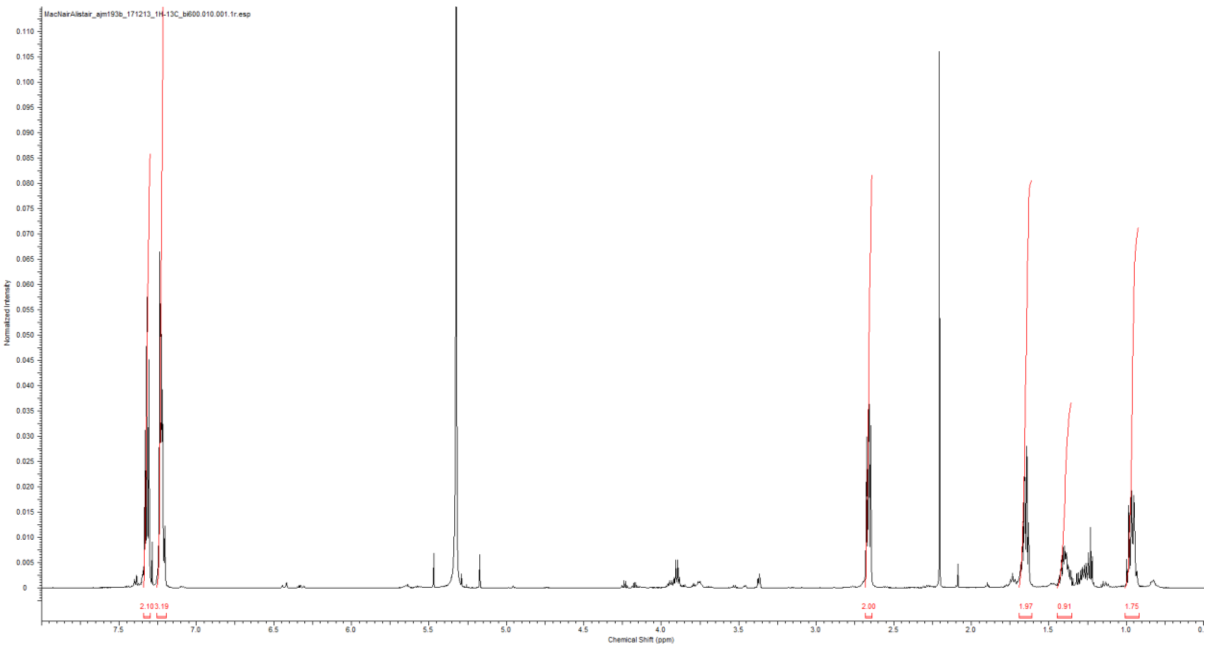

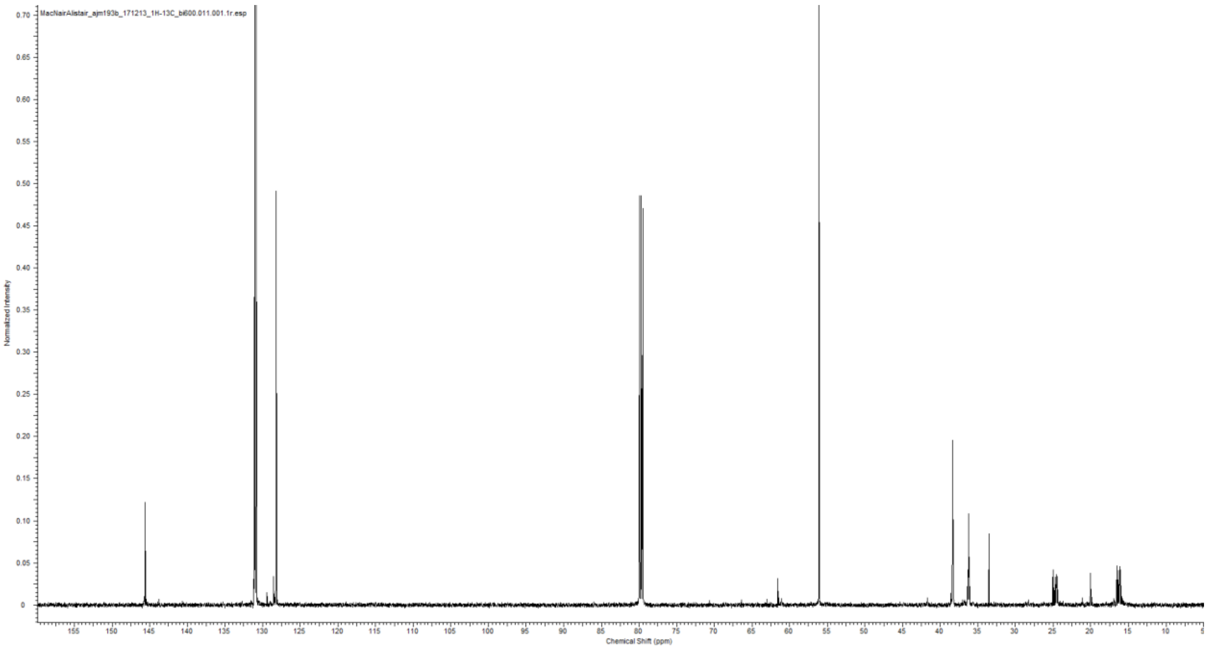

b)

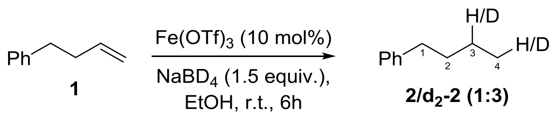

Ph
1
Fe(OTf)$_3$ (10 mol%)
NaBD$_4$ (1.5 equiv.),
EtOH, r.t., 6h
H/D
H/D
Ph
1
2
3
4
2/d$_2$-2 (1:3)

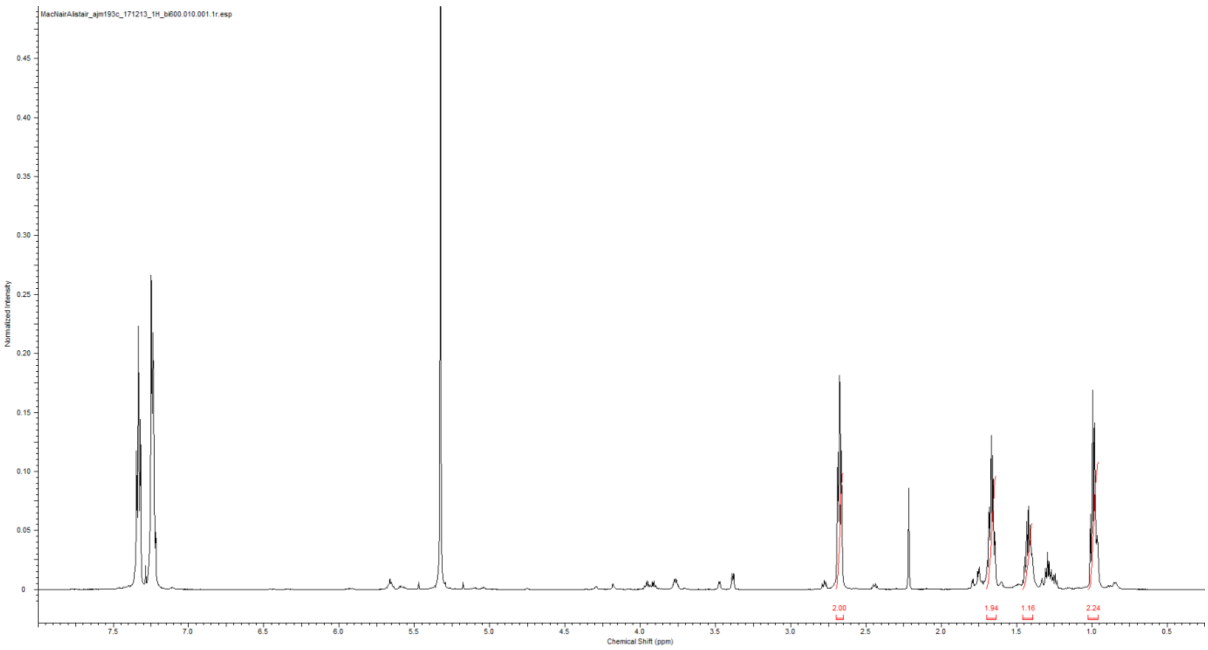

MacNairAlistair_ajm193c_171213_1H_bi600.010.001.1r.esp
Normalized Intensity
Chemical Shift (ppm)
2.00
1.94
1.16
2.24

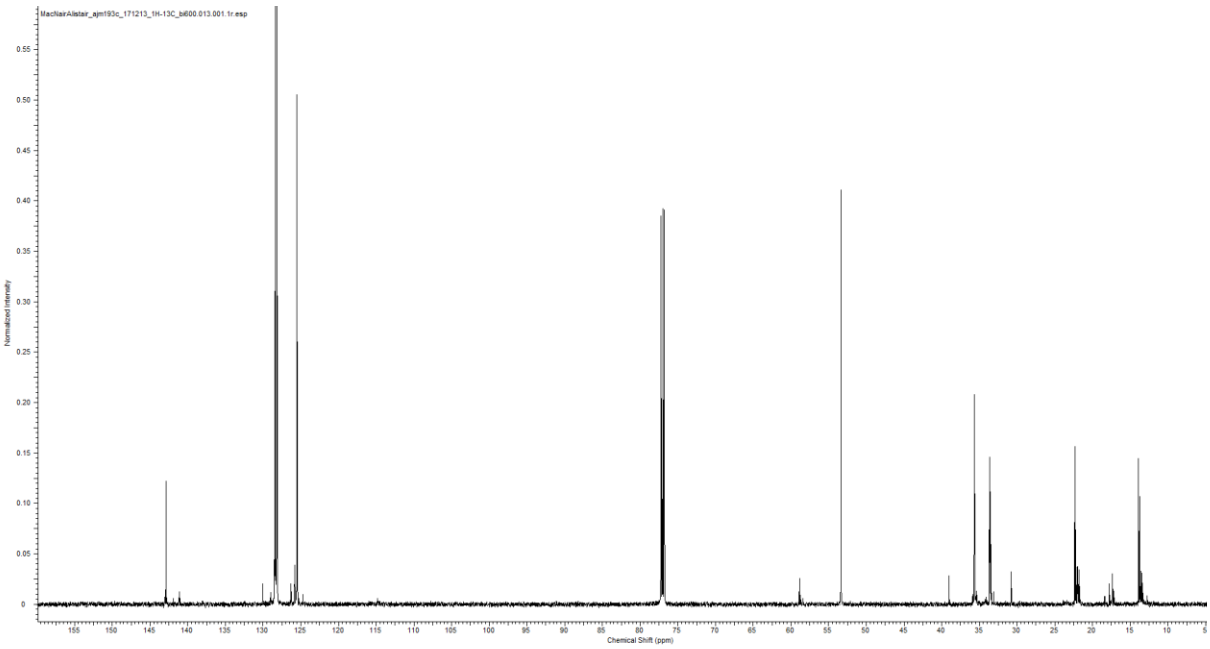

MacNairAlistair_ajm193c_171213_1H-13C_bi600.013.001.1r.esp
Normalized Intensity
Chemical Shift (ppm)

c)

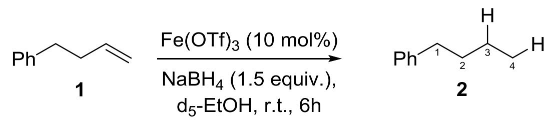
Ph
1
Fe(OTf)$_3$ (10 mol%)
NaBH$_4$ (1.5 equiv.),
d$_5$-EtOH, r.t., 6h
H
H
Ph
1
2
3
4
2

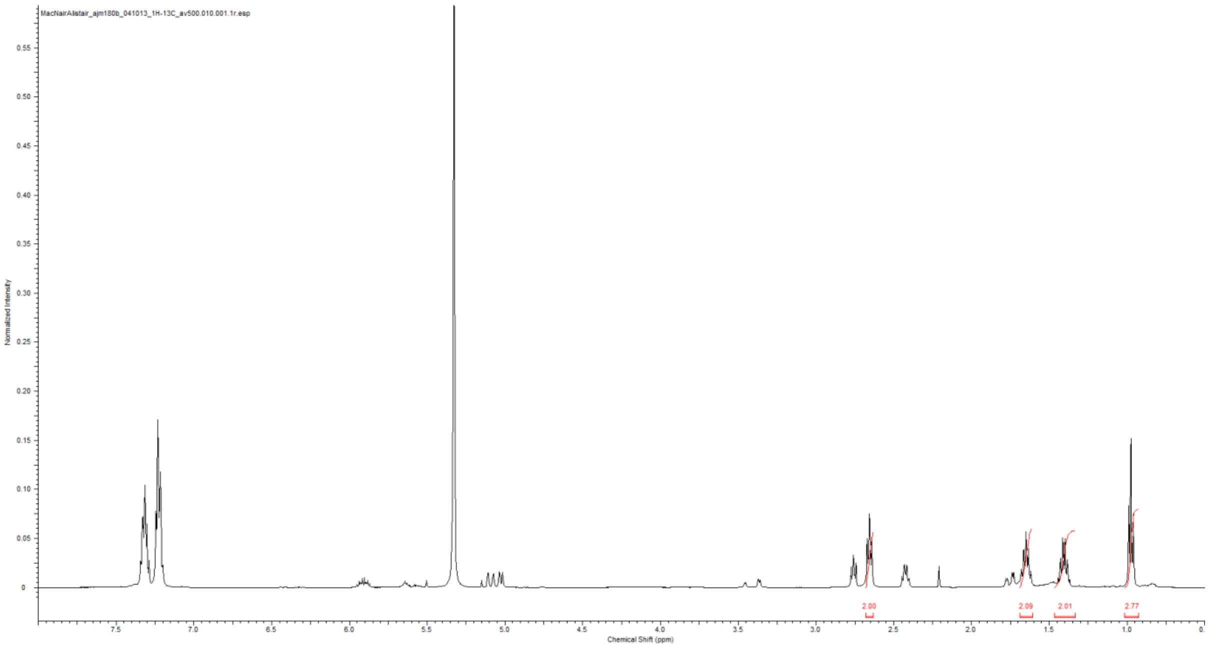
MacNairAlistair_ajm180b_041013_1H-13C_av500.010.001.1r.esp
Normalized Intensity
Chemical Shift (ppm)
2.00
2.09
2.01
2.77

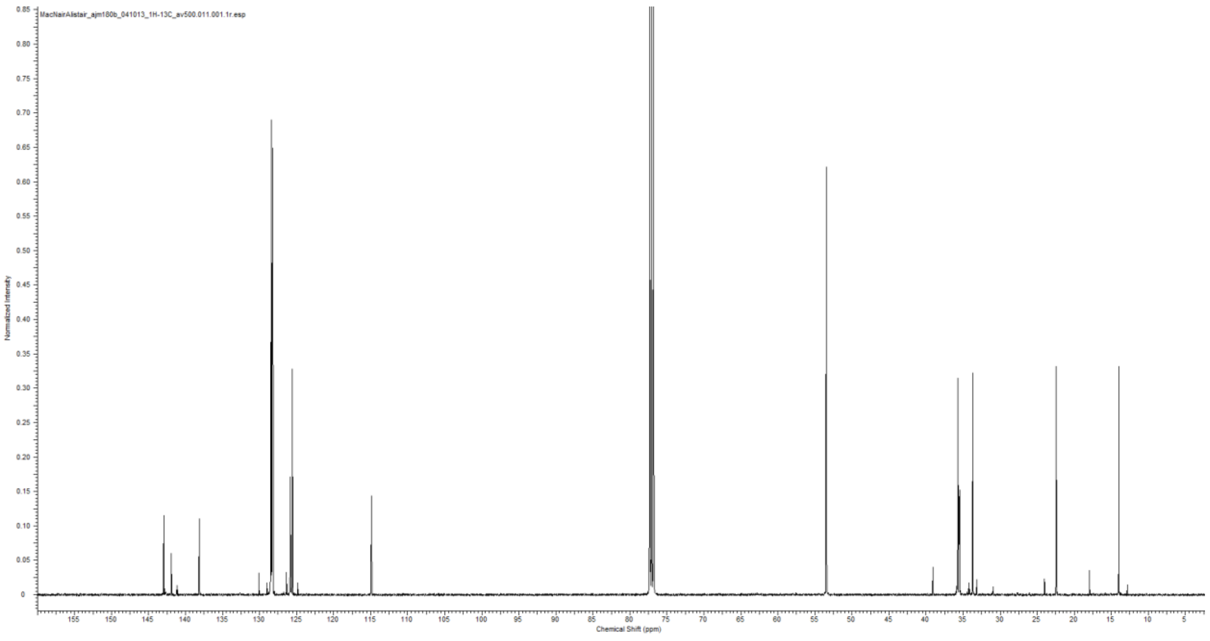
MacNairAlistair_ajm180b_041013_1H-13C_av500.011.001.1r.esp
Normalized Intensity
Chemical Shift (ppm)

References

[1] M. D. Greenhalgh, S. P. Thomas, *Chem. Commun.*, 2013, **49**, 11230.
[2] J. Seayad, A. M. Seayad, C. L. L. Chai, *Org. Lett.*, 2010, **12**, 1412.
[3] S. Hok, N. E. Schore, *J. Org. Chem.*, 2006, **71**, 1736.
[4] X.-F. Wu, C. Darcel, *Eur. J. Org. Chem.*, 2009, **2009**, 1144.
[5] K. Ekoue-Kovi, C. Wolf, *Org. Lett.*, 2007, **9**, 3429.

[6] C. Yang, Q. Jin, H. Zhang, J. Liao, J. Zhu, B. Yu, J. Deng, *Green Chem.*, 2009, **11**, 1401
[7] M. V. Troutman, D. H. Appella, S. L. Buchwald, *J. Am. Chem. Soc.*, 1999, **121**, 4916.
[8] S. L. Buchwald, C. Bolm, *Angew. Chem. Int. Ed.*, 2009, **48**, 5586.
[9] E. C. Ashby, J. J. Lin, *J. Org. Chem.*, 1978, **43**, 2567.
[10] T. S. Carter, L. Guiet, D. J. Frank, J. West, S. P. Thomas, *Adv. Synth. Catal.*, 2013, **355**, 880.
[11] P. S. Fier, J. F. Hartwig, *J. Am. Chem. Soc.*, 2012, **134**, 10795.
[12] Y. Nakao, M. Takeda, T. Matsumoto, T. Hiyama, *Angew. Chem., Int. Ed. Engl.*, 2010, **49**, 4447.
[13] X. Shen, A. M. Hyde, S. L. Buchwald, *J. Am. Chem. Soc.*, 2010, **132**, 14076.
[14] M. Karabacak, Z. Cinar, M. Kurt, S. Sudha, N. Sundaraganesan, *Spectrochim. Acta, Part A*, 2012, **85**, 179-189.
[15] A. Das, R. Chaudhuri, R.-S. Liu, *Chem. Commun.*, 2009, 4046.
[16] S. Chandrasekhar, S. J. Prakash, C. L. Rao, *J. Org. Chem.*, 2006, **71**, 2196.
[17] L. Pehlivan, E. Métay, S. Laval, W. Dayoub, P. Demonchaux, G. Mignani, M. Lemaire, *Tetrahedron Lett.*, 2010, **51**, 1939.
[18] G. D. Vo, J. F. Hartwig, *J. Am. Chem. Soc.*, 2009, **131**, 11049.
[19] D.-Y. Lee, J. F. Hartwig, *Org. Lett.*, 2005, **7**, 1169.
[20] Y. Motoyama, K. Kamo, H. Nagashima, *Org. Lett.*, 2009, **11**, 1345.
[21] T. T. Dang, Y. Zhu, J. S. Y. Ngiam, S. C. Ghosh, A. Chen, A. M. Seayad, *ACS Catalysis*, 2013, **3**, 1406.
[22] C.-X. Wei, L.-P. Guan, J.-H. Jia, K.-Y. Chai, Z.-S. Quan, *Arch. Pharm. Res.*, 2009, **32**, 23.
[23] P. Hrobárik, I. Sigmundová, P. Zahradník, *Synthesis*, 2005, **2005**, 600.
